# Identification and characterisation of a rare *MTTP* variant underlying hereditary non-alcoholic fatty liver disease

**DOI:** 10.1016/j.jhepr.2023.100764

**Published:** 2023-04-23

**Authors:** Jane I. Grove, Peggy C.K. Lo, Nick Shrine, Julian Barwell, Louise V. Wain, Martin D. Tobin, Andrew M. Salter, Aditi N. Borkar, Sara Cuevas-Ocaña, Neil Bennett, Catherine John, Ioanna Ntalla, Gabriela E. Jones, Christopher P. Neal, Mervyn G. Thomas, Helen Kuht, Pankaj Gupta, Vishwaraj M. Vemala, Allister Grant, Adeolu B. Adewoye, Kotacherry T. Shenoy, Leena K. Balakumaran, Edward J. Hollox, Nicholas R.F. Hannan, Guruprasad P. Aithal

**Affiliations:** 1 National Institute of Health Research (NIHR) Nottingham Biomedical Research Centre, Nottingham University Hospitals NHS Trust & University of Nottingham, Nottingham, UK; 2 Nottingham Digestive Diseases Centre, Translational Medical Sciences, School of Medicine, University of Nottingham, Nottingham, UK; 3 Translational Medical Sciences, School of Medicine, University of Nottingham, Nottingham, UK; 4 University of Nottingham Biodiscovery Institute, University of Nottingham, Nottingham, UK; 5 Genetic Epidemiology Group, Department of Population Health Sciences, University of Leicester, Leicester, UK; 6 Clinical Genetics Department, University Hospitals Leicester NHS Trust, Leicester, UK; 7 NIHR Leicester Respiratory Biomedical Research Centre, Glenfield Hospital, Leicester, UK; 8 School of Biosciences, University of Nottingham, Nottingham, UK; 9 School of Veterinary Medicine and Science, University of Nottingham, Nottingham, UK; 10 Leicester Cancer Research Centre, University of Leicester, Leicester, UK; 11 Ulverscroft Eye Unit, Department of Neuroscience, Psychology and Behaviour, University of Leicester, Leicester, UK; 12 Department of Chemical Pathology and Metabolic Diseases, University Hospitals of Leicester NHS Trust, Leicester, UK; 13 Department of Cardiovascular Sciences, University of Leicester, Leicester, UK; 14 Department of Gastroenterology, University Hospitals of Leicester NHS Trust, Leicester, UK; 15 Department of Genetics and Genome Biology, University of Leicester, Leicester, UK; 16 Population Health and Research Institute, Trivandrum, India

**Keywords:** Microsomal triglyceride transfer protein, Abetalipoproteinaemia, hiPSC-derived hepatocytes, Lipoprotein ApoB

## Abstract

**Background & Aims:**

Non-alcoholic fatty liver disease (NAFLD) is a complex trait with an estimated prevalence of 25% globally. We aimed to identify the genetic variant underlying a four-generation family with progressive NAFLD leading to cirrhosis, decompensation, and development of hepatocellular carcinoma in the absence of common risk factors such as obesity and type 2 diabetes.

**Methods:**

Exome sequencing and genome comparisons were used to identify the likely causal variant. We extensively characterised the clinical phenotype and post-prandial metabolic responses of family members with the identified novel variant in comparison with healthy non-carriers and wild-type patients with NAFLD. Variant-expressing hepatocyte-like cells (HLCs) were derived from human-induced pluripotent stem cells generated from homozygous donor skin fibroblasts and restored to wild-type using CRISPR-Cas9. The phenotype was assessed using imaging, targeted RNA analysis, and molecular expression arrays.

**Results:**

We identified a rare causal variant c.1691T>C p.I564T (rs745447480) in *MTTP*, encoding microsomal triglyceride transfer protein (MTP), associated with progressive NAFLD, unrelated to metabolic syndrome and without characteristic features of abetalipoproteinaemia. HLCs derived from a homozygote donor had significantly lower MTP activity and lower lipoprotein ApoB secretion than wild-type cells, while having similar levels of *MTP* mRNA and protein. Cytoplasmic triglyceride accumulation in HLCs triggered endoplasmic reticulum stress, secretion of pro-inflammatory mediators, and production of reactive oxygen species.

**Conclusions:**

We have identified and characterised a rare causal variant in *MTTP*, and homozygosity for *MTTP* p*.*I564T is associated with progressive NAFLD without any other manifestations of abetalipoproteinaemia. Our findings provide insights into mechanisms driving progressive NAFLD.

**Impact and Implications:**

A rare genetic variant in the gene *MTTP* has been identified as responsible for the development of severe non-alcoholic fatty liver disease in a four-generation family with no typical disease risk factors. A cell line culture created harbouring this variant gene was characterised to understand how this genetic variation leads to a defect in liver cells, which results in accumulation of fat and processes that promote disease. This is now a useful model for studying the disease pathways and to discover new ways to treat common types of fatty liver disease.

## Highlights

• A rare *MTTP* variant, p.I564T, leads to progressive fatty liver disease and cirrhosis.
• Hepatocyte-like cells expressing MTP 564T have reduced liver lipoprotein secretion capability.
• Reactive oxygen species, NF-κB pathway, and extracellular matrix remodelling drive lipotoxicity.

## Graphical abstract

**Figure:**
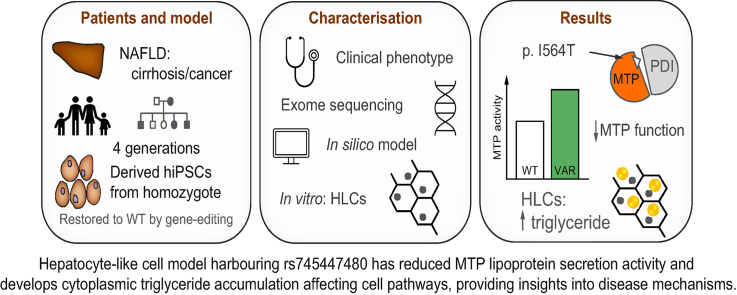

## Introduction

Non-alcoholic fatty liver disease (NAFLD) is a complex trait encompassing a spectrum of accumulation of triglyceride-rich lipid droplets within the hepatocytes (steatosis), non-alcoholic steatohepatitis (NASH; having ballooning degeneration and inflammatory cell infiltration), varying degrees and patterns of fibrosis leading to cirrhosis and its decompensation, and hepatocellular carcinoma (HCC). With rising incidence of obesity and type 2 diabetes, NAFLD is now the most common chronic liver disease, with an estimated 25% population prevalence globally.^1^

Genome-wide association studies (GWAS) have identified a number of genetic risk variants for NAFLD, including *PNPLA3* rs738409 and *TM6SF2* rs58542926, both of which have robust associations with disease phenotypes via functional pathobiological pathways.^2^^,^^3^ Accretion of the *PNPLA3* variant on lipid droplets sequesters coactivators, resulting in reduced lipolysis and lipophagy, and the *TM6SF2* variant impairs VLDL lipidation. Accumulation of triglycerides in both contexts is associated with progressive liver disease.^2^

Microsomal triglyceride transfer protein (MTP) as a heterodimer with protein disulfide isomerase (PDI) catalyses lipidation and assembly of apolipoprotein B (ApoB)-containing lipoproteins for secretion by hepatocytes, and *MTTP* variants have been linked with susceptibility to NAFLD.^4^^,^^5^ Rare, loss-of-function mutations in *MTTP* can result in the recessive disorder abetalipoproteinaemia,^6^ where MTP deficiency causes defective lipoprotein biosynthesis having multiple severe effects including liver steatosis and fibrosis.^5^^,^^7^ However, hereditary progressive NAFLD associated with a *MTTP* variant, without any manifestations of abetalipoproteinaemia, has not been previously described.

Here we have clinically characterised a large four-generation family found to have a rare *MTTP* variant located at the interface with PDI resulting in progressive NAFLD, with consequent cirrhosis, liver failure, and HCC in homozygotes. We evaluated post-prandial metabolic responses in carriers of the novel *MTTP* p.I564T variant compared with non-carriers. We used hepatocyte-like cells (HLCs) derived from human-induced pluripotent stem cells (hiPSCs) generated from donor skin fibroblasts from carriers and non-carriers of the *MTTP* variant, as a stable reproducible model for understanding the effect of the variant on the cellular phenotype. This has enabled us to understand how disrupted hepatic lipid homoeostasis can drive steatosis and NAFLD and therefore link genotype to phenotype in hereditary NAFLD.

## Patients and methods

Further details of methods used are available in Supplementary information.

### Human samples

The clinical studies were approved by the Health Research Authority after review by the National Research Ethics Service: East Midlands Northampton Committee for the Genetics of Rare Inherited Disorders (GRID) study (Ref. 12/EM/0262) and North-East Committee for meal-response study (Ref. 16/NE/0251). Studies were conducted according to the Declaration of Helsinki (Hong Kong Amendment) and Good Clinical Practice (European guidelines). All participants provided written informed consent. For the meal-response analysis, participants were recruited to the study at Queens Medical Centre, Nottingham University Hospitals, between 1 November 2016 and 1 June 2017. Patients with biopsy-proven NAFLD, sex and age matched (within 10 years) to family members, were consecutively identified from a large secondary care cohort who had previously participated in research, and invited to participate. Healthy volunteers were similarly identified and invited. None had diabetes or hazardous alcohol intake and had no known liver disease and had circulating caspase-cleaved CK18 level below 99 U/L.

Clinical investigations followed standard clinical care and included 6-month follow-up as required. Variants segregating with disease were identified following exome sequencing (llumina HiSeq2000, San Diego, CA, USA). Genotype determination was done using Sanger sequencing (Source Bioscience Ltd, Nottingham, UK) or PCR restriction fragment analysis.

### *In silico* analysis

Models of MTP were based on Protein Data Bank sequence 617S^8^ and visualised using Visual Molecular Dynamics software (University of Illinois Urbana-Champaign, Champaign, IL, USA).^9^

### Metabolite and protein analyses

Serum cholesterol, triglycerides, and ApoB, and plasma glucose were quantified using calibrated Horiba auto-analyser and reagents following validated standard manufacturer protocols (Horiba ABX, Montpellier, Hérault, France) at the University of Nottingham Metabolic Analysis Facility. Serum insulin was quantified using Human Insulin specific RAI kit (Merck KGaA, Darmstadt, Germany). Plasma lipoproteins were separated by sequential non-equilibrium density-gradient ultracentrifugation.

Apolipoprotein B-100 (ApoB-100) was determined in culture supernatants by ELISA (Merck KGaA, Darmstadt, Germany) in duplicate (twice). MTP activity was determined in lysed cells (in triplicate) at four dilutions using MTP Activity Assay Kit (Merck KGaA). Human NF-κB Pathway, Phospho-Kinase, and XL Cytokine Array Kits (R&D Systems, Minneapolis, Minnesota, USA) were used to determine protein expression or secretion.

### Fibroblast reprogramming, hiPSC maintenance, and differentiation

Two 2-mm skin punch biopsies were obtained from study participants, and primary dermal fibroblasts were established and skin fibroblasts were reprogrammed using CytoTune iPS 2.0 Sendai Reprogramming Kit (Invitrogen, Thermo Fisher Scientific, Waltham, MA, USA) in accordance with the manufacturer’s guidelines. Mesoderm, ectoderm, and hepatocyte differentiation of hiPSCs was as described previously.[10], [11], [12]

### CRISPR-Cas9-mediated correction of I564T mutation in the *MTTP*^*(VAR/VAR)*^

For CRISPR-Cas9 editing, single-guide RNA (gaacatcctgctgtctactg) was cloned and nucleofected (Lonza, Basel, Basel-Stadt, Switzerland) into the *MTTP*^*(VAR/VAR)*^ parental line.^13^ Clones were screened to select one with corrected alleles. hiPSCs derived from clones with corrected allele *MTTP*^*(WT*^*^∗^*^*/WT*^*^∗^*^*)*^, and the parental line was differentiated to HLCs in parallel for characterisation.

### Imaging and analysis of mitochondrial function and cellular reactive oxygen species

Cells were stained using Nile red, Hoechst, DAPI, or antibodies. The mitochondrial content of HLCs was visualised using 100 nM MitoTracker Green FM or MitoTracker Deep Red FM (Invitrogen, Thermo Fisher Scientific, Waltham, MA, USA), and intracellular reactive oxygen species (ROS) and mitochondrial superoxide production was assessed using 2.5 μM CellROX Green or 2.5 μM MitoSox Red. Mitochondrial respiration was determined using the Seahorse XF96 analyser (Seahorse Bioscience, Agilent Technologies Inc., Santa Clara, CA, USA).

### Gene expression analysis and RNA sequencing

Quantitative real-time PCR was carried out as described in Supplementary methods. Fold changes in expression were calculated using the comparative ΔΔCt method standardised against the housekeeping gene porphobilinogen deaminase (*PBGD*), and the mean of Ct values ± SE was reported.^12^ RNA sequencing and bioinformatics analysis were performed at the Babraham Institute (Cambridge, UK).

### Statistical analysis

Statistical analyses were performed using GraphPad Prism version 8 (San Diego, CA, USA) software. One-way ANOVA followed by Dunnett’s multiple comparison test were used to compare data from samples grouped by a single factor. Student’s *t* test was used to compare the means of variables determined in two groups.

## Results

### Clinical presentation of family

**Fig. 1:**
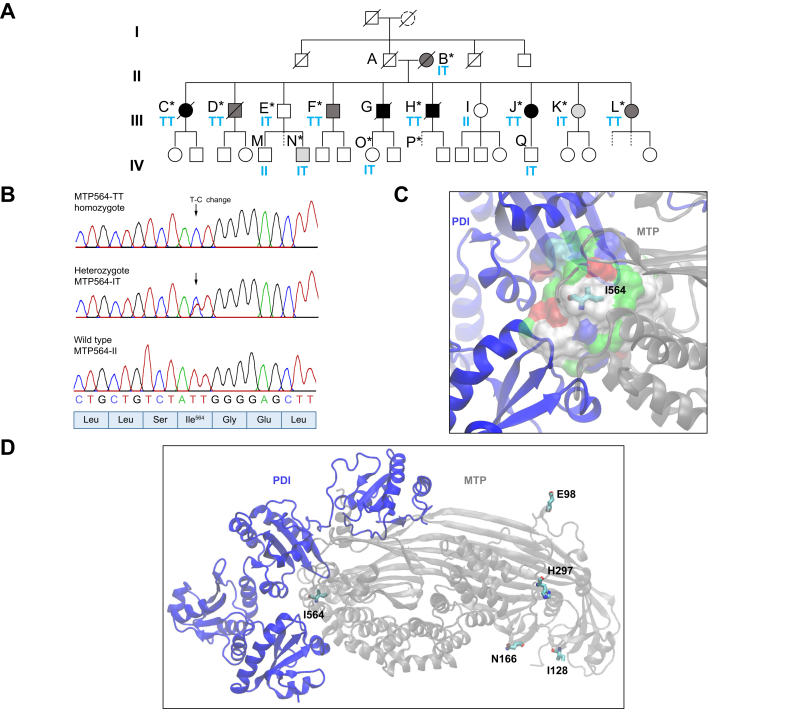
Identification of a pathogenic variant in a large family with non-alcoholic fatty liver disease. (A) Pedigree. Clinical features are described in Table 1. Diagnosis is indicated by shading: black, hepatocellular carcinoma; dark grey, cirrhosis; light grey, non-alcoholic steatohepatitis. Dashed lines indicate no investigations. ∗Exome sequenced. Blue letters indicate residue 564 in MTP. (B) Variant rs745447480 sequencing. (C) Local environment of I564 on MTP–PDI interface (hydrophobic pocket [white], polar [green], and charged [red/blue] residues). (D) 564T variant and common non-synonymous variants (side chains: C = cyan; O = red; N = blue) in a model derived from PDB ID:617S,^8^ a heterodimer of PDI (blue), and *MTTP* gene product (MTP) (grey). MTP, microsomal triglyceride transfer protein; PDI, protein disulfide isomerase.

A British four-generation family with recent Indian ancestry (Fig. 1A) was referred for genetic counselling after three individuals from the same generation developed HCC. Parent A had no history of NAFLD or metabolic syndrome; Parent B presented with NAFLD symptoms aged 80 and was diagnosed with cirrhosis aged 87. Clinical investigations found that all 10 children had NAFLD diagnosis as adults (C–L in Table 1) with progression to NASH, cirrhosis (in seven), and HCC (in four), suggesting a high conversion rate between NAFLD to cirrhosis and NAFLD to HCC. Only one of the affected individuals had a BMI of >30, and instances of type 2 diabetes, hypertension, or hyperlipidaemia within the family were not linked with the presence or severity of disease.

**Table 1:** **Clinical characteristics of family members**.

Person (Fig. 1A)	*MTTP* p.Ile564Thr	Sex (male/female)	Liver disease diagnosis (method)	Age diagnosed (years)	Type 2 diabetes	BMI	Hypertension	Liver biochemistry and lipid blood analyses at diagnosis	Other subsequent investigations, treatments, and comorbidities	*PNPLA3* p.I148M	*TM6SF2* p.E167 K	*MTTP* p.I128T	*MTTP* p.Q297H
B	IT	F	Cirrhosis and ascites (USS)	88	x	<30	✓	ALT = 26, AP = 120, Chol = 3.5, TG = 1.29	Cardiovascular-related death aged 90	MM	EK	TT	HH
C	TT	F	HCC and cirrhosis (CT/biopsy)	57	✓	<30	✓	ALT = 38, AP = 129, normal lipids	Liver resection ablation, right hemihepatectomy, HCC recurred; liver-related death	IM	EE	TT	HH
D	TT	M	Cirrhosis (MRI)	60	x	<30	✓	Bilirubin = 312 μmol/L, normal lipids	Liver screen = normal; died of gallbladder sepsis aged 72	IM	EE	TT	HH
E	IT	M	FL (USS)	57	✓	30	✓	ALT = 30, AP = 75, Chol = 2.5, TG = 0.75, HDL = 1.2 mmol/L, LDL = 1.0 mmol/L	Stable for 18 years: TE = 5.8 kPa, CAP = 341, ApoB = 0.94	IM	EE	nd	HH
F	TT	M	Cirrhosis (USS)	56	x	21	✓	ALT = 23, AP = 125, Chol = 2.4, TG = 0.58, HDL = 1.7 mmol/L, LDL = 0.4 mmol/L	No retinitis pigmentosa; vagotomy and pyloroplasty; mild bone marrow failure	IM	EK	TT	HH
G	T∗	M	HCC and cirrhosis (biopsy)	57	x	<30	X	Normal lipids	Multifocal HCC: chemotherapy and hepatectomy; died aged 61	nd	Nd	nd	nd
H	TT	M	HCC, cirrhosis, portal hypertension	51	x	<30	✓	Chol = 2.2, TG = 0.2, HDL = 1.6 mmol/L, LDL = 0.5 mmol/L	Liver transplant twice; died of cardiac complications aged 63	IM	EK	TT	nd
I	II	F	FL (USS)	50	x	<30	X		Diet/lifestyle changed: recovered/stable for >5 years: TE = 4.0 kPa; CAP = 223 ApoB = 1.02	IM	EE	TT	HH
J	TT	F	Cirrhosis (MRI) HCC	50 54	x	25	✓	ALT = 52, AP = 87, ApoB = 0.32, normal lipids	Liver transplant age 56: recovered/stable for >5 years: TE = 4.6 kPa, CAP = 214, BMI = 20.3; duodenal biopsy: no evidence of abetalipoproteinaemia	IM	EE	TT	HH
K	IT	F	NASH (USS) (TE:CAP = 380)	48	x	24	✓	ALT = 79, AP = 108, normal lipids, ApoB = 1.0	Diet/lifestyle changed: recovered/stable for 10 years: TE = 6.2 kPa, CAP = 298; duodenal biopsy: no evidence of abetalipoproteinaemia	IM	EK	nd	HH
L	TT	F	Cirrhosis (USS)	59	x	18	✓	Elevated ALT and AST, normal lipids	Stable for 10 years: TE = 10.3 kPa, CAP = 276, ALT = 104, AP = 87, AST = 73, cardiac arrhythmia, osteoporosis, vitamin D deficiency	IM	EE	TT	HH
M	II	M	Healthy	x	x	26	X	Normal lipids	TE = 4.6 kPa, CAP = 253, vitamin D deficiency	II	EK	IT	QH
N	IT	M	NASH (USS)	40	x	28	X	TE = 3.6 kPa, CAP=373; lipids and ALT elevated	Lifestyle and diet modified – stable for 7 years	IM	EK	nd	QH
O	IT	M	FL (USS)	38	x	27	X	Lipids elevated	Lifestyle and diet modified – recovered; stable for 19 years: TE = 5.8 kPa, CAP = 341	II	EK	IT	QH
Q	IT	M	FL (USS)	28	x	19	X	Normal lipids, duodenal Biopsy: evidence of duodenal lipid	Improved after 10 years: TE = 6.3 kPa, CAP = 199	MM	EE	IT	HH

For MTTP p.E98D, all tested were EE; for MTTP p.N166S, all tested were NN.

ALT, alanine transaminase (U/L); AP, alkaline phosphatase (U/L); ApoB, apolipoprotein B (g/L); CAP, controlled attenuation parameter (dB/m); Chol, cholesterol (mmol/L); CT, computed tomography; FL, fatty liver; HCC, hepatocellular carcinoma; MRI, magnetic resonance imaging; nd, not determined; TE, transient elastography; TG, triglycerides (mmol/L); USS, abdominal ultrasonographic steatosis score.

∗ Likely T carrier based on pedigree.

### Identification of rare MTTP variant allele associated with diagnosis

Functional variants that were unique to affected family members were identified by whole exome sequencing of 12 affected individuals (Fig. 1A) by comparison with nine unaffected South Asian controls (including spouses of F, G, H, and J; three unrelated participants from the EXCEED study;^14^ and two unrelated South Asian individuals with cholangiocarcinoma) and databases of genetic variation identified in the general population. We identified a missense variant: genomic NC_000004.12:g.99608899T>C, NM_000253.2:c.1691T>C, protein NP_000244.2:p.Ile564Thr, in *MTTP* that was unique to affected family members (Fig. 1B and C) and fully segregated with disease phenotype in those individuals analysed. All six homozygous individuals developed cirrhosis, and three also developed HCC, whereas some heterozygotes had no diagnosed disease (Table 1). The presence of both heterozygotes and homozygotes for the rare allele in the third generation of the family implies that individual A must also have carried the variant allele. The presence of fatty liver in wild-type individual I is suggested to be incidental relating to lifestyle factors.

This I564T variant has been previously described in combination with a second rare variant (IVS1+1G>C), manifesting as severe fatty liver in an atypical case of abetalipoproteinaemia in Japan^15^ but was reported to have a ‘mild effect’ in the mother carrying I564T alone. The I564T variant is described in the National Center for Biotechnology Information database^16^ as rs745447480 with allele frequency <1.6 × 10^5^ in an analysis of 251,024 alleles (gnomAD exomes v2.1.1) present in four non-Finnish European cases. The NCBI allele frequency aggregator population database reports two variants out of a 35,910 global total (both were in a European population). The GEM-Japan whole genome aggregation panel reports one allele in 15,198.^17^ Other family members were subsequently tested for this variant, and clinical features of abetalipoproteinaemia^6^ were investigated (Table 1, Table 2). Their genotype for other common functional variants at loci in *MTTP* (rs745447480, rs3816873, and rs2306985), *PNPLA3* (rs738409), and *TM6SF2* (rs58542926) associated with NAFLD was also determined. None of 83 patients with NAFLD from the Trivandrum cohort^18^ had the *MTTP* p.I564T variant allele.

**Table 2:** **Clinical characteristics of individuals investigated in generation IV**.

MTTP genotype p.I564T	Sex (male/female)	Liver disease diagnosis (method)	Age at testing (years)	Type 2 diabetes	BMI	Hypertension	Liver biochemistry and lipid blood analyses at diagnosis
IT	M	NASH (USS)	32	x	20	X	ALT = 53, AP = 68, Chol = 7, TG = 3.14, VitD <15, TE = 7.4 kPa, CAP = 352
IT	M	FL (USS)	37	x	18	X	Normal LFTs (ALT = 14), normal lipids; HbA1c = 9.5
IT	M	FL (USS)	40	x	28	X	ALT = 74, Chol = 6, TG = 2.12, TE = 3.6 kPa, CAP = 373
IT	F	Healthy (USS)	29	x	20	X	Normal LFTs (ALT = 13), normal lipids; ApoB = 0.95, TE = 3.5 kPa, CAP = 122
IT	M	Healthy (TE)	32	x	27	X	TG = 2.57, Chol = 3.7, ApoB = 1.04, TE = 5.3 kPa
IT	M	Healthy (USS)	31	x	18	X	Normal LFTs (ALT = 19), normal lipids
IT	F	Healthy (USS)	37	x	19	X	Normal LFTs (ALT = 10), Chol = 4.2, TG = 1.77, ApoB = 0.96, TE = 4.2 kPa, CAP = 291
IT	F	Healthy (USS)	39	x	21	x	Normal LFTs (ALT = 23), normal lipids
IT∗	F	Healthy (USS)	41	x	20	x	Normal LFTs (ALT = 27), Chol = 4.6, TG = 2.15, ApoB = 1.02, TE = 3.1 kPa, CAP = 231
IT∗	M	Healthy (USS)	44	x	<30	x	Normal LFTs (ALT = 27), Chol = 3.5, TG = 2.4; TE = 5.5 kPa, CAP = 281
IT	M	Healthy (USS)	49	x	<30	x	Normal LFTs (ALT = 14), Chol = 4.88, TG = 0.98, ApoB = 1.09, TE = 5.1 kPa, CAP = 245
IT	F	FL (USS) Healthy (USS)	35 38	x	21	x	Normal LFTs (ALT = 12), Chol = 6, TG=1.5, ApoB = 1.31, TE = 4.2 kPa, CAP = 325 Recovered after 3 years: TE = 6.3 kPa, CAP = 278
IT	M	FL (USS) Healthy (USS)	38 42	x	27	x	Normal LFTs (ALT = 39), Chol = 2.9, TG = 0.98 Recovered after 4 years: TE = 5.8 kPa, CAP = 243
II∗	M	FL (USS) Healthy (USS)	14 19	x	21	x	Normal LFTs (ALT = 15), normal lipids Recovered after 5 years: TE = 6.9 kPa, CAP = 216
II∗	F	Healthy (USS)	17	x	20	x	Normal LFTs (ALT = 15), normal lipids; TE = 6.4 kPa, CAP = 216
II∗	F	Healthy (USS)	20	x	<30	x	Normal LFTs (ALT = 9), normal lipids

ALT, alanine transaminase (U/L); AP, alkaline phosphatase (U/L); ApoB, apolipoprotein B (g/L) (research laboratory data); CAP, controlled attenuation parameter (dB/m); Chol, cholesterol (mmol/L); FL, fatty liver; HbA1c, haemoglobin A1c; LFT, liver function test; NASH, non-alcoholic steatohepatitis; TE, transient elastography; TG, triglycerides (mmol/L); USS, abdominal ultrasonographic steatosis score.

∗ Deduced from pedigree.

Mapping of the I564T variant onto the crystal structure^8^ located it to the heterodimer interaction interface within a hydrophobic pocket on the MTP subunit surface having surrounding polar and charged residues (Fig. 1C and D). Mutation analysis predicts that medium mutation sensitivity and substitution to threonine, a polar residue, will likely destabilise this hydrophobic pocket, promote interactions with the surrounding polar/charged residues, and thereby cause local conformational variations (PolyPhen = 1, GERP (Genome Evolutionary Rate Profiling) = 5.120, CADD (Combined Annotation-Dependent Depletion) = 20.4, Mutation Assessor = 0.76, and REVEL (Rare Exome Variant Ensemble Learner) = 0.519).^19^ A plausible impact on dimer formation and MTP functionality is therefore expected. In contrast, substitutions E98D, N166S, I128T, and H297Q arising from common single-nucleotide polymorphisms (SNPs), map to the protein surface exposed to the solvent in the complex structure (Fig. 1D), and mutation analysis predicts low mutation sensitivity and high tolerance of substitutions at each of these positions.

### Post-prandial responses in affected individuals

**Fig. 2:**
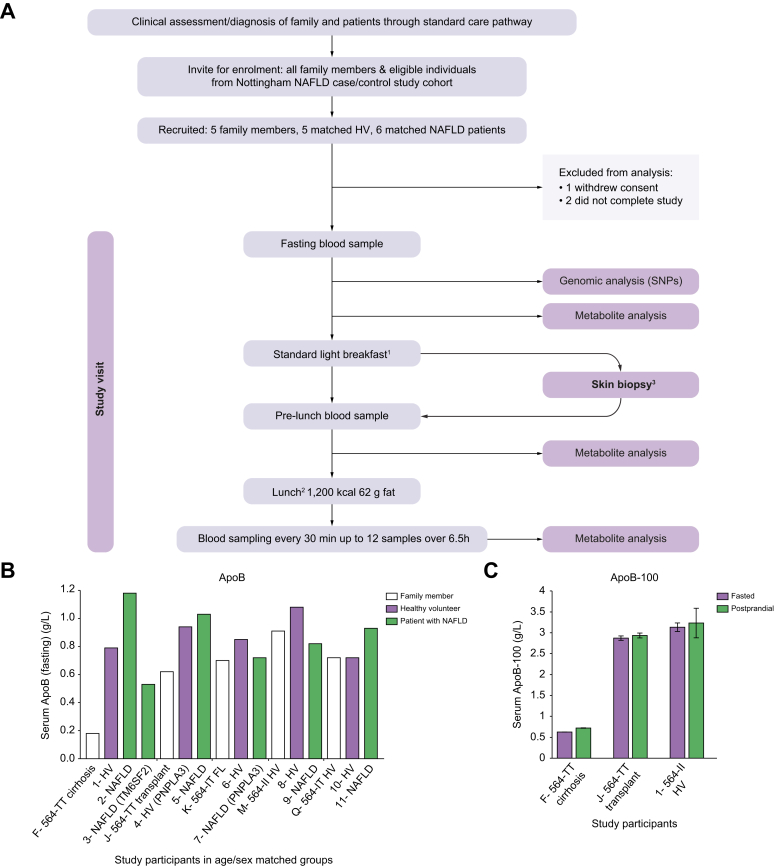
Meal-response study to investigate metabolism in family members and matched controls. (A) Study design. (B) ApoB levels. Participants are grouped according to age and sex matching to family members F, J, K, Q, and M (Table S1). MTP residue 564 is indicated (TT/TI/II). *PNPLA3* or *TM6SF2* in parentheses indicates individuals homozygous for variant rs738409 or rs58542926, respectively. (C) Serum ApoB-100 (mean levels ± standard deviation). ^1^Milk and cornflakes. ^2^80–175 min between breakfast start and pre-lunch sample. ^3^From participants J and 1 used to derive cell lines. ApoB, apolipoprotein B; ApoB-100, apolipoprotein B-100; HV, healthy volunteers; MTP, microsomal triglyceride transfer protein; NAFLD, non-alcoholic fatty liver disease; SNP, single-nucleotide polymorphism.

The phenotypic impact of the *MTTP* p.I564T variant was assessed through investigation of metabolic responses to fat consumption. Family members were invited, and responses in five participants were compared with those in age- and sex-matched healthy volunteers and patients with NAFLD (Fig. 2A and Table S1). The level of ApoB, the protein constituent assembled into chylomicron and VLDL via the activity of MTP, was notably lower in the MTP564-TT homozygote F than in other participants including two MTP564-IT heterozygotes (participants K and Q) and the MTP564-TT liver-transplant recipient J (Fig. 2B). Subsequent testing of six further heterozygotes also showed levels within the normal range. Testing of a stored, pre-transplant serum sample from individual J and clinical data revealed that levels were also markedly lower before receiving a replacement liver where the gene is likely restored.

We also compared levels of serum ApoB-100, the isoform associated with VLDL, in MTP564-TT homozygotes F and J, with those of wild-type MTP564-II control, participant 1, supporting the proposal that expression of the variant form in the liver results in reduced VLDL secretion (Fig. 2C). Levels of total cholesterol both before and after the meal were also noticeably lower in MTP564-TT homozygote F (Fig. S1) than in all other study participants owing to only very low levels of HDL-cholesterol being present (<0.4 mmol/L). Clinical data confirm this observation (Table 1), and the same phenotype was noted in another MTP564-TT homozygote, H; however, levels in homozygote J, pre-transplant, were normal. Participant F also reported post-prandial gastrointestinal discomfort and diarrhoea following the study meal.

**Fig. 3:**
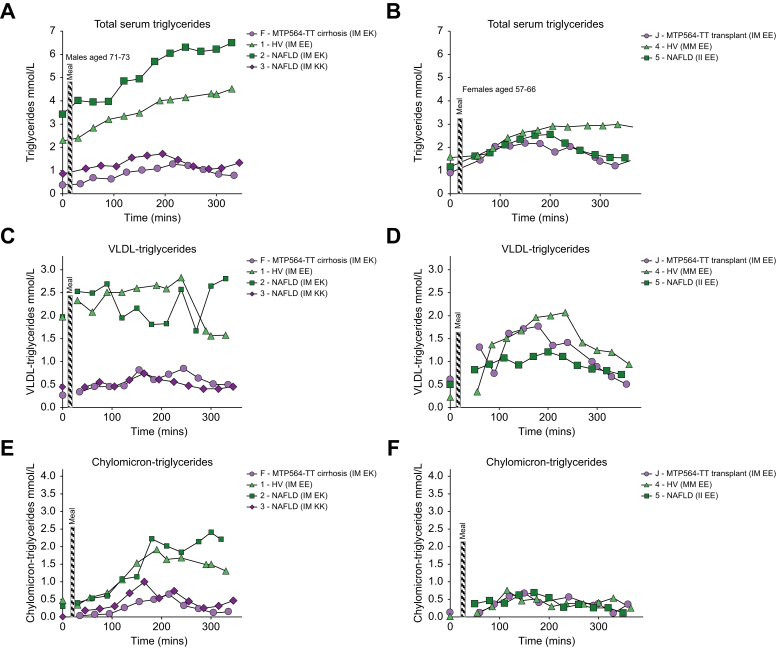
Triglyceride levels in study participants. Total serum triglycerides: (A) Participant F and matched controls and (B) participant J and matched controls. VLDL-triglyceride: (C) Participant F and matched controls and (D) participant J and matched controls. Chylomicron-triglyceride: (E) Participant F and matched controls and (F) participant J and matched controls. *MTTP* genotypes are shown for family members. *PNPLA3* p.I148M and *TM6SF2* p.E167 K genotypes are indicated in parentheses. HV, healthy volunteers; NAFLD, non-alcoholic fatty liver disease.

The levels of circulating triglyceride in participant F were lower than those in the healthy and disease controls (participants 1 and 2; Fig. 3A) but were similar to levels in participant 3, who possessed two variant alleles for *TM6SF2* rs58542926 (TM6SF2-KK). TM6SF2 is also involved in VLDL secretion and the variant associated with impaired function and decreased serum LDL-cholesterol.^20^^,^^21^ In contrast, the MTP564-TT homozygote with a liver transplant showed a similar triglyceride response to her two matched controls (Fig. 3B). Investigation of VLDL- and chylomicron lipoprotein-associated triglycerides (Fig. 3C–F) revealed that both components were again lowered in participants F (MTP564-TT) and 3 (TM6SF2-KK) compared with the controls, whereas they appeared normal in the MTP564-TT with a transplant, J. Other family members had no notable defects in lipoprotein triglycerides (Fig. S2). Furthermore, VLDL-cholesterol levels were similarly blunted in participants F (MTP564-TT) and 3 (TM6SF2-KK) but not in other family members (Fig. S3), suggesting that transplant hepatocytes and MTP564-IT heterozygote hepatocytes are functioning effectively in VLDL secretion.

Of note, lipoprotein-associated lipid levels are also lower in participant 7, a patient with NAFLD who is homozygous for the *PNPLA3* rs738409 variant (PNPLA3-MM), which has been linked to a relative reduction in large VLDL secretion^22^ (Figs. S2A and S3F). Circulating free fatty acids, glucose, and insulin levels in the family members were unremarkable (Fig. S4).

### Generation of wild-type and *MTTP*^*(VAR/VAR)*^ hiPSCs for disease modelling

To elucidate the mechanisms driving hepatic steatosis in homozygous MTP564-TT patients, we generated hiPSCs and differentiated them into hepatocytes to create an *in vitro* model of the variant. Fibroblasts derived from participant 1, genotyped as MTP564-II (also PNPLA3-148-IM; TM6SF2-167-EE), and family member J, MTP564-TT (also PNPLA3-148-IM; TM6SF2-167-EE), were expanded up to passage 4 and then reprogrammed into hiPSCs: *MTTP*^*(WT/WT)*^ and *MTTP*^*(VAR/VAR)*^, respectively (Figs. S5–S7). Neither carry the TM6SF2 variant that could confound the observed phenotype. Reprogrammed fibroblasts displayed the typical features of hiPSCs forming dense cell colonies, with well-defined colony boarders, containing cells with a high nuclear-to-cytoplasm ratio. To confirm their pluripotent status, Oct3/4 and NANOG expression was assessed and their ability to differentiate into endoderm, mesoderm, and ectoderm determined. We confirmed the karyotype as normal, without any major chromosomal abnormalities.

### MTP levels are lower and lipoprotein secretion is impaired in *MTTP*^(*VAR/VAR*)^ HLCs compared with *MTTP*^(*WT/WT*)^ HLCs

**Fig. 4:**
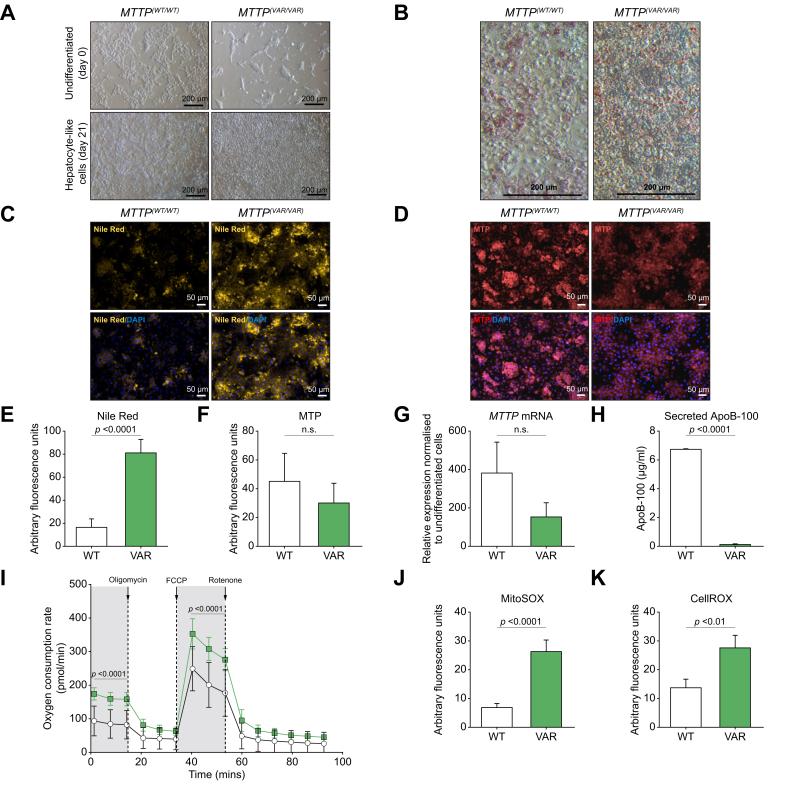
Characterisation of MTP-564T homozygote variant, *MTTP*^(*VAR/VAR*)^, and wild-type HLCs. (A) Light microscopy image of terminally differentiated hiPSC-derived HLCs. (B) Oil Red O staining of HLCs (light microscopy). (C) Nile red staining of lipids ± DAPI staining (fluorescence microscopy). (D) MTP expression immunocytochemistry ± DAPI staining. Quantification of Nile red fluorescence (E) and MTP staining (F) in HLCs. (G) Expression of MTP determined by quantitaive PCR. (H) ApoB-100 secretion by HLCs (ELISA). (I) Basal and maximal mitochondrial respiratory rates in *MTTP*^*(WT/WT)*^ (white circles) and *MTTP*^*(VAR/VAR)*^ (green squares) HLCs. Quantification of cellular superoxide (J) and reactive oxygen species (K) from fluorescence microscopy. Mean ± SE. *p* <0.05 is significant (*t* test, paired, two-tailed). ApoB-100, apolipoprotein B-100; hiPSC, human induced pluripotent stem cell; HLC, hepatocyte-like cell; MTP, microsomal triglyceride transfer protein.

To prevent bias as a result of differing differentiation efficiency of hiPSC lines, we differentiated cells from study donors 1 and J into HLCs to compare morphology and gene expression profiles.^12^ Both cell lines appeared morphologically similar during all stages of differentiation and generated a monolayer of HLCs by Day 21 (Fig. 4A). Gene expression was similar at each of the developmental time points including definitive endoderm, foregut endoderm, and hepatoblast cells. Expression of genes associated with a mature hepatocyte phenotype was not significantly different between the two cell lines (Fig. S7). Analysis of mRNA expression patterns for both cell lines primarily matched ‘liver bulk tissue’ and then ‘hepatocyte’ (Table S2), and both showed high similarity to HepG2, HuH7, and Hep3B cell lines.

Nile red and Oil Red O staining revealed phenotypical differences with apparent significant sequestration of lipid via development of discrete lipid droplets and microvesicular steatosis throughout the cytoplasm in the *MTTP*^*(VAR/VAR)*^ HLCs after 48 h of culture (Fig. 4B and C). Quantification showed levels were more than fourfold higher in *MTTP*^*(VAR/VAR)*^ HLCs (Fig. 4E), consistent with the proposed reduced VLDL secretion in cells expressing MTP564-TT, restricting removal of intracellular triglycerides.

Importantly, however, although both immunocytochemistry and mRNA expression analysis suggested a trend towards lower MTP levels compared with cells expressing the wild-type allele, this was not statistically significant (Fig. 4D, F, and G). To assess the VLDL export capabilities of the cell lines, and thus functioning of variant MTP in lipoprotein biosynthesis, levels of secreted ApoB-100 were determined. There was significantly less, but detectable, ApoB-100 in the media from *MTTP*^*(VAR/VAR)*^ compared with *MTTP*^*(WT/WT)*^ HLCs (Fig. 4H), confirming the clinical phenotype and supporting the suggestion that the MTP variant in these patients affects lipid trafficking.

### Increased generation of ROS and altered mitochondrial respiration in *MTTP*^(*VAR/VAR*)^ HLCs

Hepatic free fatty acids can be converted to triglyceride for storage as cytoplasmic droplets or secreted as VLDL, or else directly metabolised via mitochondrial β-oxidation. Therefore, impaired MTP functionality restricting lipid secretion, thus increasing the availability of fatty acids, may impact on mitochondrial activities. Using mitochondrial stress testing measuring the oxygen consumption rate in live cells revealed that *MTTP*^*(VAR/VAR)*^ HLCs had significantly higher basal and maximal mitochondrial respiration than mitochondria from the wild-type cell line (Fig. 4I). Of importance, increased β-oxidation would generate additional ROS, which can be a major driver of oxidative stress and cellular dysfunction. Both mitochondrial superoxide production and cytoplasmic ROS were significantly higher in the *MTTP*^*(VAR/VAR)*^ HLCs than in *MTTP*^*(WT/WT)*^ HLCs (Fig. 4J and K and Fig. S8), consistent with increased fatty acid metabolism.

### Increased NF-κB signalling, inflammation, ER stress, and secretion of pro-inflammatory mediators in *MTTP*^(*VAR/VAR*)^ HLCs

**Fig. 5:**
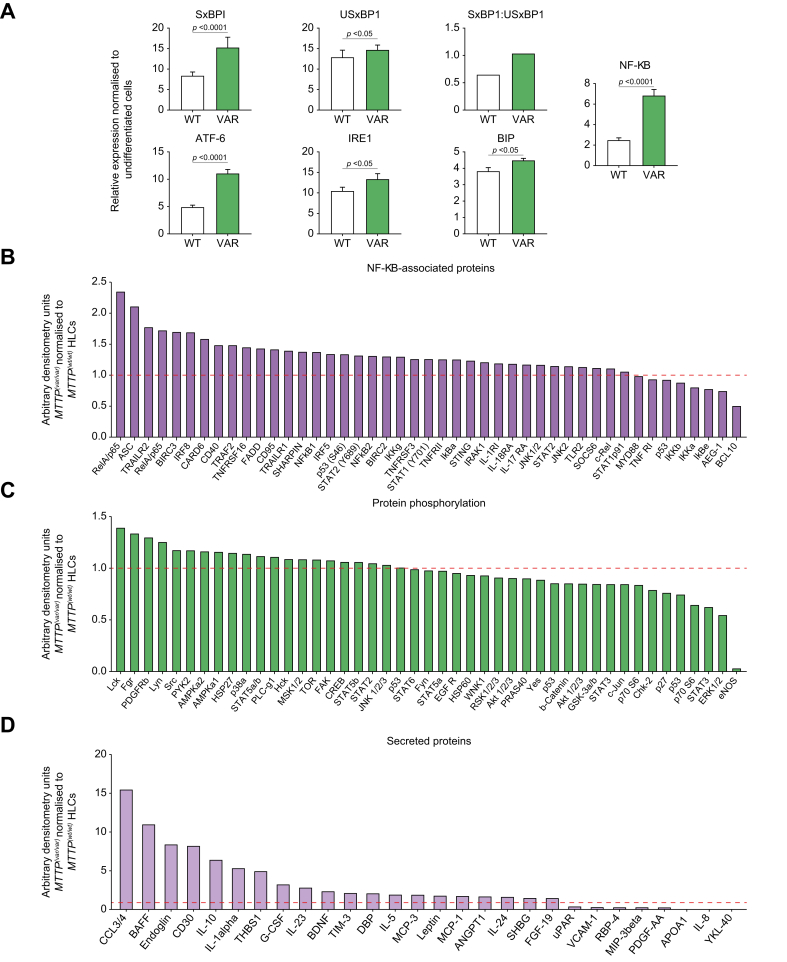
Phenotypic characterisation of *MTTP*^*(VAR/VAR)*^ hiPSC-derived HLCs. (A) Expression of inflammation-related and ER stress-related genes. Mean ± SE. *p* <0.05 is significant (*t* test, paired, two-tailed) (B) Expression of NF-κB-associated intracellular signalling proteins in *MTTP*^*(VAR/VAR)*^ HLCs, relative to expression in *MTTP*^*(WT/WT)*^. (C) Phosphorylated proteins in *MTTP*^*(VAR/VAR)*^ HLCs, normalised to *MTTP*^*(WT/WT)*^. (D) Secreted proteins from *MTTP*^*(VAR/VAR)*^ HLCs, normalised to *MTTP*^*(WT/WT)*^. ATF6, activating transcription factor 6; BIP, binding immunoglobulin protein; ER, endoplasmic reticulum; hiPSC, human induced pluripotent stem cell; HLC, hepatocyte-like cell; IRE1, inositol requiring enzyme 1; SxBP1, spliced X-box binding protein-1; USxBP1, unspliced X-box binding protein-1.

Impaired lipid trafficking and lipoprotein assembly incurred as a consequence of reduced MTP functionality is likely to cause a range of cellular responses including endoplasmic reticulum (ER) stress and inflammation. Analysis of mRNA revealed that expression of ER stress mediators spliced X-box binding protein-1 (SxBP1), activating transcription factor 6 (ATF6), and binding immunoglobulin protein (BIP), and the ER stress transducer inositol requiring enzyme 1 (IRE1) were significantly higher in *MTTP*^*(VAR/VAR)*^ HLCs (Fig. 5A).

*MTTP*^*(VAR/VAR)*^ HLCs expressed significantly higher levels of NF-κB (Fig. 5B), suggesting greater ER stress and active pro-inflammatory response.

These observations were confirmed by assessing changes in intracellular and extracellular signalling (Fig. 5C–E). The observed lipid accumulation in the novel MTP564-TT variant strain was associated with increases in NF-κB pathway components, indicative of activation, and phosphorylation of pro-inflammatory and pro-apoptotic pathway mediators including RelA/p65 complex, apoptosis-associated speck-like protein containing a caspase-recruitment domain (ASC), p53, Fas-associated death domain (FADD), and CD95. This increased pro-inflammatory signalling coincided with increased secretion of pro-inflammatory mediators including chemokine (C-C motif) ligand 3/4 (CCL3/4), B cell–activating factor of the TNF family (BAFF), CD30, IL-1, IL-10, IL-23, D-box binding PAR BZIP transcription factor (DBP), and leptin while showing decreased retinol binding protein 4 (RBP4) and platelet-derived growth factor (PDGF). Notably, *MTTP*^*(VAR/VAR)*^ HLCs had more than 10-fold lower expression of phosphorylated endothelial nitric oxide synthase (eNOS) than *MTTP*^*(WT/WT)*^ HLCs. There were no significant differences in protein phosphorylation in the other 44 proteins assayed. However, there was a trend towards decreased extracellular signal-regulated kinase 1/2 (ERK1/2) phosphorylation in *MTTP*^*(VAR/VAR)*^ HLCs.

### *MTTP*^(*VAR/VAR*)^ HLCs show increased expression of ECM remodelling and lipid metabolising genes

Bioinformatics analysis of mRNA-sequencing data to assess genome-wide changes in gene expression revealed 472 genes differentially expressed (>1 × log_2_-fold) between *MTTP*^*(VAR/VAR)*^ and *MTTP*^*(WT/WT)*^ cultured HLCs. These fulfil diverse cellular functions including glycolysis, lipid oxidation, oxidative phosphorylation, and complement activation (Fig. S9) and support previous observations of increased ROS generation and altered mitochondrial activity. Gene Ontology terms associated with the changing genes (Tables S3–S5) were mostly implicated in extracellular matrix (ECM) remodelling, ECM organisation and degradation, ECM–receptor interactions, and proteoglycan modifications, suggesting that ECM remodelling may be initiated during hepatosteatosis.

### Confirmation that the homozygous rs745447480 variant in the *MTTP*^(*VAR/VAR*)^ HLCs results in significantly lower MTP lipid transfer activity

**Fig. 6:**
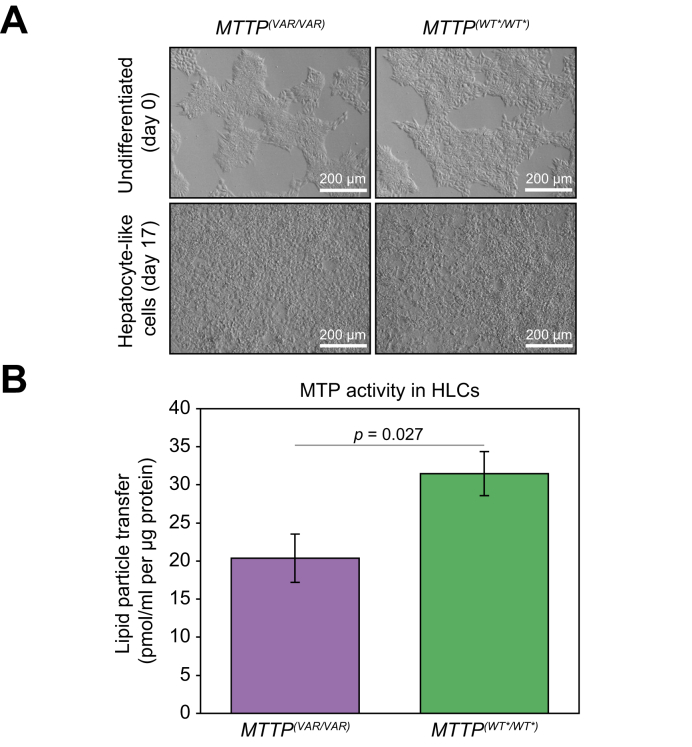
Restoration of activities by gene editing of *MTTP*^*VAR/VAR*^ 564-TT to 564-II. (A) Light microscopy showing terminally differentiated hiPSC-derived HLCs from *MTTP*^*(VAR/VAR)*^ and gene-edited derivative *MTTP*^*(WT*^*^∗^*^*/WT*^^∗^^*)*^*.* (B) MTP enzyme activity in HLCs. Mean ± SE; significance level *p* <0.05 (*t* test). hiPSC, human induced pluripotent stem cell; HLC, hepatocyte-like cell; MTP, microsomal triglyceride transfer protein.

A third cell line, *MTTP*^(*WT*^*^∗^*^*/WT*^*^∗^*^)^, was generated from *MTTP*^(*VAR/VAR*)^ in which the MTP564-TT in was gene-edited to wild-type 564-II using CRISPR-Cas9 transfection and selection of a corrected cloned (Fig. S10). The resultant differentiated cell line displayed the same characteristics as the *MTTP*^(*WT/WT*)^ cell line (Fig. 6 and Fig. S11). This enables us to rule out the possibility of other genetic variants harboured by the patient or healthy volunteer influencing the observed *in vitro* phenotype. MTP lipid transfer activity of the original patient-derived HLCs, *MTTP*^(*VAR/VAR*)^, was compared with that of the gene-edited HLCs to establish the impact of the SNP rs745447480. For equivalent cellular protein quantity, MTP activity was significantly lower in the *MTTP*^(*VAR/VAR*)^ HLCs than in the edited derivative *MTTP*^(*WT*^*^∗^*^*/WT*^*^∗^*^)^, having only 61% of the level determined in the wild-type cells. This is distinct from other described variants that abolish MTP activity and may explain the observed apparent phenotype of impaired lipid trafficking. To assure that the observed phenotype does not reflect compensatory activity of TM6SF2 in the same pathway, *TM6SF2* mRNA expression levels were determined. This indicated that TM6SF2 expression, normalised to undifferentiated cells, is not higher in *MTTP*^(*VAR/VAR*)^ than in *MTTP*^(*WT*^*^∗^*^*/WT*^*^∗^*^*)*^ and therefore suggests that the observed phenotype is not caused by increased TM6SF2 (Fig. S12).

## Discussion

We have identified and characterised a rare *MTTP* variant (p.I564T) as causative for the Mendelian trait associated with an inherited form of NAFLD in a four-generation family. Our investigation has revealed a variant resulting in decreased ApoB-containing lipoprotein secretion in homozygotes (but not heterozygotes), in contrast to other variants causing abetalipoproteinaemia, where ApoB is undetectable (Fig. 2 and Table 1).^6^^,^^7^^,^[23], [24], [25] Although other carriages of this variant have been reported, no phenotypic characteristics related to these are previously described.^15^ None of the GWAS so far, including that using UK Biobank^26^ and the largest cross-ancestry GWAS,^27^ have identified this particular *MTTP* variant (p.I564T) in association with NAFLD. Protein modelling (Fig. 1C) suggests that the substitution moderately affects the protein structure and likely impacts upon the interaction with PDI in the formation of a normal heterodimeric enzyme but is unlikely to abolish all functionality as in abetalipoproteinaemia. Presentation of homozygote cases is clearly distinct from abetalipoproteinaemia,^23^^,^^28^ supporting the suggested subtle phenotype whereby impact is limited to liver lipid imbalance. This provides potential for treatment through reduced dietary fat intake and makes it an attractive model for cellular consequences of lipid accumulation.

Our phenotyping studies demonstrated distinct VLDL secretion responses following meal challenge and ApoB levels in the two *MTTP*-564TT family members: although these biomarkers were substantially low in untreated individual F, these were in the normal range in liver transplant recipient J (Fig. 2B and Table 1). Previously, the *MTTP* -493 variant (rs1800591) G allele linked with reduced MTP function has been associated with NAFLD susceptibility in a meta-analysis of 11 case–control studies.^29^ Moreover, an association study in patients without diabetes with NASH found that GG homozygotes had significantly higher plasma triglycerides, intestinal and hepatic large VLDL, and oxidised LDL than the GG/GT group.^30^ All of the five MTP564-IT heterozygous individuals showed ApoB and lipoprotein levels within the normal range, consistent with reports that a single copy of *MTTP* is sufficient.^31^

Functional analysis of a childhood case with compound heterozygosity for *MTTP* c.619-5_619-2del and p.L435H, having severe liver fibrosis but no typical abetaliporoteinaemia symptoms, found that p.L435H abolished MTP activity, whereas the intronic variant resulted in 26% of transcripts being normally spliced, allowing limited MTP expression.^32^ This suggested that the residual expression and resulting MTP activity was sufficient for substantial biological activity covering majority of necessary functionality, except for liver functions. Consistent with this, our observed modest reduction in activity to 61% would thus be predicted to have a subtle effect only on liver homoeostasis. The modest impact on protein function *in vitro* is compatible with the less severe, non-abetalipoproteinaemia clinical phenotypes described. The observed ‘normal’ levels of expression in HLCs may suggest that the I564M change effects translation, protein stability/turnover, or enzymatic function. Our *in silico* models suggested that the impact is on heterodimer assembly, stability, interaction, or activity. An impact on activity would be entirely compatible with the unchanged protein levels observed in HLCs.

Disruption of ApoB biosynthesis and associated VLDL secretion has been widely described with a spectrum of consequences linked to characterised pathologies.^33^ ApoB missense variants are also associated with development of fibrosis and HCC linked to NAFLD.^34^ Furthermore, rare variants in *MTTP* were found to be associated with increased hepatic fat in the UK Biobank cohort.^5^ The underlying mechanisms are inherently linked to nutritional intake with diets high in fats (increasing hepatic fat content) and carbohydrate (increasing hepatic *de novo* lipogenesis), resulting in hyperlipidaemia. Hepatic lipid balance is dependent on secretion of VLDL, which is limited by availability/activity of MTP, so any variants with altered activities are likely to have metabolic effects. The low frequency of the *MTTP* p.I564T variant reported in large datasets^16^ means that identification, recruitment, and analysis of further carriers to strengthen the study would be very difficult.

Overall, VLDL secretion may increase with hepatic steatosis related to metabolic syndrome.^35^ However, decreased VLDL secretion has been reported in carriers of PNPLA3 G allele.^22^ VLDL secretion is also lowered in *TM6SF2* T carriers,^20^^,^^21^^,^^36^ which affects the same pathway. We considered key genetic risk factors, *PNPLA3* and *TM6SF2* variants, likely to influence disease phenotype as polygenic scores have been proposed for NAFLD.^37^ *TM6SF2* p.E167K is of particular interest because it acts in the same pathway as MTP, so similarities in phenotypes would be expected. In our study, post-prandial secretion of VLDL, the predominant post-prandial lipoprotein associated with hyperlipidaemia,^38^ was lower in participants homozygous for *TM6SF2* or *PNPLA3*, but fasting ApoB levels were normal. By contrast, in *MTTP* p.I564T homozygotes, we observed a reduced level of both circulating ApoB and VLDL-associated lipids. We have specifically considered the possibility of functional redundancy between TM6SF2 and MTP and potential additive effects of the variants *in vivo* and *in vitro*. First, the clinical characterisation in Table 1 shows that the disease phenotype is not linked to the *TM6SF2* carriage as three affected siblings do not carry the *TM6SF2* variant. Second, the post-prandial lipid data are consistent with reduction in circulating lipids owing to the lack of either wild-type *MTTP* or wild-type *TM6SF2*, whereas heterozygotes retain functionality.

In addition to demonstrating the functional consequences of the *MTTP* p.I564T variant, the HLCs derived from hiPSCs provide a disease model for early-stage NAFLD, beyond triglyceride accumulation. As the donors are wild type for *TM6SF2*, the cell phenotype reflects only the impact of the *MTTP* variant. Studies have shown a link between the amount of steatosis, fibrosis development, and liver disease mortality,^39^ with lipid metabolism acting as the initiator of progression to NASH.^40^ Although triglyceride sequestration may be protective, when fatty acid storage and disposal routes reach capacity, alternative pathways resulting in lipotoxicity can occur. Components of these pathways, such as acetyl-CoA carboxylase-1/2 (ACC-1/2), farnesoid X-activated receptor (FXR), fibroblast growth factor-19 (FGF19), and stearoyl-Coenzyme A desaturase-1 (SCD-1), are thus being tested as therapeutic targets.^41^ Increased mitochondrial fatty acid β-oxidation may provide a protective response but uncontrolled results in the generation of ROS, which can be a major driver of oxidative stress and cellular dysfunction (Fig. 4, Fig. 6).

We show that as lipid accumulation increases, hepatocytes have increased ER stress; activate pro-inflammatory signalling pathways including NF-κB, P53, and eNOS; and secrete pro-inflammatory mediators. This coincides with increased production of ROS, superoxide production, and alterations to mitochondrial respiration driving the disease progression leading to cirrhosis and HCC, as seen among the family members. Similar findings were reported in cardiomyocytes derived in an *MTTP* pR46G variant model.^42^ Excessive lipid accumulation in hepatocytes can serve as substrates for the generation of lipotoxic species. One of the major consequences of hepatic lipid metabolism is mitochondrial β-oxidation and esterification to form triglycerides, which can serve as a protective mechanism against lipotoxicity in hepatocytes. However, if lipid accumulation is in excess of the β-oxidation capacity, such as in NAFLD, toxic intermediates can accumulate, which induce metabolic stress and subsequent inflammation and cell death. Changes in expression of ECM remodelling-associated genes, suggestive of ECM remodelling occuring during steatosis, may contribute to drive progression to fibrosis, which is clinically observed later.

We conclude that the main feature of the *MTTP* p.I564T variant is impaired ApoB secretion and hepatic lipid accumulation as a result of decreased lipid transfer activity distinct from the classical abetalipoproteinaemia phenotype where MTP expression is abolished. Identification and characterisation of a rare disease such as hereditary NAFLD is of medical significance in Indian populations where high rates of founder events have been reported.^43^ In addition, HLC modelling supports this, providing additional details of signalling, inflammatory, and metabolic cellular pathways involved, highlighting pathophysiology driving NAFLD progression and possible therapeutic targets.

## Financial support

This work was supported by the Medical Research Council (MRC) Nottingham Molecular Pathology Node (grant number MR/N005953/1), National Institute of Health Research (NIHR) Nottingham Digestive Diseases Biomedical Research Unit, and Nottingham 10.13039/100014653Biomedical Research Centre (BRC-1215-20003). JIG and GPA are supported by NIHR Nottingham Biomedical Research Centre. KTS and LKB are supported by Population Health and Research Institute. All cell modelling was supported by the RoseTrees Trust and the Stoneygate Trust (M546). NRFH and SCO are supported by the Medical Research Council (MR/S009930/1). LVW holds a GSK/British Lung Foundation Chair in Respiratory Research (C17-1). The research was supported by the NIHR Leicester Biomedical Research Centre; CJ held a 10.13039/501100000265Medical Research Council Clinical Research Training Fellowship (MR/P00167X/1). EXCEED is supported by the 10.13039/501100000738University of Leicester, the NIHR Leicester Respiratory Biomedical Research Centre; by Wellcome (202849); and by Cohort Access fees from studies funded by the Medical Research Council (MRC), BBRSC, NIHR, the UK Space Agency, and GSK. This work is supported by BREATHE – The Health Data Research Hub for Respiratory Health (UKR_PC_19004) in partnership with SAIL Databank. The exome sequencing was funded by MRC Grant Senior Clinical Fellowship to MDT (G0902313), and we thank the high-throughput genomics group at the Wellcome Trust Centre for Human Genetics (funded by Wellcome Trust Grant 090532/Z/09/Z and MRC Hub Grant G090074791070) for the generation of the sequence data. MDT is supported by a Wellcome Trust Investigator Award (WT202849/Z/16/Z) and holds an NIHR Senior Investigator Award. The funders had no role in study design, data collection and analysis, decision to publish, or preparation of the manuscript.

## Authors’ contributions

Conceptualisation: GPA . Study design: GPA, JB, NRFH, AMS, LVW, MDT. Data curation: JIG, PCKL, NRFH, EJH, NS, NB, CJ, IN. Funding: GPA, NRFH, EJH, KTS, MDT, LVW. Formal analysis: JIG, NRFH, PCKL, NS, NB. Investigation: JIG, PCKL, GPA, NRFH, JB, NS, ANB, SCO, NB, EJH, AG, GEJ, MGT, HK, ABA, PG, VMV. Supervision: GPA, NRFH, JB, AMS, JIG, LVW, MDT . Resources: GPA, JIG, NRFH, AMS, JB, CJ, IN, CPN, KTS, LKB. Interpretation of data: GPA, JIG, NRFH, AMS, MDT, JB, LVW, EJH. Writing – original draft: JIG, PCKL, NRFH, AMS, JB, LVW, GPA . Writing – review and editing: all authors.

## Data availability statement

Study data are available on request. The three EXCEED exome sequences are available in the European Genome-phenome Archive using accession number EGAD00001007649. Access to sensitive genetic data and cell lines will be restricted to research facilities with institutional data and material transfer agreements to protect participant anonymity.

## Conflicts of interest

GPA has served as a consultant and an advisory board member for Pfizer Inc, Inventiva Pharma, GlaxoSmithKline, and KaNDy Therapeutics; he has been a consultant to Servier, Clinipace, Albireo Pharma, BenevolentAI Bio, DNDi, BerGenBio ASA, Median Technologies, FRACTYL, Amryt Pharma, and AstraZeneca; and has given presentations on behalf of Roche Diagnostics and Medscape. IN is employed by Gilead Sciences Ltd. (since August 2019). All other authors declare no conflict of interests.

Please refer to the accompanying ICMJE disclosure forms for further details.
