# Supplementary material for: Identification and characterisation of a rare MTTP variant underlying hereditary non-alcoholic fatty liver disease
Source: JHEP Rep. 2023 Apr 23;5(8):100764. doi: 10.1016/j.jhepr.2023.100764 (PMC10362796; doi:10.1016/j.jhepr.2023.100764)
Supplement: Multimedia component 1 [file mmc1.pdf]

Supplementary materials

Identification and characterisation of a rare *MTTP* variant underlying hereditary non-alcoholic fatty liver disease.

Jane I. Grove, Peggy Cho Kiu Lo, Nick Shrine , Julian Barwell, Louise V. Wain, Martin D. Tobin , Andrew M. Salter, Aditi Borkar, Sara Cuevas-Ocana, Neil Bennett, Catherine John, Ioanna Ntalla, Gabriela E. Jones, Christopher P. Neal, Mervyn G. Thomas, Helen Kuht , Pankaj Gupta, Vishwaraj M. Vemala, Allister Grant, Adeolu B. Adewoye, Kotacherry T. Shenoy , Leena K. Balakumaran, Edward J. Hollox, Nicholas R.F. Hannan, Guruprasad P. Aithal

Table of Contents:

Supplemental Methods……………………………………………………………………2

Supplemental Figures…………………………………………………………….......13

Supplemental Tables…………………………………………………………………28

Supplemental References…………………………………………………………....34

Supplemental Methods

See CTAT table for addition details of materials

Patient Investigations

Family members were screened at a joint hepatology-genetics clinic. Liver ultrasound and fibroscan® were used to detect evidence of steatosis and if seen in the context of abnormal liver function tests, NAFLD was diagnosed. Details of clinical investigations: Eyes: any evidence of night blindness, progressive reduced vision, retinitis pigmentosa, nystagmus and ophthalmoplegia; Neurological: ataxia (if any what age of onset), slurred speech, muscle weakness, loss of reflexes; Skeletal: lordosis, kyphoscoliosis, high arched feet (pes cavus, club foot); Gastrointestinal: fat malabsorption, greasy pale stools, steatorrhea; Developmental: failure to thrive, intellectual disability, developmental delay; blood tests: peripheral smear for acanthocytosis, low number of red blood cells; lipid profile: hypocholesterolaemia; duodenal biopsy; liver profiles, ultrasound and fibroscan®, or biopsy.

Meal Response Study

Foods: Breakfast was 70g Kelloggs Cornflakes, 300ml Tesco British Whole Milk with 35g Nestle Lido full cream milk powder (total: 633kcals; 21.3g fat; 84.9g carbohydrate; 23.5g protein; 2.1g fibre; 1.5g salt). Lunch was Sainsbury's Indian Vegetable Biryani (500g) meal and milkshake containing 200ml Tesco whole milk, 20ml Tesco fresh double cream and Kelly's clotted cream vanilla ice cream (total: 1134kcals; 58.7g fat; 119.9g carbohydrate; 23.9g protein; 15.7g fibre; 1.77g salt).

Blood Sampling

Blood samples were collected in vacutainer tubes containing: 5mg sodium fluoride and 4mg potassium oxalate (for glucose analysis), lithium heparin, potassium EDTA and stored on ice, or in tubes without additive (all Becton Dickinson) for serum after coagulation at room temperature for 30 min. For free fatty acid analysis 45µl of glutathione/ethylene glycol tetra acetic acid was added

per 6ml blood in with lithium heparin (Sigma-Aldrich) and 15µl Tetrahydrolipostatin was also added to inhibit lipase activity[1]. Plasma free fatty acids were measured using Wako NEFA C enzymatic colour test method (Wako Chemicals GmbH). Caspase-cleaved CK-18 was quantified in duplicate using M30 Apoptosense ELISA (Peviva, Sweden). Blood was centrifuged at 2000g for 10 min either at room temperature for serum, or at 4°C for plasma and the upper layer transferred to cryovials. EDTA blood was collected for DNA extraction. Whole blood, plasma and serum samples were stored at -80°C prior to metabolic analyses, or used immediately.

Lipoprotein Preparation

Lipoprotein fractions were prepared from plasma within 12h of blood sampling.
Plasma lipoproteins were separated by sequential non-equilibrium density-gradient ultracentrifugation by established techniques based on those originally described [2]. Up to 3 ml EDTA plasma was pipetted into quick-seal ultracentrifuge tubes (Beckman Coulter, Inc., High Wycombe, UK) and were topped up with 1.006 g/ml potassium bromide (KBr) solution and sealed. The tubes were centrifuged in a Beckman Optima ultracentrifuge XL-70 under vacuum at 12°C using rotor 50.4 for 20 min at 12,000 rpm with full acceleration and no break. Afterwards each tube was cut open using a tube slicer (Kontron, Watford, UK). The top chylomicron layer was taken and made up to 2 ml with 1.006 g/ml KBr solution and stored at -20°C The lower lipoprotein layer was then transferred to fresh ultracentrifuge tubes and centrifuged under vacuum at 12°C using rotor 50.4 for 16 hrs at 39,000 rpm with full acceleration and no break. Following ultracentrifugation the top VLDL layer was removed, this and the lower layer were separately stored at -20°C.
Quantitative determination of TAG and cholesterol was carried out on the plasma, chylomicron and VLDL fractions using Infinity™ TAG and Cholesterol Liquid Stable Reagent kits (Thermo Fisher Scientific, UK), according to the manufacturer's instructions.

Variant identification

DNA was derived from blood except the 3 EXCEED study samples[3] which were derived from saliva. Whole exome sequencing (single batch with 3 replicates) mean depth of coverage was 42-66×. Exome enrichment was done in 3 batches using NimbleGen SeqCap EZ Exome v3.0 (64Mb). Samples were sequenced using 100bp paired-end sequencing on the Illumina HiSeq2000 (each sample sequenced in 2 lanes) and validated by Sanger sequencing. Both NAFLD cases and controls were present in each pair of lanes and in each batch to minimise confounding.

After alignment[4], reads were cleaned with Picard v1.93 (synchronise mate-pair information) and SAMtools[5] (PCR duplicates removed, sorted and indexed); local realignment around indels and recalibration of quality scores was done with GATK v3.2-2[6]. Variant calling was done using both GATK v3.2-2 HaplotypeCaller and SAMtools v1.1 mpileup to obtain a consensus. Concordance between the two methods was 96.8%. Concordance between the 3 pairs of replicate samples was 98.4-98.6%.

First we identified variants that were not called in any of the 9 control samples. This identified 8,835 SNPs unique to one or more NAFLD cases. We next excluded any SNPs that were also present in the following datasets: 1000 genomes phase I high confidence SNPs[7], 1000 Genomes Project (May 2013 release), 1000 genomes Illumina OMNI 2.5 SNP array[8], dbSNP version 138 and 142[9] (https://www.ncbi.nlm.nih.gov/snp/) HapMap3[10] (https://www.sanger.ac.uk/resources/downloads/human/hapmap3.html), NHLBI exomes[11] (March 2015; https://evs.gs.washington.edu/EVS/) and whole exome sequencing of 125 South Asian samples[12]. We restricted the remaining 1024 SNPs to those annotated as 'exonic' using ANNOVAR[13] (November 2014 version) which left 434. Of those, Variant Effect Predictor[14] annotated 159 SNPs as 'deleterious' by SIFT[15], 'probably damaging' or 'potentially damaging' by PolyPhen-2[16], or had a CADD[17] Phred scaled score≥20 or GWAVA[18] score >0.5. Segregation with disease was assessed by overlaying the variant genotypes on the pedigree (Fig. 1A).

A missense variant in *MTTP* seen in all 12 affected individuals (6 heterozygotes and 6 homozygotes) was the only variant that fully segregated with disease. Two additional variants were found as heterozygotes: N1484S in *NOTCH1* NC_000009.11:g.139399897T>C (10 cases) and G796R in *EPB41L1* NC_000020.10:g.34807716G>C (11 cases). The sequence was analysed via Phyre2.0[19] and SuSPect[20] webservers to study the effects of mutations on the protein structure, stability and possible function.

Genotyping

Genomic DNA was prepared using Flexigene DNA kit (Qiagen). PCR-RFLP genotyping used primers *MTTP*-F1 and *MTTP*-R1 followed by restriction digestion with Hpy166II and analysis by agarose gel electrophoresis. PCR-RFLP was used for genotyping rs738409 (FokI), rs58542926 (MspI) and rs58542926 (Hpy188I)[21]. For analysis of *MTTP* alleles, following removal of PCR components, the sequences of PCR products generated were determined using Sanger sequencing (Source Bioscience, Nottingham, UK). The following primer pairs were used to amplify DNA for genotyping common genetic variants: rs745447480: TCTTAACGGCCTCAGCCTAG & CAGAGTTACCAGTCATGGACTC; rs2306986 and rs3816873: AAGGTAGAATAGGGCAGGGGTCC & CTAATCTCAGTTGGATCATTTCAGTCTC; rs3792683: GTTACAGGTAGAGAACATGCTGACATG & CCTCCATGGTACAGTGGTGCAC; rs2306985: CAGTCACAGAGTCCTACCCAGG & GAGACTGCTGTCATCACAACTCTGTG; rs738409: CAGCTGTGGCTACTCTGTCTG & TGGAGAAAGCTTATGAAGGATCAG; rs58542926: CCAAAATGTTGGGATTACAGG & ACAGATGTCCAGCAGGGTTC.

Isolation and culture of human dermal fibroblasts

Primary human dermal fibroblasts were established via explant culture in DMEM medium supplemented with 2% Antibiotic-Antimycotic; 10% FBS; 1% GlutaMAX; 1% NEAA and 1% penicillin/streptomycin. Fibroblast medium was refreshed every 2-3 days, cells were split at 1:3-1:6

using 0.25% Trypsin-EDTA for 3min at 37°C when 80% confluent. All reagents were from Gibco (ThermoFisher, UK).

Fibroblast reprogramming and hiPSC maintenance

Skin biopsies from participant 1, genotyped as MTP564-II, and family member J, MTP564-TT, were dissected to remove subcutaneous fat and cultured in fibroblast media for approximately 10 days until fibroblasts began emerging from the biopsy forming a monolayer. Approximately 20,000 fibroblasts were seeded in a well of 6-well plate, cells were transduced 24h later at a MOI of 5:5:3 (hKOS: hc-Myc: hKlf4). Transduced cells were maintained in fibroblast medium for 3-7 days until they were 80-90% confluent, then transferred to a new 6-well plate at a split ratio of 1:3 using 0.05% Trypsin (Gibco) and switched to TeSR-E7 medium (StemCell Technologies, Cambridge, UK). When hiPSC colonies appeared around 13-22 days post-transduction, medium was replaced to TeSR-E8 (StemCell Technologies). Colonies were selected between day 13-45 post-transduction using either 0.5mM EDTA (Invitrogen), or ReLeSR (StemCell Technologies). Once the hiPSC lines were established, culture medium was transitioned from TeSR-E8 to an E8 medium (prepared on site) following the formula described previously (50), with an additional 100 ng/ml heparin sodium salt. HiPSCs were passaged every 3-4 days (75-90%) using TrypLE Express (Gibco) at 1:10-1:20 ratio and seeded onto matrigel- coated (Corning) Nunc plasticware (Thermofisher). E8 medium was supplemented with 10 μM Y27632 ROCK inhibitor (ROCKi; Tocris) for the initial 24h after splitting. The first undifferentiated colonies appeared 7 days post transduction, stable colonies were picked after 3 weeks and stable cell lines generated approximately 40 days post transduction. All cell lines tested negative for mycoplasma contamination using the EZ-PCR Mycoplasma Test Kit (Biological Industries) prior to reprogramming.

Differentiation to embryonic germ layers

For mesoderm differentiation hiPSCs were differentiated as described previously[22]. Briefly, hiPSCs were seeded at 20,000 cells/cm^2 on Matrigel-coated 48-well plate. 72 h later E8 medium

was replaced to mesoderm induction medium consisting of RPMI (Gibco), 213 μg/ml ascorbic acid and 500 μg/ml albumin (both from Sigma-Aldrich). 4 μM CHIR99021 was supplemented to the medium for the first two days. Cells were fixed with 4% paraformadehyde on day 4 for immunostaining.

For ectoderm differentiation hiPSCs were differentiated as described previously [23]. Briefly, hiPSCs were seeded onto Matrigel-coated 48-well plate at a density of 50,000 cells/cm^2. 24 h after seeding, E8 medium was replaced to RPMI medium supplemented with 2% B27; 1% NEAA; 10 μM SB431542 (Selleckchem) and 12 ng/ml FGF2 (Peprotech), medium was replaced daily.

Differentiation of hiPSCs into hepatocyte-like cells (HLCs)

All cells were differentiated into hepatocytes as described previously [24]. Briefly, hiPSCs were seeded at a density of 15,000-20,000 cells/cm^2 (dependant on the cell line) onto Matrigel-coated plasticware. Definitive endoderm differentiation was initiated 48h after seeding when cells were approximately 50% confluent, hiPSCs were cultured in RPMI with 2% B27 and 1% NEAA as basal medium, then supplemented with 100 ng/ml activin A and 50 ng/ml Wnt-3a (R&D) for 3 days. Foregut was generated by incubating cells in RPMI/B27/NEAA with 50 ng/ml activin A for an additional 2 days. Hepatic endoderm specification was achieved by supplementing RPMI/B27/NEAA with 10 μM SB431542, 10 ng/ml BMP4 (R&D Systems) and 20 ng/ml FGF10 for 4 days. Finally, medium was replaced every other day to HepatoZYME (Gibco) with 1% chemically defined-lipid concentrate (Gibco); 1% GlutaMAX; 1% NEAA; HGF; and oncostatin-M.

CRISPR-Cas9 mediated correction of I564T mutation in the MTTP$^{(VAR/VAR)}$

Nucleofection of the *MTTP*$^{(VAR/VAR)}$ parental line was optimised according to the manufacturer's recommendations (Lonza) using the provided eGFP plasmid and transfection efficiency was quantified by flow cytometry (Supplementary Fig. 10A). For CRISPR-Cas9 editing (Supplementary Fig. 10B-F), a single-guide RNA (5'-gaacatcctgctgtctactg) was cloned into the #51133 plasmid (Addgene) [25] and nucleofected into the *MTTP*$^{(VAR/VAR)}$ parental line along with #62988 (Addgene)

[26]. Plasmid # 51133 (pGL3-U6-sgRNA-PGK-puromycin) was a gift from Xingxu Huang [25] and #62988 (pSpCas9(BB)-2A-Puro (PX459) V2.0) was a gift from Feng Zhang [26]. Clones were manually isolated and screened for correction of the I564T mutation *MTTP*$^{(VAR/VAR)}$ to *MTTP*$^{(WT*/WT*)}$ (C>T) using PCR amplification of the target site (primers FWD and REV) followed by restriction digestion with Hpy166II (New England Biolabs) that specifically detect *MTTP*$^{(VAR/VAR)}$. Genetic modifications in the selected clones were confirmed by Sanger sequencing analysis (Source Bioscience Ltd, UK). Corrected *MTTP*$^{(WT*/WT*)}$ hiPSCs and the *MTTP*$^{(VAR/VAR)}$ parental hiPSC line were differentiated to HLCs in parallel for characterisation.

Karyotyping

30 metaphase spreads from exponentially growing hiPSC cultures were analysed by conventional karyotyping[27] (Nottingham University Hospitals NHS Trust).

Detection of cellular and mitochondrial ROS

Mitochondria content of HLCs were visualised using 100 nM MitoTracker green FM or MitoTracker deep red FM. Intracellular reactive oxygen species (ROS) and mitochondrial superoxide production were assessed using 2.5 μM CellROX green or 2.5 μM MitoSox Red respectively. Nuclei counterstaining was achieved using 5 μg/ml of Hoechst 33342 (Invitrogen). All reagents were purchased from Invitrogen and used according to manufacturer's guidance. Live cells were loaded with the dyes for 30 minutes at 37°C and 5% CO_2. Cells were then replaced with warm HepatoZYME (Gibco) media and imaged using the Operetta system (PerkinElmer). Images were captured at 445nm (Hoechst 33342), 525nm (CellROX green and MitoTracker green) and 705nm (MitoSOX Red and MitoTracker deep red). Fluorescence intensity was analysed using Columbus system (PerkinElmer) and plotted as mean ± SD from multiple image acquisition fields.

Mitochondrial respiration analysis

HLCs were dissociated using TrypLE Express (Gibco) and seeded onto XF96 plate with a density of 25,000-50,000 cells per well. HLCs were maintained for further 6 days and mitochondrial respiration analysed at D20. To assess mitochondrial respiration, culture medium was replaced with 200 µl Seahorse XF base medium supplemented with 10 mM glucose, 1 mM sodium pyruvate and 2 mM L-glutamine at 37°C without CO_2 for 1 h prior to measurements using the Seahorse XF96 analyser (Seahorse Bioscience, USA). Mitochondria stress tests were performed as recommended by the manufacturer, oxygen consumption rate (OCR) was measured while injecting oligomycin (1.5 µM), FCCP (0.4 µM), rotenone (1 µM) and XF base medium. OCR values were normalised by the number of viable cells counted with DAPI. All reagents were from Agilent.

RT-qPCR Gene expression analysis

Total RNA was isolated from HLCs cells using RNeasy Mini kit (Qiagen). Total RNA (500 µg) was reverse transcribed using the SuperScript II Reverse Transcriptase kit (Invitrogen) with random primers (Promega) and dNTP (Promega) according to the manufacturer's recommendations. Quantitative real-time PCR (qPCR) was performed as described previously [28, 29]: after cDNA samples diluted to a final volume of 600 µl with nuclease free water. qPCR reactions were run using cDNA samples (from initial 500 ng of reverse-transcribed RNA, diluted 1:30); 5 µM forward and reverse primers; with SensiMix SYBR & Fluorescein Kit (Bioline). qPCR amplification was performed on an Applied Biosystems 7500 Fast Real-Time PCR Systems using a three-step cycling programme of 40 cycles consisting incubation at 95°C (15 sec), 60°C (30 sec) and 72°C (30 sec). All samples were run with three technical replicates. Fold changes in expression of differentiating cells over undifferentiated hiPSCs were calculated using comparative ΔΔCt method standardised against the housekeeping gene PBGD, data were shown as mean of Ct values ± standard error of mean (SEM).

RNA sequencing and analysis

Total RNA was isolated from hiPSC-HLCs on day 21 using RNeasy Mini Kit (Qiagen). RNA yield was quantified by a Qubit Fluorometer (Invitrogen) with the Qubit RNA BR Assay Kit (ThermoFisher). RNA quality was assessed by Agilent RNA ScreenTape Assay kit all samples had an integrity number (RIN) ≥ 9.8. NEBNext rRNA Depletion Kit (New England Biolabs) was used to enrich mRNAs, depleted RNA samples were assessed using Agilent High Sensitivity RNA ScreenTape Assay Kit (RIN ranges 1.0-5.4). NEBNext Ultra II Directional RNA Library Prep Kit for Illumina (Set 1 and 2 indexes) was used to construct cDNA libraries. Concentration and sizes of the generated cDNAs were measured using Agilent High Sensitivity D1000 ScreenTape Assay Kit. 4200 TapeStation System (Agilent) was used to evaluate the quality of RNA and cDNA library. EnrichR was used to query the likely tissue and cell types based on gene expression [30]. Sequencing was done using the Illumina HiSeq 2500 system (high output mode) to yield targeted number of single-end 100bp reads to a depth of 30 million per sample. Reads were mapped to the GRCh38.p10 Ensembl human genome using Hisat2 (v2.1.0). Analysis was performed using SeqMonk (1.46.0) software, the read counts per gene was determined using RNA-Seq pipeline quantitation. Differential expression analysis was performed in R using DESeq2 (1.28.1) package. Data was trimmed with Trim Galore v0.6.2 using default parameters. It was aligned to the GRCh38 human genome assembly using Hisat2 v2.1.0 using the option "--sp 1000,1000" to prevent soft-clipping. The alignment was seeded with introns from gene models from Ensembl v87. Alignments with a MAPQ score of < 20 were discarded.

Per gene expression was quantitated against gene models from Ensembl v97 counting read overlaps to any exon of each gene. Only alignments on the opposite strand to the gene being measured were counted. For normalised expression visualisation log2 Reads per million reads of library (log2RPM) values were calculated, and these were then corrected using size factor normalisation based on genes which were measured in at least one replicate.

A similar process was employed for all differential expression calculations. An initial set of differentially expressed genes was calculated from raw counts using the DESeq2 package. Genes

with a FDR of <0.05 were retained. This list was further filtered using an expression normalised fold change z-score, again with a cut-off of FDR < 0.05. Final candidates were the intersection of the hits from these two tests.

Proteome profiler antibody arrays

Human NFκB Pathway, Phospho-Kinase and XL Cytokine Array Kits were all purchased from R&D Systems and used as specified by the manufacturer. Cell protein lysates were analysed using the NFκB and Phospho-kinase array kits; cell culture supernate from cultured HLCs collected 48 h after media change, was used for the XL Cytokine arrays.

Total protein concentration was quantified by NanoDrop-1000 spectrophotometer (Thermo-Fisher). Protein samples were incubated overnight and visualised the next day using ImageQuant LAS-4000 (Fujitsu Life Sciences). Image Studio Lite Software (version 5.2) was used to quantify the pixel intensity of each set of duplicated antibody spots and normalised by subtracting the averaged background signal. Comparison of protein expression was achieved by calculating the relative fold change with respect to signals produced by $MTTP^{(WT/WT)}$.

Immunocytochemistry

Cells were fixed in 4% paraformaldehyde (PFA; VWR Chemicals) for 20 min at 4°C, followed by 30 min blocking and membrane permeabilisation using 10% foetal bovine serum (FBS; Gibco) and 0.1% TritonX-100 (Thermo Scientific) in PBS (Gibco). Fixed cells were incubated overnight at 4°C in a combination of primary antibodies diluted in 1% FBS-PBST solution. The next day, cells were incubated with fluorescent-labelled secondary antibodies for 1 h at RT, followed by nuclei-counterstaining using 0.5 µg/ml DAPI (Sigma) in 1% FBS-PBST for 5 min. Antibodies used were MTTP (Abcam); OCT3/4 (Santa Cruz Biotechnology); NANOG (R&D Systems); MESP1 (Abcam); GATA-4 (R&D Systems); Nestin (Merck); SOX2 (Novus Biologicals); ALB (R&D Systems); CYP2A6 (OriGene Technologies); A1AT (Abcam); GST-pi (Enzo Life Sciences);

Secondary antibodies at 1/400 dilution: Donkey Anti-Mouse AF 488 (Invitrogen); Donkey Anti-Goat AF 647 (Invitrogen); Donkey Anti-Rabbit AF 647 (Invitrogen).

Microscopy

Cells were fixed and permeabilised and imaged on tissue culture plates containing PBS. All images were acquired using the Automated Operetta™ high content image analyser (PerkinElmer); captured images were then analysed using Columbus™ software (PerkinElmer), fluorescence intensity of specific channel was determined with the developed algorithms in the software. Fluorochromes were Alex Fluor 488 and 647. Images were captured at either 10x or 20x magnification as indicated. Expression was quantified in 10 fields in each. Mean fluorescence intensity in triplicate wells were compared using T-test.

Supplemental Figures

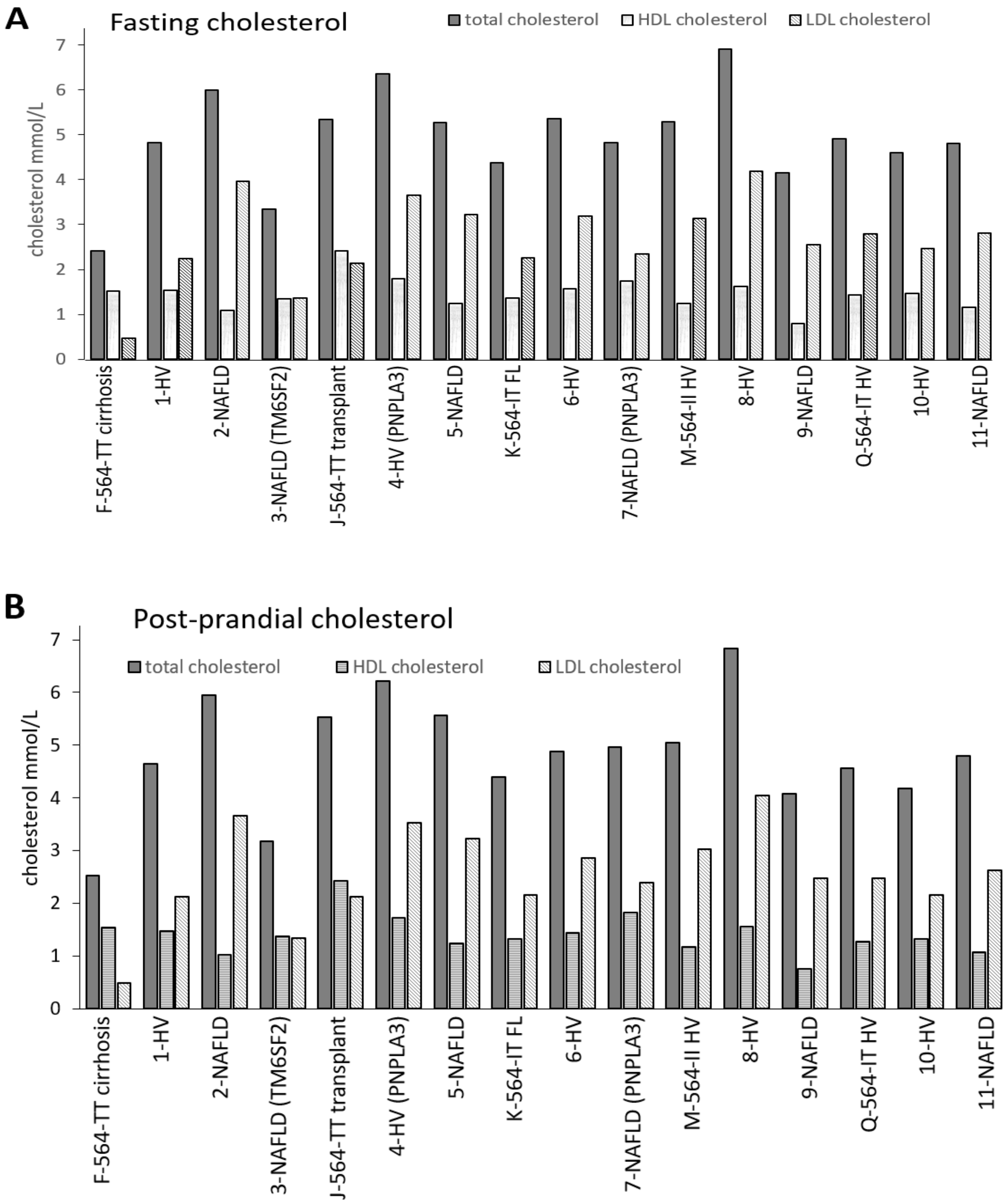

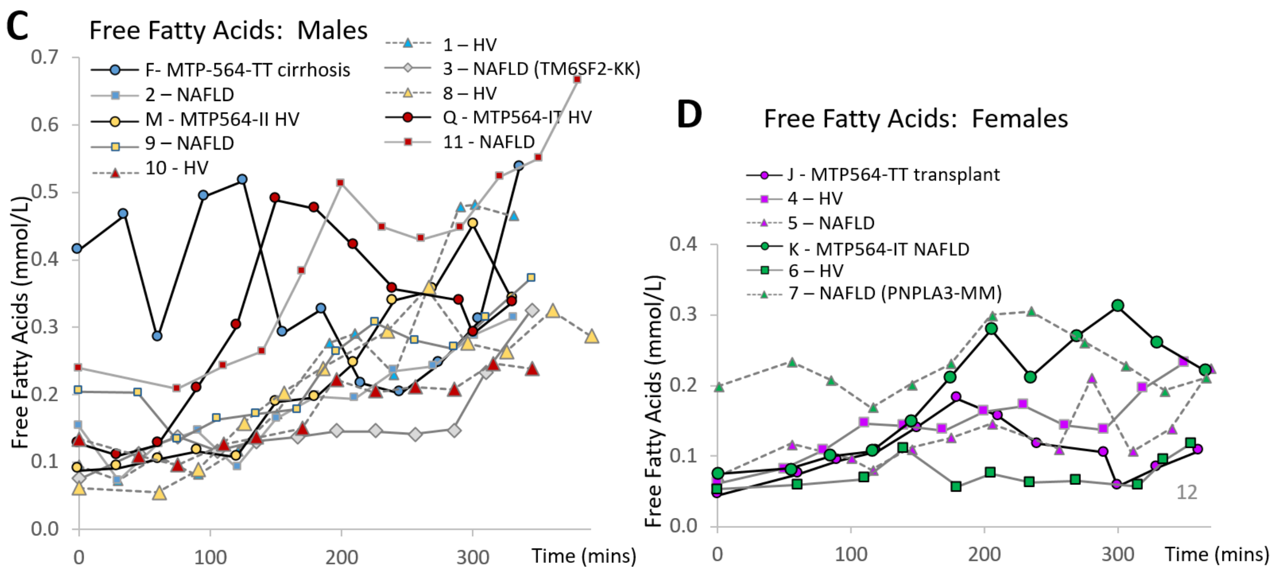

Figure S1. Serum cholesterol levels and free fatty acid levels in study participants.

Participants grouped according to age and gender matching with family member (Fig. 1a and Supplemental Table 3). Genes are shown in parentheses where participant is homozygous for other effect alleles: *PNPLA3* rs738409; *TM6SF2* rs58542926). (**A)** Fasting cholesterol. (**B)** Cholesterol level approx. 2h after eating standard study meal. (**C)** Free fatty acid levels in male participants. (**D)** Free fatty acid levels in female participants. NAFLD= non-alcoholic fatty liver disease; HV=healthy volunteer; FL=fatty liver. *PNPLA3* (rs738409) variant homozygotes (148-MM) and *TM6SF2* (rs58542926) variant homozygotes (167-KK) are indicated.

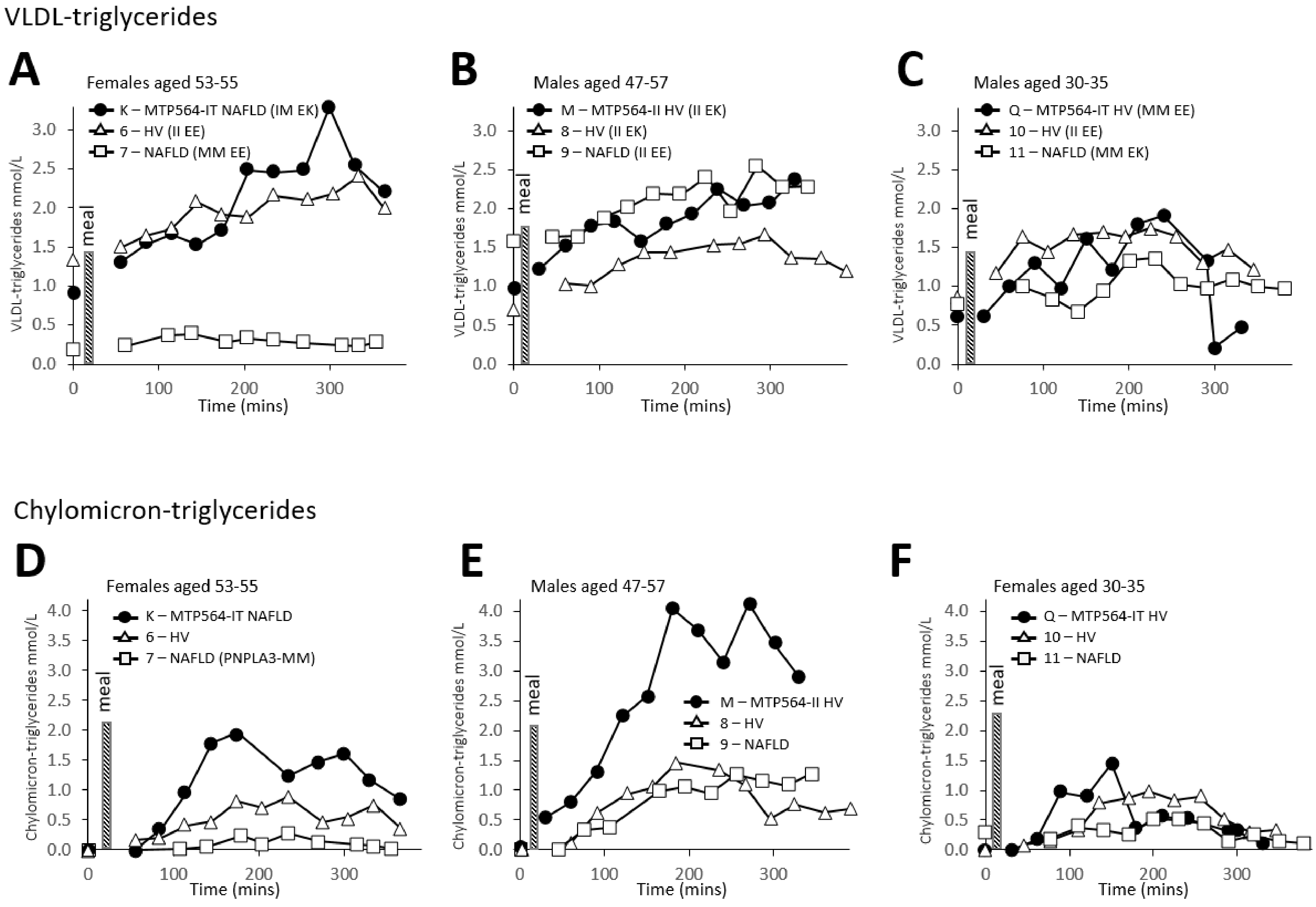

VLDL-triglycerides
A
Females aged 53-55
K – MTP564-IT NAFLD (IM EK)
6 – HV (II EE)
7 – NAFLD (MM EE)
B
Males aged 47-57
M – MTP564-II HV (II EK)
8 – HV (II EK)
9 – NAFLD (II EE)
C
Males aged 30-35
Q – MTP564-IT HV (MM EE)
10 – HV (II EE)
11 – NAFLD (MM EK)
VLDL-triglycerides mmol/L
meal
Time (mins)
Chylomicron-triglycerides
D
Females aged 53-55
K – MTP564-IT NAFLD
6 – HV
7 – NAFLD (PNPLA3-MM)
E
Males aged 47-57
M – MTP564-II HV
8 – HV
9 – NAFLD
F
Females aged 30-35
Q – MTP564-IT HV
10 – HV
11 – NAFLD
Chylomicron-triglycerides mmol/L
meal
Time (mins)

Figure S2. Lipoprotein-associated triglyceride levels in study participants before and after a fatty meal.

Participants are described in Supplemental Table 1**. (A)** Serum VLDL-triglycerides in MTP564-IT family member K and matched control participants: 6 (HV) and 7 (NAFLD patient). (**B)** VLDL-triglyceride in MTP564-II (wild type) family member M and matched control participants: 8 (HV) and 9 (NAFLD patient). (**C)** VLDL-triglycerides in MTP564-IT family member Q and matched control participants: 10 (HV) and 11 (NAFLD patient). (**D)** Chylomicron-triglycerides in participants K, 6 and 7. € Chylomicron-triglycerides in participants M, 8 and 9. (**F)** Chylomicron-triglycerides in participants Q, 10 and 11. *PNPLA3* p.I148M and *TM6SF2* pE167K genotypes are indicated in parentheses. HV=healthy volunteer; NAFLD=non-alcoholic fatty liver disease.

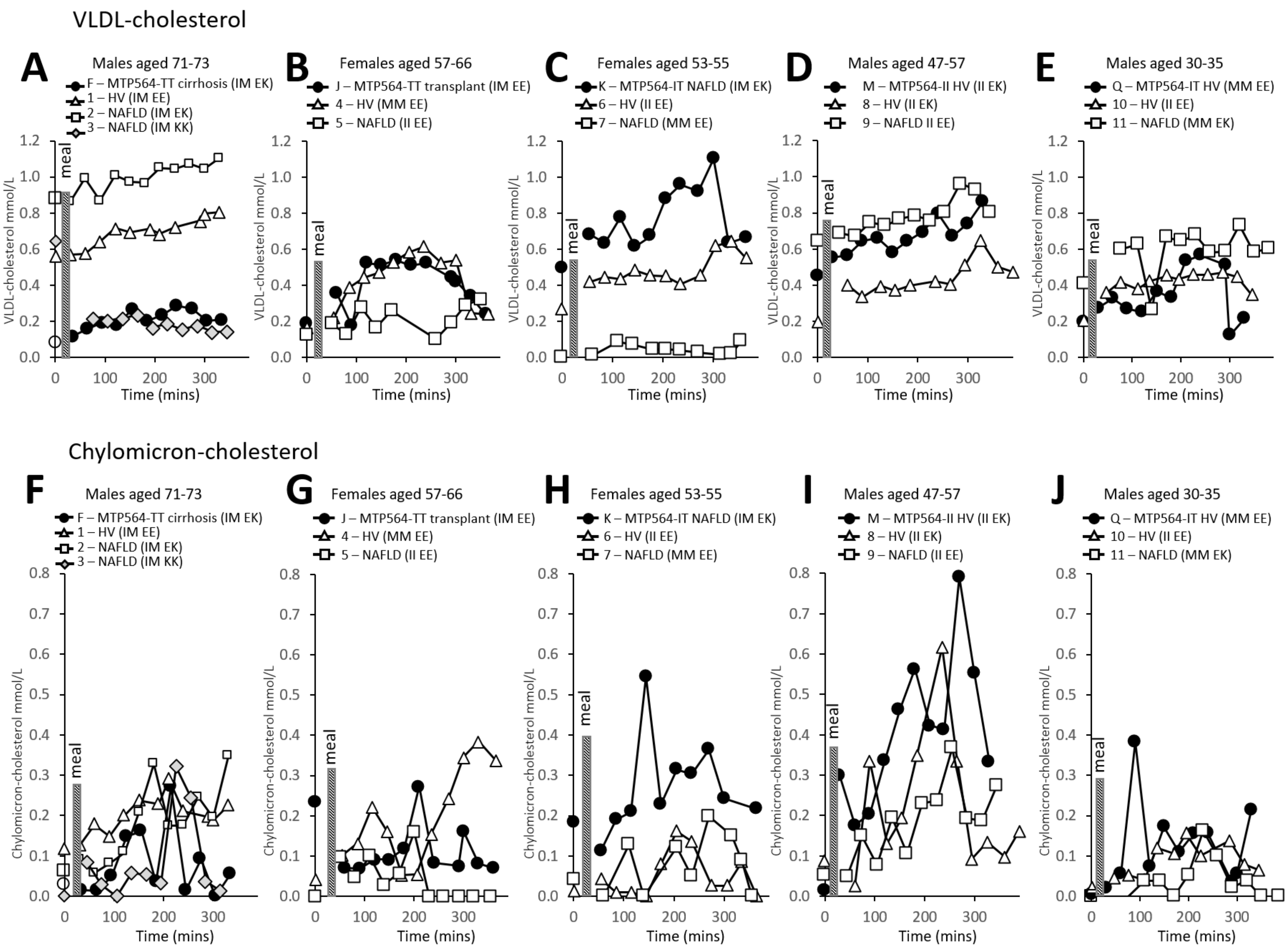
VLDL-cholesterol
A
Males aged 71-73
F – MTP564-TT cirrhosis (IM EK)
1 – HV (IM EE)
2 – NAFLD (IM EK)
3 – NAFLD (IM KK)
B
Females aged 57-66
J – MTP564-TT transplant (IM EE)
4 – HV (MM EE)
5 – NAFLD (II EE)
C
Females aged 53-55
K – MTP564-IT NAFLD (IM EK)
6 – HV (II EE)
7 – NAFLD (MM EE)
D
Males aged 47-57
M – MTP564-II HV (II EK)
8 – HV (II EK)
9 – NAFLD II EE)
E
Males aged 30-35
Q – MTP564-IT HV (MM EE)
10 – HV (II EE)
11 – NAFLD (MM EK)
VLDL-cholesterol mmol/L
meal
Time (mins)
Chylomicron-cholesterol
F
Males aged 71-73
F – MTP564-TT cirrhosis (IM EK)
1 – HV (IM EE)
2 – NAFLD (IM EK)
3 – NAFLD (IM KK)
G
Females aged 57-66
J – MTP564-TT transplant (IM EE)
4 – HV (MM EE)
5 – NAFLD (II EE)
H
Females aged 53-55
K – MTP564-IT NAFLD (IM EK)
6 – HV (II EE)
7 – NAFLD (MM EE)
I
Males aged 47-57
M – MTP564-II HV (II EK)
8 – HV (II EK)
9 – NAFLD (II EE)
J
Males aged 30-35
Q – MTP564-IT HV (MM EE)
10 – HV (II EE)
11 – NAFLD (MM EK)
Chylomicron-cholesterol mmol/L
meal
Time (mins)

Fig. S3. Lipoprotein-associated cholesterol levels in study participants before and after a high fat meal.

Participants are described in Supplemental Table 1. (**A**) VLDL-associated cholesterol levels in MTP564-TT family member F with cirrhosis and matched control participants: 1 (HV), 2 and 3 (NAFLD patients). **(B)** VLDL-cholesterol in MTP564-TT family member J with liver transplantand matched control participants: 4 (HV) and 5 (NAFLD patient). **(C)** VLDL-associated cholesterol levels in MTP564-IT family member K with NAFLD and matched control participants: 6 (HV) and 7 (NAFLD patient). (**D)** VLDL-associated cholesterol levels in MTP564-II (wild type) family member M and matched control participants 8 (HV) and 9 (NAFLD patient). **(E)** VLDL-associated cholesterol levels in MTP564-IT family member Q and matched control participants 10 (HV) and 11 (NAFLD patient). **(F)** Chylomicron-associated cholesterol in participants F, 1, 2 and 3. (**G)** Chylomicron-associated cholesterol in participants J, 4 and 5. (**H)** Chylomicron-associated cholesterol in participants K, 6 and 7. **(I)** Chylomicron-associated cholesterol in participants M, 8 and 9. (**J)** Chylomicron-associated cholesterol in participants Q, 10 and 11.

PNPLA3 p.I148M and *TM6SF2* pE167K genotypes are indicated in parentheses. HV=healthy volunteer; NAFLD=non-alcoholic fatty liver disease.

.

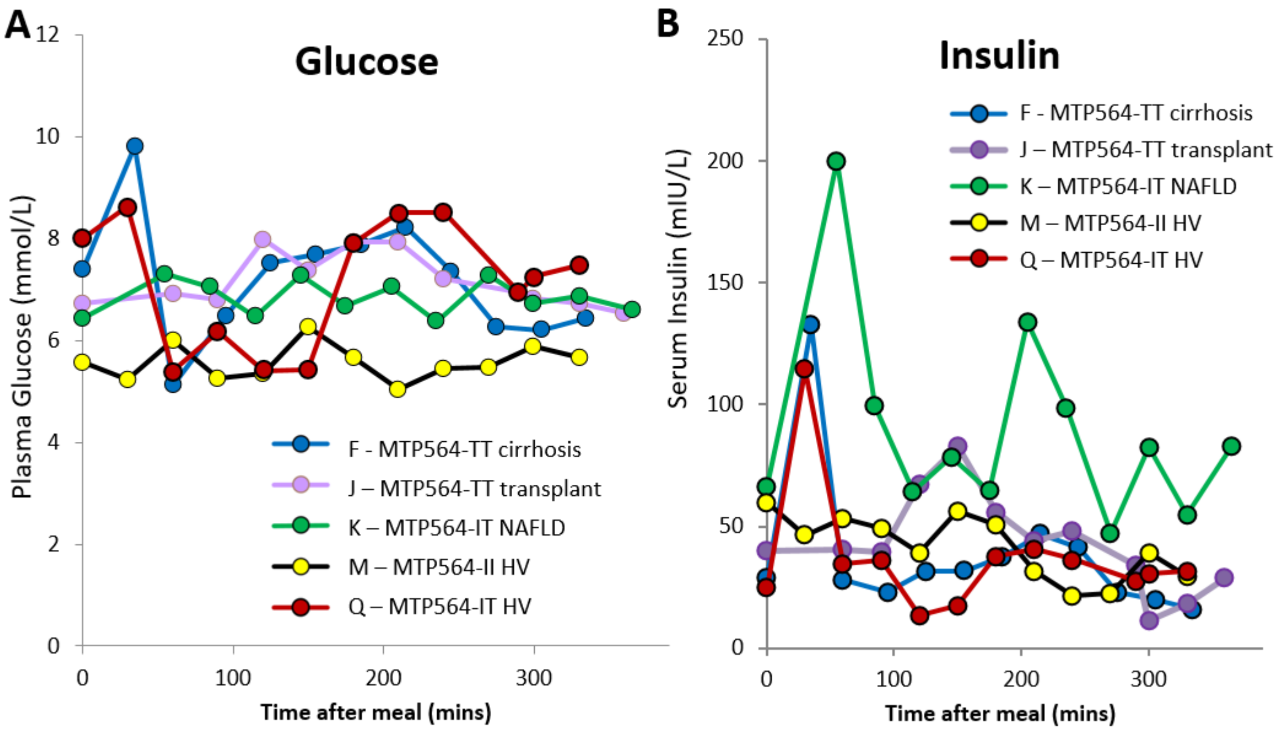

Fig. S4. Blood biomarker levels in family members after consuming a fatty meal.

(**A**) Plasma glucose. (**B**) Serum insulin.

PNPLA3 (rs738409) variant homozygotes (148-MM) and *TM6SF2* (rs58542926) variant homozygotes (167-KK) are indicated. HV=healthy volunteer; NAFLD=non-alcoholic fatty liver disease.

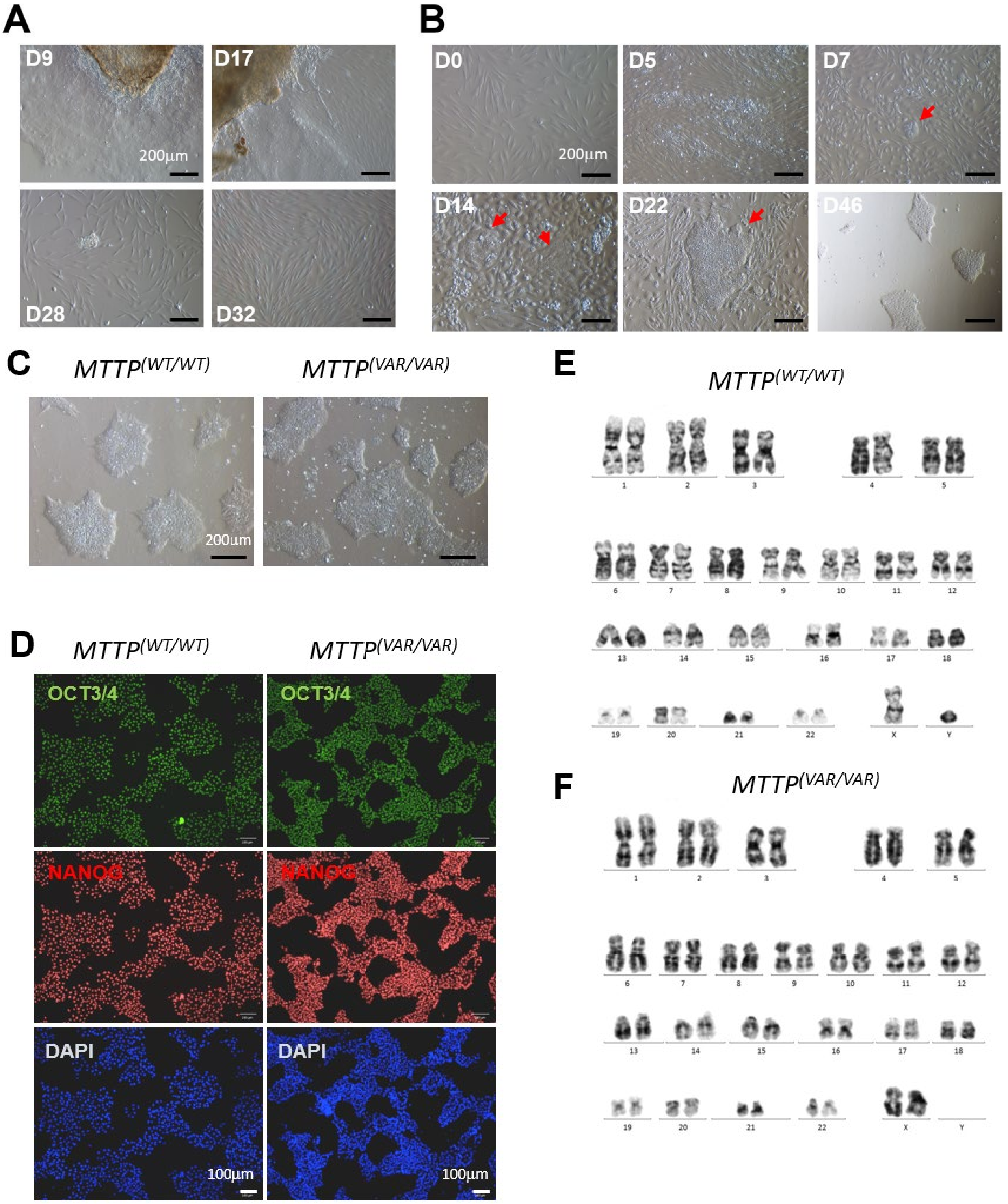

Fig. S5. Characteristics of hiPSCs derived from donor skin biopsy.
(**A**) Light microscopy showing representative skin biopsy fibroblast outgrowth at day 9, 17, 28 and 32. (**B**) Light microscopy showing representative images of fibroblast reprogramming at day 0, 5, 7, 14, 22 and 46 post viral transduction. Red arrows indicate emerging hIPSC colonies. (**C**) Light microscopy showing representative pictures of reprogrammed *MTTP*$^{(WT/WT)}$ and *MTTP*$^{(VAR/VAR)}$ hIPSC cultures. (**D**) Immunocytochemistry showing expression of pluripotency markers in *MTTP*$^{(WT/WT)}$ and *MTTP*$^{(VAR/VAR)}$ hiPSCs. (**E**), Karyotype of *MTTP*$^{(WT/WT)}$. (**F**) Karyotype of *MTTP*$^{(VAR/VAR)}$ hiPSCs.

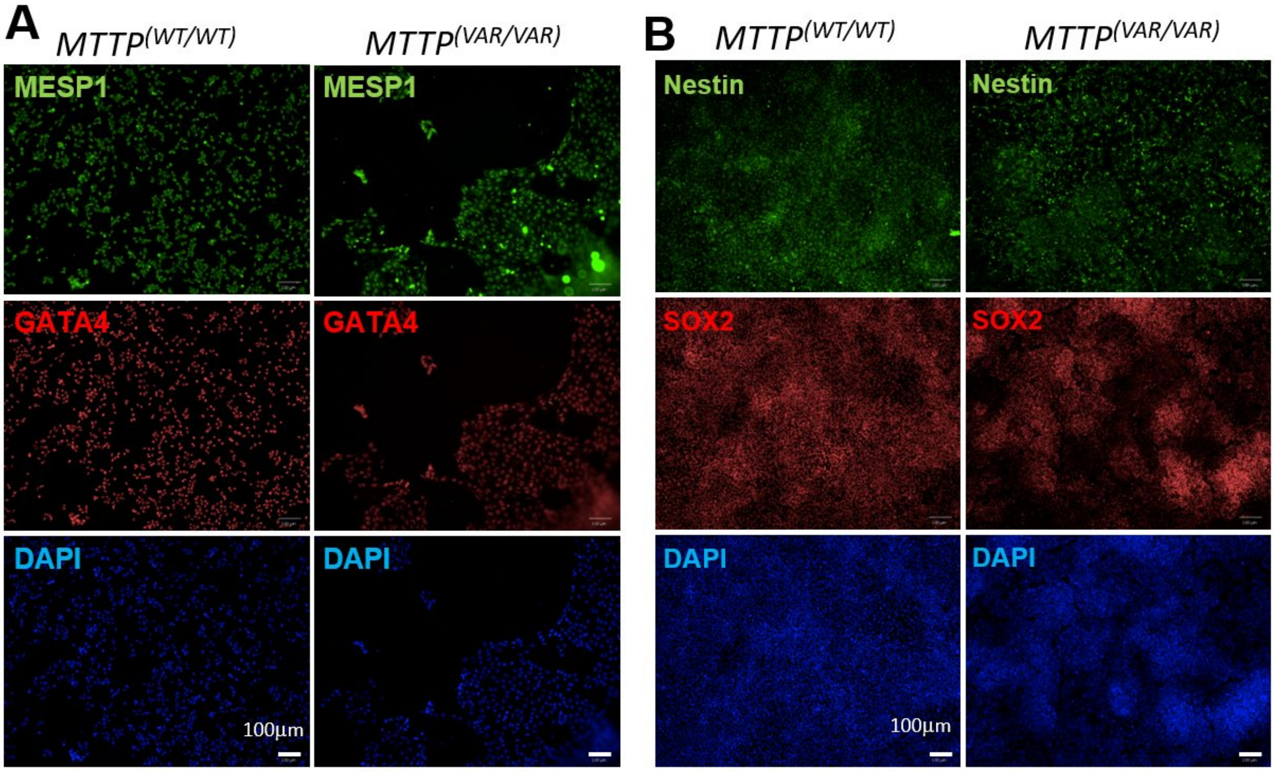

Fig. S6. Differentiation of *MTTP*$^{(WT/WT)}$ and *MTTP*$^{(VAR/VAR)}$ hiPSCs into multiple germ layers. (**A**) Expression of mesodermal genes following mesoderm differentiation of *MTTP*$^{(WT/WT)}$ and *MTTP*$^{(VAR/VAR)}$ hiPSCs by immunocytochemistry with MESP1, GATA4 and DAPI staining. (**B**) Expression of ectoderm genes following ectoderm differentiation of *MTTP*$^{(WT/WT)}$ and *MTTP*$^{(VAR/VAR)}$ hiPSCs by immunocytochemistry with Nestin, SOX2 and DAPI staining.

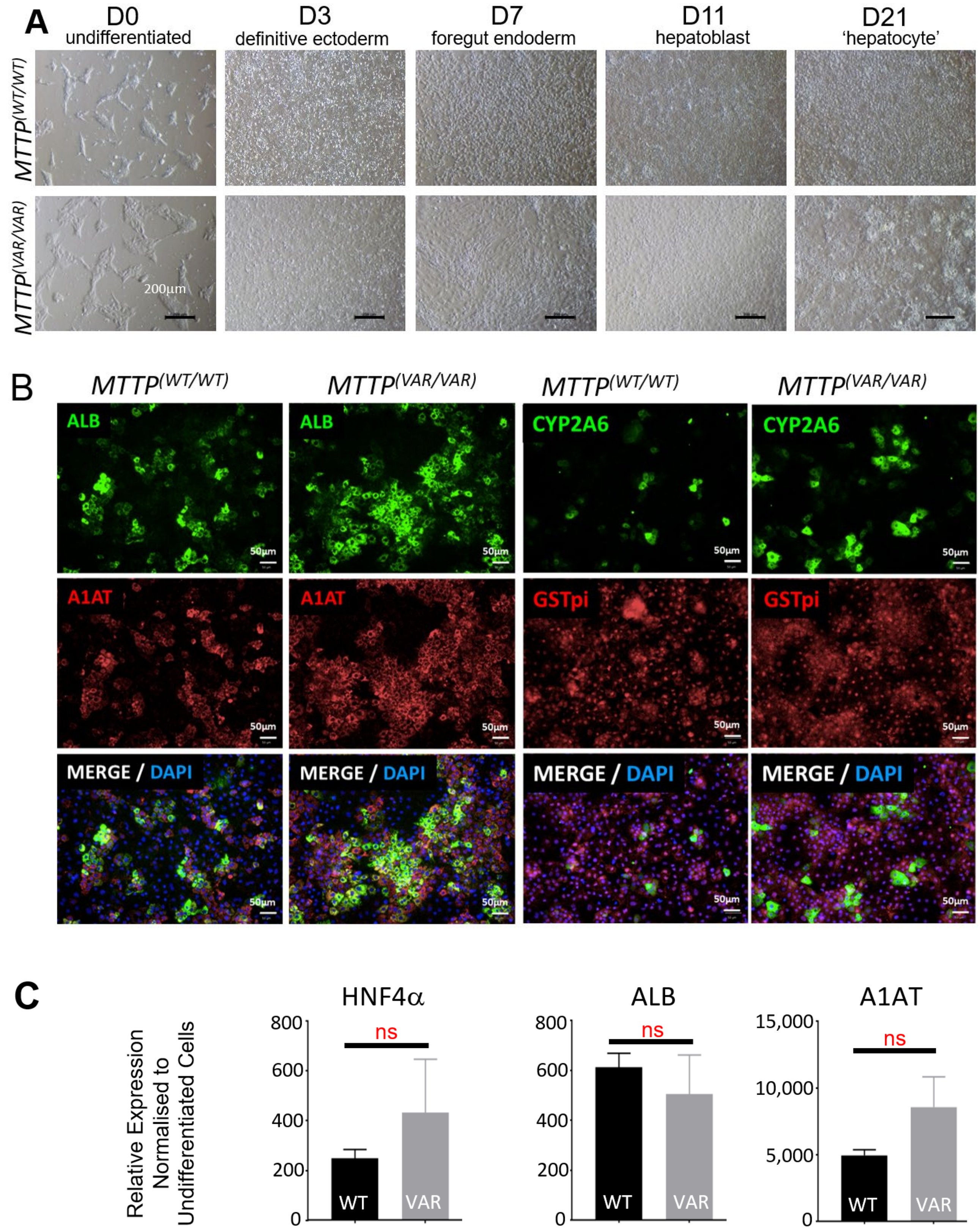

Fig. S7. Differentiation of *MTTP*$^{(WT/WT)}$ and *MTTP*$^{(VAR/VAR)}$ hiPSCs into mature HLCs. **(A)** Representative light microscopy images of *MTTP*$^{(WT/WT)}$ and *MTTP*$^{(VAR/VAR)}$ hiPSCs as they differentiate into HLCs including undifferentiated cells (Day 0), definitive endoderm (Day 3), foregut endoderm (Day 7), hepatoblast (Day 11) and 'hepatocytes' (Day 21). **(B)** Immunocytochemistry showing expression of hepatocyte markers in *MTTP*$^{(WT/WT)}$ and *MTTP*$^{(VAR/VAR)}$ hiPSC derived HLCs. (**C**) Light microscopy of Oil-Red-O stained *MTTP*$^{(WT/WT)}$ and *MTTP*$^{(VAR/VAR)}$ HLCs.

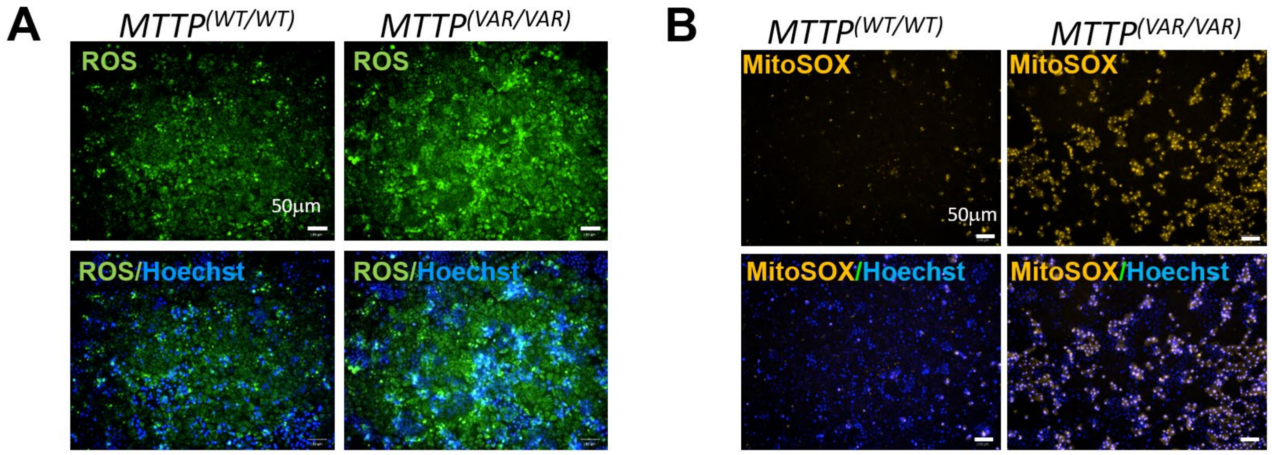

Fig. S8. Assessment of oxidative stress in MTP-564T homozygote variant, MTTP$^{(VAR/VAR)}$ and wild-type hepatocyte-like cells (HLCs).

(A) Reactive oxygen species detected in cells by fluorescence microscopy ±Hoechst stain.

(**B**) Superoxide presence in cells detected by fluorescence microscopy ±Hoechst stain.

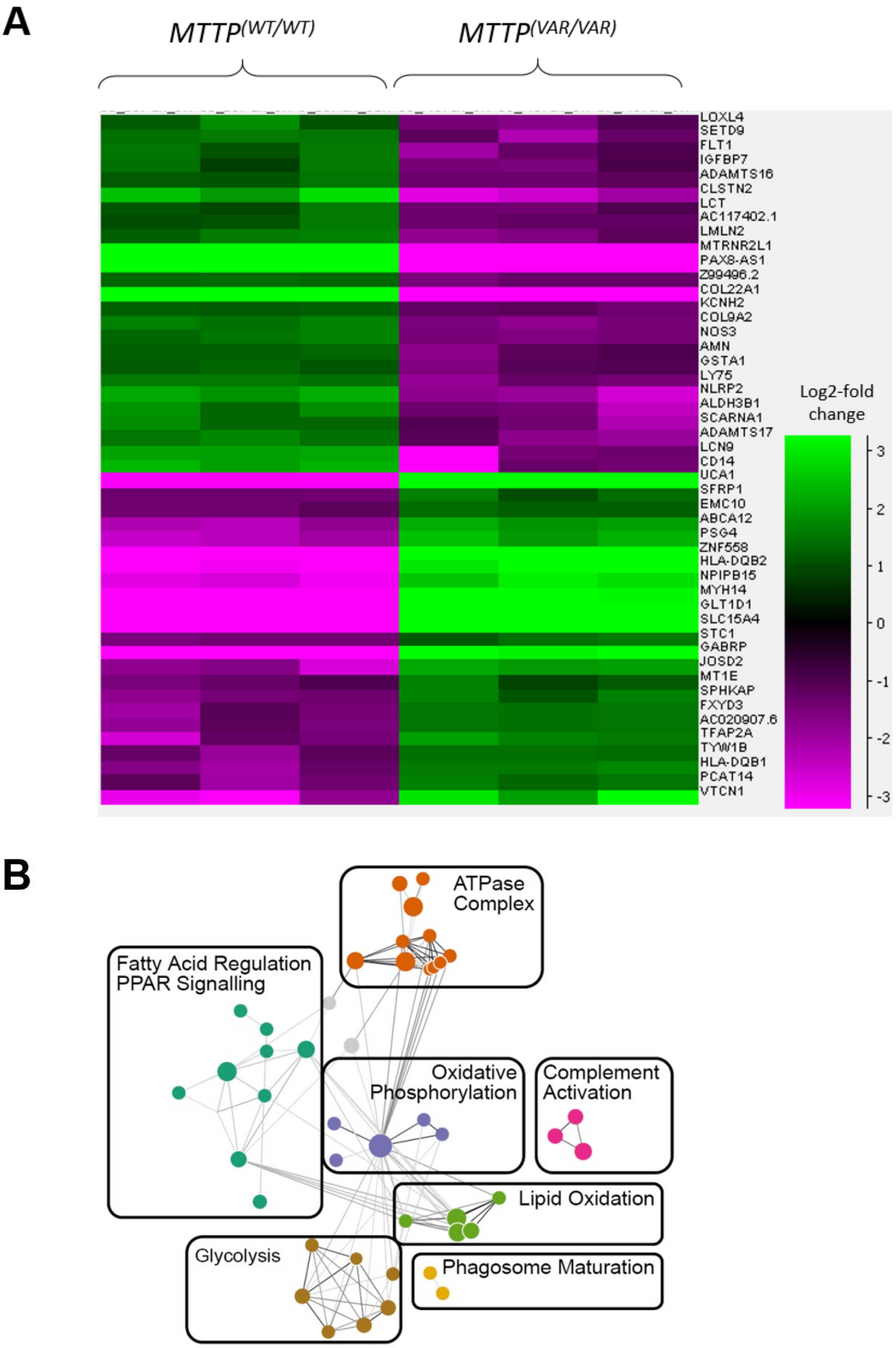

Fig. S9. Comparison of expression patterns in HLCs with MTP564-II and MTP564-TT.
(**A**) Heatmap representing 48 differentially expressed genes with more than 2x log2 fold change in expression, in wild type and MTTP$^{(VAR/VAR)}$ hIPSC-derived HLCs (triplicate samples).
(**B**) Giraph plot from quantitative gene set analysis showing gene sets up-regulated in *MTTP*$^{(VAR/VAR)}$ hIPSC-derived HLCs.

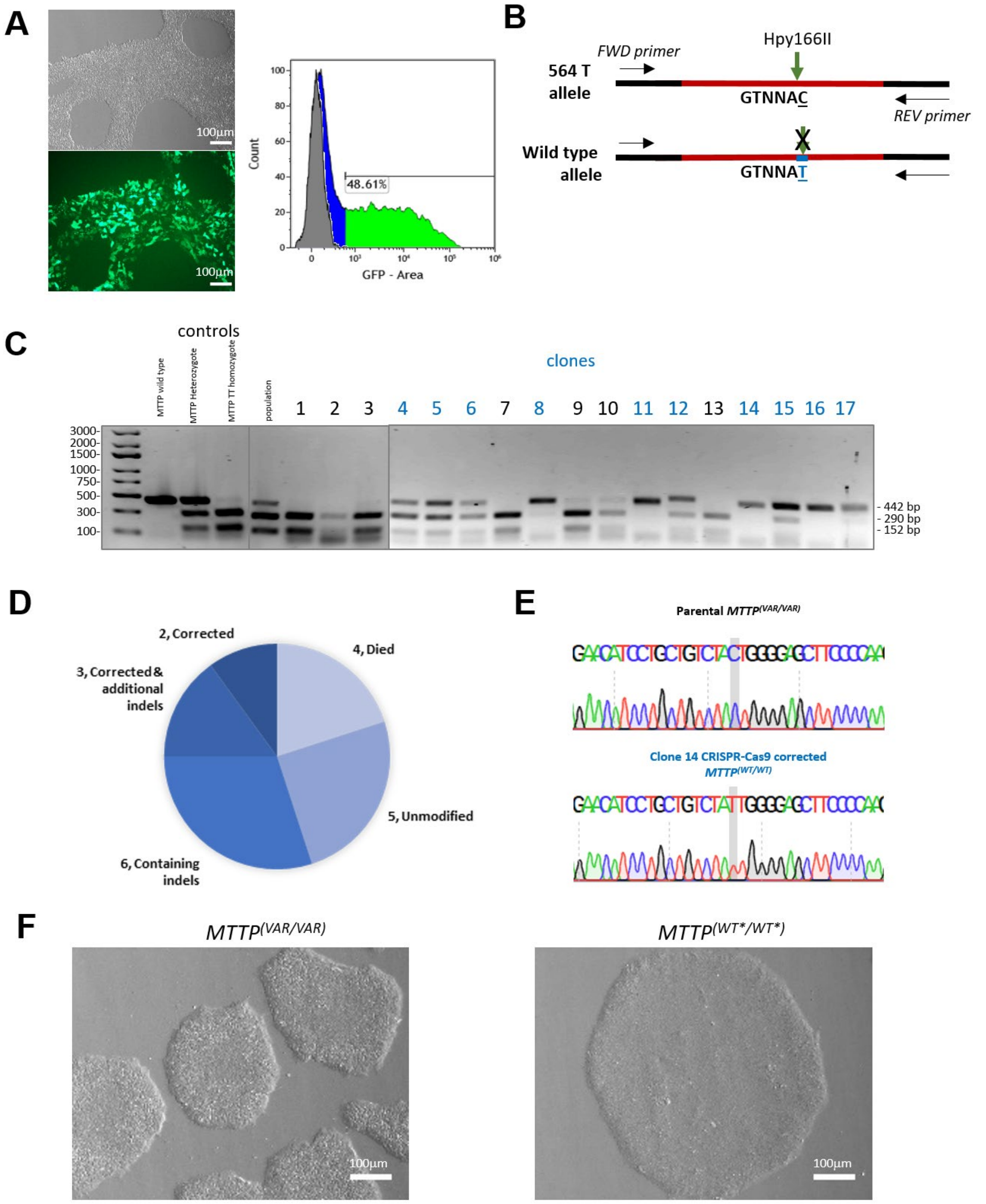

Fig. S10. CRISPR-Cas9 mediated correction of 564TT in the *MTTP*$^{(VAR/VAR)}$ parental line to restore wild type 564II in *MTTP*$^{(WT*/WT*)}$.

(A) Representative phase-contrast and fluorescence microscopy images of MTTP$^{(VAR/VAR)}$ parental line transfected with an EGFP plasmid. Quantification of transfection efficiency by flow cytometry. Untransfected control (grey), sample (blue/green). **(B)** Schematic showing screening strategy for corrected clones. **(C)** Screening and selection of clone 14 by PCR and Hpy166II restriction digestion and agarose gel electrophoresis (O'GeneRuler Express DNA Ladder, Thermofisher). Screening identified 20/39 potentially corrected clones suggesting 51% efficiency. **(D)** Summary of characterisation of clones identified by PCR-RD analysis. Sanger sequencing confirmed 5 clones were unmodified, 6 clones contained indels, 3 were corrected but contained additional indels, giving 5% CRISPR-Cas9 mediated gene-editing efficiency. **(E)** Sanger sequencing chromatograms of parental mutant *MTTP*$^{(VAR/VAR)}$ line and corrected clone 14 *MTTP*$^{(WT*/WT*)}$. **(F)** Representative phase-contrast microscopy images of iPSC cultures for *MTTP*$^{(VAR/VAR)}$ and *MTTP*$^{(WT*/WT*)}$ derived from clone 14.

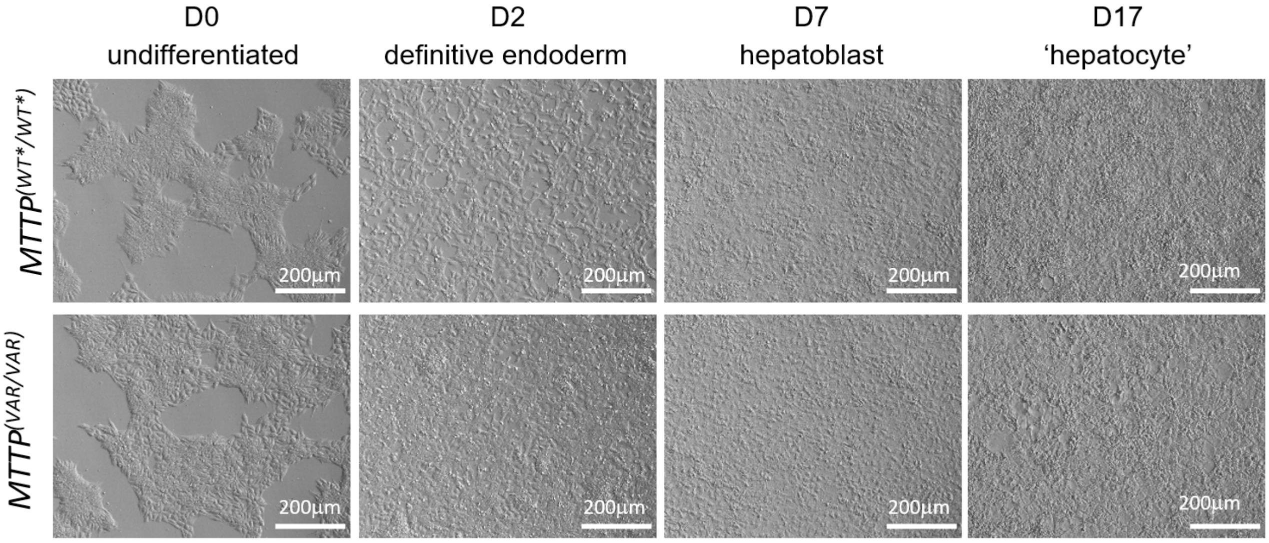

Fig. S11. **Differentiation of CRISPR-Cas9 corrected *MTTP*$^{(WT/WT)}$ and parental *MTTP*$^{(VAR/VAR)}$ hiPSCs into mature HLCs**.

Representative phase-contrast microscopy images of CRISPR-Cas9 corrected *MTTP*$^{(WT/WT)}$ and parental *MTTP*$^{(VAR/VAR)}$ hiPSCs during the differentiation into hiPSC-derived hepatocyte-like cells (HLCs) including undifferentiated cells (Day 0), definitive endoderm (Day 2), hepatoblasts (Day 7) and 'hepatocytes' (Day 17).

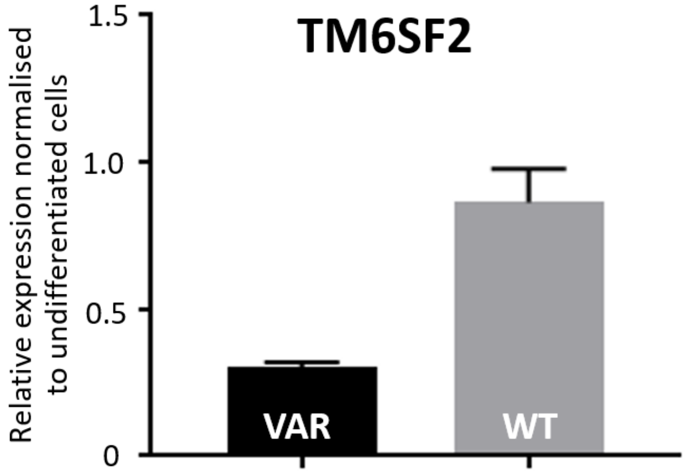

Fig. S12. Assessment of TM6SF2 expression in *MTTP*$^{(VAR/VAR)}$ and restored wild type 564II *MTTP*$^{(WT*/WT*)}$.

Quantification of TM6SF2 mRNA levels by QPCR in *MTTP*$^{(VAR/VAR)}$ and *MTTP*$^{(WT*/WT*)}$ derived hepatocytes.

Supplemental Tables:

Table S1. Characteristics of study participants included in meal response analysis. *PNPLA3* p.I148M and *TM6SF2* p.E167K genotypes are shown. NAFLD, Non-alcoholic fatty liver disease; NASH, non-alcoholic steatohepatitis.

**Family member studied**		**Matched healthy volunteer**		**Matched NAFLD patient**	
**Person ID-Family**	Description	**Person ID-Healthy Volunteer**	Description	**Person ID-NAFLD case**	Description
**F**	♂ 71y, BMI 21.4, MTP564-TT cirrhosis, PNPLA3-IM TM6SF2-EK	**1**	♂ 73y, BMI 29.6, PNPLA3-IM TM6SF2-EE	**2**	♂ 73y, BMI 31.9, NASH + fibrosis PNPLA3-IM TM6SF2-EK
				**3**	♂ 71y, BMI 25.3, NASH + fibrosis PNPLA3-IM TM6SF2-KK
**J**	♀ 57y, BMI 20.3, MTP564-TT healthy transplanted PNPLA3-IM TM6SF2-EE	**4**	♀ 63y, BMI 27.0, PNPLA3-MM TM6SF2-EE	**5**	♀ 66y, BMI 26.6, NASH no fibrosis, PNPLA3-II TM6SF2-EE
**K**	♀ 55y, BMI 24.5, MTP564-IT fatty liver PNPLA3-IM TM6SF2-EK	**6**	♀ 57y, BMI 25.3, PNPLA3-II TM6SF2-EE	**7**	♀ 57y, BMI 41.5, NASH + fibrosis, PNPLA3-MM TM6SF2-EE
**M**	♂ 48y, BMI 25.4, MTP564-II healthy PNPLA3-II TM6SF2-EK	**8**	♂ 57y, BMI 29.5, PNPLA3 II TM6SF2 EK	**9**	♂ 47y, BMI 42.0, NASH + fibrosis, diabetic PNPLA3-II TM6SF2-EE
**Q**	♂ 31y, BMI 22.8, MTP564-IT healthy PNPLA3-MM TM6SF2-EE	**10**	♂ 35y, BMI 29.0, PNPLA3-II TM6SF2-EE	**11**	♂ 30y, BMI 39.9, NASH + fibrosis PNPLA3-MM TM6SF2-EK

Table S2. All RNA-seq and CHIP-seq Sample Search Space (ARCHS4) tissue type and cell type predictions for hIPSC-derived hepatocyte-like cells.

Index		MTP564-II wild type hIPSC derived hepatocyte-like cells					MTP564-TT variant hIPSC derived hepatocyte-like cells				
		Name	P-value	Adjusted P-value	Odds ratio	Combined Score	Name	P-value	Adjusted P-value	Odds ratio	Combined Score
Tissue Type Prediction	1	Liver (bulk tissue)	6.662e-163	7.194e-161	2.72	1017.19	Liver (bulk tissue)	2.363e-171	2.552e-169	2.74	1075.07
	2	Hepatocyte	3.025e-118	1.633e-116	2.44	661.17	Hepatocyte	4.413e-123	2.383e-121	2.45	689.05
	3	Small intestine (bulk tissue)	1.268e-78	4.564e-77	2.15	385.83	Small intestine (bulk tissue)	4.644e-82	1.672e-80	2.16	403.60
	4	Gastric epithelial cell	9.568e-70	2.583e-68	2.08	330.09	Ileum (bulk tissue)	4.053e-68	1.094e-66	2.04	316.85
	5	Ileum (bulk tissue)	4.833e-67	1.044e-65	2.05	313.59	Lung (bulk tissue)	1.365e-61	2.948e-60	1.99	278.23
	6	Lung (bulk tissue)	5.792e-58	1.042e-56	1.97	259.88	Gastric epithelial cell	1.754e-59	3.158e-58	1.97	266.04
	7	Colon (bulk tissue)	1.893e-53	2.921e-52	1.93	234.18	Colon (bulk tissue)	2.340e-53	3.610e-52	1.91	231.44
	8	Gastric tissue (bulk)	4.762e-46	6.428e-45	1.85	193.58	Gastric tissue (bulk)	3.668e-47	4.952e-46	1.85	197.74
	9	Omentum	3.685e-39	4.422e-38	1.78	157.60	Omentum	6.260e-45	7.512e-44	1.83	185.93
	10	Amniotic fluid	1.454e-23	1.570e-22	1.59	83.40	Skin (bulk tissue)	5.090e-28	5.497e-27	1.63	102.71
Cell	1	HEPG2	9.067e-83	1.133e-80	2.16	407.91	HEPG2	1.419e-75	1.774e-73	2.08	358.54
	2	HUH7	5.774e-52	3.609e-50	1.90	223.63	HUH7	5.473e-42	3.421e-49	1.87	216.27

	3	HEP3B	1.702e-41	7.091e-40	1.79	168.03	HEP3B	9.101e-42	3.754e-40	1.78	167.99
	4	CFPAC1	1.049e-38	3.278e-37	1.76	153.90	CFPAC1	2.518e-38	7.870e-37	1.74	150.72
	5	CAPAN1	5.244e-36	1.311e-34	1.73	140.51	MCF10	2.035e-33	5.088e-32	1.69	126.93
	6	A549	1.156e-29	2.409e-28	1.65	110.23	CAPAN1	3.661e-32	7.626e-31	1.67	121.00
	7	BXPC3	7.111e-28	1.270e-26	1.63	102.00	A549	7.477e-32	1.335e-30	1.67	119.54
	8	MCF10	2.724e-25	4.257e-24	1.60	90.38	BXPC3	4.377e-27	6.840e-26	1.61	97.69
	9	RT4	1.230e-23	1.709e-22	1.58	83.09	RT4	1.444e-24	2.006e-23	1.58	86.55
	10	HT29	2.676e-22	3.345e-21	1.56	77.31	HNSCC	1.740e-23	2.175e-22	1.56	81.86

Table S3. Gene set analysis showing terms for all genes differentially expressed between MTP564-II wild type and MTP564-TT variant hIPSC-derived hepatocyte-like cells.

source	term_name	term_id	adjusted_p_value	negative_log10_of_adjusted_p_value
REAC	Extracellular matrix organization	REAC:R-HSA-1474244	4.87E-07	6.312667258
GO:CC	extracellular matrix	GO:0031012	2.85941E-06	5.543723748
REAC	Degradation of the extracellular matrix	REAC:R-HSA-1474228	2.43072E-05	4.614264431
GO:BP	extracellular matrix organization	GO:0030198	0.000831626	3.080071732
GO:BP	extracellular structure organization	GO:0043062	0.000894227	3.048552438
REAC	Collagen formation	REAC:R-HSA-1474290	0.003136295	2.503583115
GO:BP	digestion	GO:0007586	0.004959299	2.304579689
GO:CC	collagen-containing extracellular matrix	GO:0062023	0.00516165	2.287211438
KEGG	Protein digestion and absorption	KEGG:04974	0.006978005	2.156268728
REAC	Collagen biosynthesis and modifying enzymes	REAC:R-HSA-1650814	0.014030082	1.852939779
KEGG	ECM-receptor interaction	KEGG:04512	0.014057765	1.852083733
REAC	ECM proteoglycans	REAC:R-HSA-3000178	0.016110075	1.792902441
TF	Factor: slug; motif: NRCAGGTGCR; match class: 1	TF:M12259_1	0.027606305	1.558991718

Table S4. Gene set analysis showing terms for genes upregulated in MTP564-II wild type hIPSC derived hepatocyte-like cells compared to MTP564-TT variant hIPSC-derived hepatocyte-like cells.

source	term_name	term_id	adjusted_p_value	negative_log10_of_adjusted_p_value
GO:CC	midbody	GO:0030496	0.014234541	1.846656538
REAC	Resolution of Sister Chromatid Cohesion	REAC:R-HSA-2500257	0.018667177	1.728921362
REAC	Amplification of signal from unattached kinetochores via a MAD2 inhibitory signal	REAC:R-HSA-141444	0.023621746	1.626688011
REAC	Amplification of signal from the kinetochores	REAC:R-HSA-141424	0.023621746	1.626688011
GO:CC	spindle	GO:0005819	0.026656468	1.5741974

Table S5. Gene set analysis showing terms for genes upregulated in MTP564-TT variant hIPSC derived hepatocyte-like cells compared to MTP564-II wild type hIPSC-derived hepatocyte-like cells.

source	term_name	term_id	adjusted_p_value	negative_log10_of_adjusted_p_value
REAC	Extracellular matrix organization	REAC:R-HSA-1474244	0.000145038	3.838517822
REAC	Degradation of the extracellular matrix	REAC:R-HSA-1474228	0.000353562	3.451534414
REAC	ECM proteoglycans	REAC:R-HSA-3000178	0.002952027	2.529879617
GO:BP	extracellular matrix organization	GO:0030198	0.008885339	2.051326005
GO:BP	extracellular structure organization	GO:0043062	0.009318155	2.030670061
HP	Abnormal cardiovascular system physiology	HP:0011025	0.014922899	1.826146793
KEGG	ECM-receptor interaction	KEGG:04512	0.01921473	1.71636572
KEGG	Focal adhesion	KEGG:04510	0.027077396	1.567393098
WP	Focal Adhesion	WP:WP306	0.030961443	1.509178802
HP	Osteoporosis	HP:0000939	0.038409054	1.415566389
HP	Abnormality of skin physiology	HP:0011122	0.043372457	1.362785975
HP	Abnormality of humoral immunity	HP:0005368	0.043717346	1.35934621
HP	Abnormal vascular physiology	HP:0030163	0.04508747	1.345944131

Supplemental References

Author names in bold designate shared co-first authorship.

[1] Simpson EJ, Debevec T, Eiken O, Mekjavic I, Macdonald IA. PlanHab: the combined and separate effects of 16 days of bed rest and normobaric hypoxic confinement on circulating lipids and indices of insulin sensitivity in healthy men. Journal of applied physiology 2016;120:947-955.

[2] Havel RJ, Eder HA, Bragdon JH. The distribution and chemical composition of ultracentrifugally separated lipoproteins in human serum. The Journal of clinical investigation 1955;34:1345-1353.

[3] **John C, Reeve NF**, Free RC, Williams AT, Ntalla I, Farmaki A-E, et al. Cohort Profile: Extended Cohort for E-health, Environment and DNA (EXCEED). International Journal of Epidemiology 2019;48:678-679j.

[4] Li H, Durbin R. Fast and accurate short read alignment with Burrows-Wheeler transform. Bioinformatics 2009;25:1754-1760.

[5] **Li H, Handsaker B**, Wysoker A, Fennell T, Ruan J, Homer N, et al. The Sequence Alignment/Map format and SAMtools. Bioinformatics 2009;25:2078-2079.

[6] Van der Auwera GA, Carneiro MO, Hartl C, Poplin R, Del Angel G, Levy-Moonshine A, et al. From FastQ data to high confidence variant calls: the Genome Analysis Toolkit best practices pipeline. Curr Protoc Bioinformatics 2013;43:11.10.11-11.10.33.

[7] https://www.internationalgenome.org. Accessed 15 March 2015

[8] ftp://ftp.1000genomes.ebi.ac.uk/vol1/ftp/release/20130502/supporting/hd_genotype_chip/. Accessed 15 March 2015

[9] https://www.ncbi.nlm.nih.gov/snp/. Accessed 15 March 2015.

[10] https://www.sanger.ac.uk/resources/downloads/human/hapmap3.html. Accessed 15 March 2014

[11] https://evs.gs.washington.edu/EVS/. Accessed 13 March 2015.

[12] **Chambers JC, Abbott J, Zhang W, Turro E,** Scott WR, Tan ST, et al. The South Asian genome. PloS one 2014;9:e102645.

[13] Wang K, Li M, Hakonarson H. ANNOVAR: functional annotation of genetic variants from high-throughput sequencing data. Nucleic Acids Research 2010;38:e164-e164.

[14] McLaren W, Gil L, Hunt SE, Riat HS, Ritchie GRS, Thormann A, et al. The Ensembl Variant Effect Predictor. Genome Biology 2016;17:122.

[15] Vaser R, Adusumalli S, Leng SN, Sikic M, Ng PC. SIFT missense predictions for genomes. Nat Protoc 2016;11:1-9.

[16] **Adzhubei IA, Schmidt S, Peshkin L,** Ramensky VE, Gerasimova A, Bork P, et al. A method and server for predicting damaging missense mutations. Nat Methods 2010;7:248-249.

[17] **Kircher M, Witten DM,** Jain P, O'Roak BJ, Cooper GM, Shendure J. A general framework for estimating the relative pathogenicity of human genetic variants. Nat Genet 2014;46:310-315.

[18] Ritchie GR, Dunham I, Zeggini E, Flicek P. Functional annotation of noncoding sequence variants. Nat Methods 2014;11:294-296.

[19] Kelley LA, Mezulis S, Yates CM, Wass MN, Sternberg MJE. The Phyre2 web portal for protein modeling, prediction and analysis. Nature protocols 2015;10:845-858.

[20] Yates CM, Filippis I, Kelley LA, Sternberg MJ. SuSPect: enhanced prediction of single amino acid variant (SAV) phenotype using network features. Journal of molecular biology 2014;426:2692-2701.

[21] Grove JI, Thiagarajan P, Astbury S, Harris R, Delahooke T, Guha IN, et al. Analysis of genotyping for predicting liver injury marker, procollagen III in persons at risk of non-alcoholic fatty liver disease. Liver international : official journal of the International Association for the Study of the Liver 2018;38:1832-1838.

[22] Bertero A, Pawlowski M, Ortmann D, Snijders K, Yiangou L, Cardoso de Brito M, et al. Optimized inducible shRNA and CRISPR/Cas9 platforms for in vitro studies of human development using hPSCs. Development 2016;143:4405.

[23] Vallier L, Touboul T, Chng Z, Brimpari M, Hannan N, Millan E, et al. Early cell fate decisions of human embryonic stem cells and mouse epiblast stem cells are controlled by the same signalling pathways. PloS one 2009;4:e6082.

[24] Hannan NR, Fordham RP, Syed YA, Moignard V, Berry A, Bautista R, et al. Generation of multipotent foregut stem cells from human pluripotent stem cells. Stem cell reports 2013;1:293-306.

[25] Ran FA, Hsu PD, Wright J, Agarwala V, Scott DA, Zhang F. Genome engineering using the CRISPR-Cas9 system. Nature protocols 2013;8:2281-2308.

[26] Shen B, Zhang W, Zhang J, Zhou J, Wang J, Chen L, et al. Efficient genome modification by CRISPR-Cas9 nickase with minimal off-target effects. Nature methods 2014;11:399-402.

[27] Priddle H, Allegrucci C, Burridge P, Munoz M, Smith NM, Devlin L, et al. Derivation and characterisation of the human embryonic stem cell lines, NOTT1 and NOTT2. In vitro cellular & developmental biology Animal 2010;46:367-375.

[28] Rimland CA, Tilson SG, Morell CM, Tomaz RA, Lu WY, Adams SE, et al. Regional Differences in Human Biliary Tissues and Corresponding In Vitro-Derived Organoids. Hepatology 2021;73:247-267.

[29] Segeritz CP, Rashid ST, de Brito MC, Serra MP, Ordonez A, Morell CM, et al. hiPSC hepatocyte model demonstrates the role of unfolded protein response and inflammatory networks in α(1)-antitrypsin deficiency. Journal of hepatology 2018;69:851-860.

[30] Kuleshov MV, Jones MR, Rouillard AD, Fernandez NF, Duan Q, Wang Z, et al. Enrichr: a comprehensive gene set enrichment analysis web server 2016 update. Nucleic acids research 2016;44:W90-97.
