# Supplementary material for: Identification and characterisation of a rare MTTP variant underlying hereditary non-alcoholic fatty liver disease
Source: JHEP Rep. 2023 Apr 23;5(8):100764. doi: 10.1016/j.jhepr.2023.100764 (PMC10362796; doi:10.1016/j.jhepr.2023.100764)
Supplement: Multimedia component 2 [file mmc2.docx]

**Journal of Hepatology**

**CTAT methods**

Tables for a “Complete, Transparent, Accurate and Timely account” (CTAT) are now mandatory for all revised submissions. The aim is to enhance the reproducibility of methods.

- Only include the parts relevant to your study
- Refer to the CTAT in the main text as ‘Supplementary CTAT Table’
- Do not add subheadings
- Add as many rows as needed to include all information
- Only include one item per row

**If the CTAT form is not relevant to your study, please outline the reasons why:**

|  |
| --- |

- 1. **Antibodies**

| **Name** | **Citation** | **Supplier** | **Cat no.** | **Clone no.** |
| --- | --- | --- | --- | --- |
| MTTP |  | Abcam | #ab63467 |  |
| OCT3/4 |  | Santa Cruz Biotechnology | #sc-5279 |  |
| NANOG |  | R&D Systems | #AF1997 |  |
| MESP1 |  | Abcam | #ab129387 |  |
| GATA-4 |  | R&D Systems | #AF2606 |  |
| Nestin |  | Merck | #MAB5326 |  |
| SOX2 |  | Novus Biologicals | #NB110-37235 |  |
| ALB |  | R&D Systems | #MAB1455 |  |
| CYP2A6 |  | OriGene Technologies | #TA503832 |  |
| A1AT |  | Abcam | #ab179443 |  |
| GST-pi |  | Enzo Life Sciences | #ADI-MSA-102-E |  |
| Donkey Anti-Mouse AF 488 (1/400 dilution) |  | Invitrogen (Thermo Fisher) | #A21202 |  |
| Donkey Anti-Rabbit AF 647 (1/400 dilution) |  | Invitrogen (Thermo Fisher) | #A31573 |  |
| Donkey Anti-Goat AF 647 (1/400 dilution) |  | Invitrogen (Thermo Fisher) | #A21447 |  |

- 1. **Cell lines**

| **Name** | **Citation** | **Supplier** | **Cat no.** | **Passage no.** | **Authentication test method** |
| --- | --- | --- | --- | --- | --- |
|  |  |  |  |  |  |

- 1. **Organisms**

| **Name** | **Citation** | **Supplier** | **Strain** | **Sex** | **Age** | **Overall n number** |
| --- | --- | --- | --- | --- | --- | --- |
|  |  |  |  |  |  |  |

- 1. **Sequence based reagents**

| **Name** | **Sequence** | **Supplier** |
| --- | --- | --- |
| *MTTP*-F1 | tcttaacggcctcagcctag | Sigma |
| *MTTP*-R1 | cagagttaccagtcatggactc | Sigma |
| *MTTP*-F2 | GGCTTGCTAGTGTGCTAATGACAG | Sigma |
| *MTTP*-R2 | GAGTGACCCTCTTCAGAACCTGC | Sigma |
| *MTTP*-F5 | AAGGTAGAATAGGGCAGGGGTCC | Sigma |
| *MTTP*-R5 | CTAATCTCAGTTGGATCATTTCAGTCTC | Sigma |
| *MTTP*-F6 | GTTACAGGTAGAGAACATGCTGACATG | Sigma |
| *MTTP*-R6 | CCTCCATGGTACAGTGGTGCAC | Sigma |
| *MTTP*-F7 | CAGTCACAGAGTCCTACCCAGG | Sigma |
| *MTTP*-R7 | GAGACTGCTGTCATCACAACTCTGTG | Sigma |
| PNPLA3-6R | CAGCTGTGGCTACTCTGTCTG | Sigma |
| PNPLA3-4F | TGGAGAAAGCTTATGAAGGATCAG | Sigma |
| GCKR-2F | GGGTCTTAGGGTACCTGCTCAGAGG | Sigma |
| GCKR-2R | GGTAACCCATGACCTTGCCCAGC | Sigma |
| TM6SF2-2F | Ccaaaatgttgggattacagg | Sigma |
| TM6SF2-2R | Acagatgtccagcagggttc | Sigma |
| FWD | GTTTGAACATCTTATGAACAGGTG | Sigma |
| REV | CAGAGTTACCAGTCATGGACTC | Sigma |
| TM6SF2-F | CGGTCTACAGCTTGTCCCAT | Sigma |
| TM6SF2-R | GGTATGGCTCTCCCTCCTTG | Sigma |

- 1. **Biological samples**

| **Description** | **Source** | **Identifier** |
| --- | --- | --- |
|  |  |  |

- 1. **Deposited data**

| **Name of repository** | **Identifier** | **Link** |
| --- | --- | --- |
|  |  |  |

- 1. **Software**

| **Software name** | **Manufacturer** | **Version** |
| --- | --- | --- |
| Picard | http://broadinstitute.github.io/picard/ | 1.93 |
| SAMtools | *Bioinformatics.* 2009;25(16):2078-2079 | 1.1 |
| GATK | https://gatk.broadinstitute.org/ | 3.2-2 |
| BWA | Bioinformatics. 2009;25(14):1754-1760 | 0.7.5 |
| ANNOVAR | *Nucleic Acids Research.* 2010;38(16):e164-e164. | November 2014 |
| Effect Predictor | *https://www.ensembl.org/info/docs/tools/vep/index.html#vep_tools* | November 2014 |
| Hisat2 | http://daehwankimlab.github.io/hisat2/ | v2.1.0 |
| SeqMonk | The Babraham Institute | 1.46.0 |
| DESeq2 | Bioconductor | 1.28.1 |
| Trim Galore | @FelixKrueger, The Babraham Institute | 0.6.2 |
| Graph Pad Prism | La Jolla | 8 |
| Protein Homology/analogy Recognition Engine (Phyre2). | Structural Bioinformatics Group, Imperial College, London | 2.0 |
| Visual Molecular Dynamics (VMD) | Theoretical and Computational Biophysics Group, University of Illinois, Urbana-Champaign | 1.9.3 |
| Image Studio Lite | Licor | 5.2 |
| Columbus™ | PerkinElmer |  |
|  |  |  |

- 1. **Other (e.g. drugs, proteins, vectors etc.)**

| **Name** | **Cat. No.** | **Supplier** |
| --- | --- | --- |
| Taq polymerase | M02675 | New England Biolabs |
| dNTP | N0447S | New England Biolabs |
| QIAquick PCR Purification Kit | 28104 | Qiagen |
| Agarose | A6877 | Sigma |
| Tris | 15430167 | Fisher Scientific |
| Boric Acid | 10011083 | Fisher Scientific |
| EDTA | BP2482-500 | Fisher Scientific |
| FokI | R0109S | New England Biolabs |
| Hpy166II | R0616S | New England Biolabs |
| MspI | R0106S | New England Biolabs |
| Hpy188I | R0617S | New England Biolabs |
| NimbleGen SeqCap EZ Exome v3.0 | n/a | Roche |
| ABX Pentra Apo B reagent | A11A01688 | Horiba ABX |
| Cholesterol BP diagnostic reagent | A11A01634 | Horiba ABX |
| HDL Direct CP reagent | A11A01636 | Horiba ABX |
| LDL Direct CP reagent | A11A01638 | Horiba ABX |
| Glucose PAP CP reagent | A11A01668 | Horiba ABX |
| Triglycerides CP reagent | A11A01640 | Horiba ABX |
| ethylene glycol tetraacetic acid | E4378 | Sigma |
| glutathione | G4251 | Sigma |
| tetrahydrolipstatin | Xenical 120mg Orlistat | Roche |
| Free fatty acids assay | NEFA C | Wako |
| Insulin RIA | HI-14K | Merck Millipore |
| ApoB ELISA | RAB0610 | Sigma |
| MitoTracker green FM | M7514 | Invitrogen (ThermoFisher) |
| MitoTracker deep red FM | M22426 | Invitrogen (ThermoFisher) |
| CellROX green | C10444 | Invitrogen (ThermoFisher) |
| MitoSox Red | M36008 | Invitrogen (ThermoFisher) |
| Hoechst 33342 | H3570 | Invitrogen (ThermoFisher) |
| DAPI | D9542 | Sigma |
| Nile Red | N1142 | Invitrogen (ThermoFisher) |
| foetal bovine serum | A5256701 | Gibco (ThermoFisher) |
| TritonX-100 | A16046 | ThermoFisher |
| PBS | 10010023 | Gibco (ThermoFisher) |
| DMEM | 11966 | Gibco (ThermoFisher) |
| Antibiotic-Antimycotic | 15240096 | Gibco (ThermoFisher) |
| GlutaMAX | 35050061 | Gibco (ThermoFisher) |
| NEAA | 11140068 | Gibco (ThermoFisher) |
| penicillin/streptomycin | 15070063 | Gibco (ThermoFisher) |
| Trypsin-EDTA | R001100 | Gibco (ThermoFisher) |
| EZ-PCR Mycoplasma Test Kit | 20-700-20 | Biological Industries |
| Human NFκB Pathway, Array | ARY029 | R&D Systems |
| Human, Phospho-Kinase Array | ARY003C | R&D Systems |
| Human XL Cytokine Array | ARY022B | R&D Systems |
| oligomycin | 75351 | Sigma |
| MTP Activity Assay Kit | MAK110-1KT | Sigma |
| Agilent Seahorse XF Cell Mito Stress Test Kit | 103015-100 | Seahorse Bioscience |
| Onetaq | M0480S | New England Biolabs |
| BMP4 | 314-BP | R&D Systems |
| Cholesterol Liquid Stable Reagent kits | TR13421 | ThermoFisher |
| Infinity™ Triglyceride reagent | TR22421 | ThermoFisher |
| TeSR-E7 medium | 05914 | StemCell Technologies |
| TeSR-E8 medium | 05990 | StemCell Technologies |
| ReLeSR | 05872 | StemCell Technologies |
| Y-27632 ROCK inhibitor | 1254 | Tocris |
| TrypLE Express | 12604013 | Gibco (ThermoFisher) |
| RPMI | 11875093 | Gibco (ThermoFisher) |
| ascorbic acid | A4403 | Sigma |
| HepatoZYME | 17705021 | Gibco (ThermoFisher) |
| RNeasy Mini kit | 74104 | Qiagen |
| SuperScript II Reverse Transcriptase kit | 18064014 | Invitrogen (ThermoFisher) |
| random primers | C1181 | Promega |
| dNTP | U1511 | Promega |
| SensiMix SYBR & Fluorescein Kit | QT615-05 | Bioline |
| Qubit RNA BR Assay Kit | Q10210 | Invitrogen (ThermoFisher) |
| Agilent RNA ScreenTape Assay | 5067-5579 | Agilent |
| NEBNext rRNA Depletion Kit | E6350L | New England Biolabs |
| NEBNext Ultra II Directional RNA Library Prep Kit | E7760S | New England Biolabs |
| High Sensitivity D1000 ScreenTape Assay Kit | 5067-5584 | Agilent |

- 1. **Please provide the details of the corresponding methods author for the manuscript:**

| **Nicholas R.F. Hannan,** Division of Cancer and Stem Cells, School of Medicine, University of Nottingham, Nottingham, NG7 2RD, UK.  **Nick.Hannan@nottingham.ac.uk** |
| --- |

**2.0 Please confirm for randomised controlled trials all versions of the clinical protocol are included in the submission. These will be published online as supplementary information.**

| **Not applicable** |
| --- |
