# Supplementary material for: Identification and characterisation of a rare MTTP variant underlying hereditary non-alcoholic fatty liver disease
Source: JHEP Rep. 2023 Apr 23;5(8):100764. doi: 10.1016/j.jhepr.2023.100764 (PMC10362796; doi:10.1016/j.jhepr.2023.100764)
Supplement: Multimedia component 4 [file mmc4.pdf]

Research article

JHEP|Reports

Identification and characterisation of a rare *MTTP* variant underlying hereditary non-alcoholic fatty liver disease

Jane I. Grove,[1,2,†] **Peggy C.K. Lo**,[3,4,†] **Nick Shrine**,[5,†] Julian Barwell,[6] Louise V. Wain,[5,7] Martin D. Tobin,[5,7] Andrew M. Salter,[8] Aditi N. Borkar,[9] Sara Cuevas-Ocaña,[3,4] Neil Bennett,[5] Catherine John,[5] Ioanna Ntalla,[6] Gabriela E. Jones,[6,§] Christopher P. Neal,[10] Mervyn G. Thomas,[11] Helen Kuht,[11] Pankaj Gupta,[12,13] Vishwaraj M. Vemala,[14] Allister Grant,[14,¶] Adeolu B. Adewoye,[15,**] Kotacherry T. Shenoy,[16] Leena K. Balakumaran,[16] Edward J. Hollox,[15,‡] Nicholas R.F. Hannan,[3,4,‡] Guruprasad P. Aithal[1,2,*,‡]

[1]National Institute of Health Research (NIHR) Nottingham Biomedical Research Centre, Nottingham University Hospitals NHS Trust & University of Nottingham, Nottingham, UK; [2]Nottingham Digestive Diseases Centre, Translational Medical Sciences, School of Medicine, University of Nottingham, Nottingham, UK; [3]Translational Medical Sciences, School of Medicine, University of Nottingham, Nottingham, UK; [4]University of Nottingham Biodiscovery Institute, University of Nottingham, Nottingham, UK; [5]Genetic Epidemiology Group, Department of Population Health Sciences, University of Leicester, Leicester, UK; [6]Clinical Genetics Department, University Hospitals Leicester NHS Trust, Leicester, UK; [7]NIHR Leicester Respiratory Biomedical Research Centre, Glenfield Hospital, Leicester, UK; [8]School of Biosciences, University of Nottingham, Nottingham, UK; [9]School of Veterinary Medicine and Science, University of Nottingham, Nottingham, UK; [10]Leicester Cancer Research Centre, University of Leicester, Leicester, UK; [11]Ulverscroft Eye Unit, Department of Neuroscience, Psychology and Behaviour, University of Leicester, Leicester, UK; [12]Department of Chemical Pathology and Metabolic Diseases, University Hospitals of Leicester NHS Trust, Leicester, UK; [13]Department of Cardiovascular Sciences, University of Leicester, Leicester, UK; [14]Department of Gastroenterology, University Hospitals of Leicester NHS Trust, Leicester, UK; [15]Department of Genetics and Genome Biology, University of Leicester, Leicester, UK; [16]Population Health and Research Institute, Trivandrum, India

JHEP Reports **2023.** https://doi.org/10.1016/j.jhepr.2023.100764

Background & Aims: Non-alcoholic fatty liver disease (NAFLD) is a complex trait with an estimated prevalence of 25% globally. We aimed to identify the genetic variant underlying a four-generation family with progressive NAFLD leading to cirrhosis, decompensation, and development of hepatocellular carcinoma in the absence of common risk factors such as obesity and type 2 diabetes.
Methods: Exome sequencing and genome comparisons were used to identify the likely causal variant. We extensively characterised the clinical phenotype and post-prandial metabolic responses of family members with the identified novel variant in comparison with healthy non-carriers and wild-type patients with NAFLD. Variant-expressing hepatocyte-like cells (HLCs) were derived from human-induced pluripotent stem cells generated from homozygous donor skin fibroblasts and restored to wild-type using CRISPR-Cas9. The phenotype was assessed using imaging, targeted RNA analysis, and molecular expression arrays.
Results: We identified a rare causal variant c.1691T>C p.I564T (rs745447480) in *MTTP*, encoding microsomal triglyceride transfer protein (MTP), associated with progressive NAFLD, unrelated to metabolic syndrome and without characteristic features of abetalipoproteinaemia. HLCs derived from a homozygote donor had significantly lower MTP activity and lower lipoprotein ApoB secretion than wild-type cells, while having similar levels of *MTP* mRNA and protein. Cytoplasmic triglyceride accumulation in HLCs triggered endoplasmic reticulum stress, secretion of pro-inflammatory mediators, and production of reactive oxygen species.
Conclusions: We have identified and characterised a rare causal variant in *MTTP*, and homozygosity for *MTTP* p.I564T is associated with progressive NAFLD without any other manifestations of abetalipoproteinaemia. Our findings provide insights into mechanisms driving progressive NAFLD.
Impact and Implications: A rare genetic variant in the gene *MTTP* has been identified as responsible for the development of severe non-alcoholic fatty liver disease in a four-generation family with no typical disease risk factors. A cell line culture created harbouring this variant gene was characterised to understand how this genetic variation leads to a defect in liver cells,

Keywords: Microsomal triglyceride transfer protein; Abetalipoproteinaemia; hiPSC-derived hepatocytes; Lipoprotein ApoB.
Received 31 January 2023; received in revised form 28 March 2023; accepted 11 April 2023; available online 23 April 2023

† These authors share first authorship.
‡ These authors share senior authorship.
¶ Current institution: Treliske Hospital, Truro, Cornwall, UK.
§ Current institution: Department of Genetics, Nottingham University Hospitals NHS Trust, Nottingham, UK.
** Current institution: Department of Biosciences, School of Science and Technology, Nottingham Trent University, Clifton Campus, Nottingham, UK.

* Corresponding author. Address: Nottingham Digestive Diseases Centre, University of Nottingham, Queens Medical Centre Campus, Nottingham NG7 2UH, UK
E-mail address: guru.aithal@nottingham.ac.uk (G.P. Aithal).

ELSEVIER

EASL The Home of Hepatology

which results in accumulation of fat and processes that promote disease. This is now a useful model for studying the disease pathways and to discover new ways to treat common types of fatty liver disease.
© 2023 The Authors. Published by Elsevier B.V. on behalf of European Association for the Study of the Liver (EASL). This is an open access article under the CC BY license (http://creativecommons.org/licenses/by/4.0/).

Introduction

Non-alcoholic fatty liver disease (NAFLD) is a complex trait encompassing a spectrum of accumulation of triglyceride-rich lipid droplets within the hepatocytes (steatosis), non-alcoholic steatohepatitis (NASH; having ballooning degeneration and inflammatory cell infiltration), varying degrees and patterns of fibrosis leading to cirrhosis and its decompensation, and hepatocellular carcinoma (HCC). With rising incidence of obesity and type 2 diabetes, NAFLD is now the most common chronic liver disease, with an estimated 25% population prevalence globally.[1]

Genome-wide association studies (GWAS) have identified a number of genetic risk variants for NAFLD, including *PNPLA3* rs738409 and *TM6SF2* rs58542926, both of which have robust associations with disease phenotypes via functional pathobiological pathways.[2,3] Accretion of the *PNPLA3* variant on lipid droplets sequesters coactivators, resulting in reduced lipolysis and lipophagy, and the *TM6SF2* variant impairs VLDL lipidation. Accumulation of triglycerides in both contexts is associated with progressive liver disease.[2]

Microsomal triglyceride transfer protein (MTP) as a heterodimer with protein disulfide isomerase (PDI) catalyses lipidation and assembly of apolipoprotein B (ApoB)-containing lipoproteins for secretion by hepatocytes, and *MTTP* variants have been linked with susceptibility to NAFLD.[4,5] Rare, loss-of-function mutations in *MTTP* can result in the recessive disorder abetalipoproteinaemia,[6] where MTP deficiency causes defective lipoprotein biosynthesis having multiple severe effects including liver steatosis and fibrosis.[5,7] However, hereditary progressive NAFLD associated with a *MTTP* variant, without any manifestations of abetalipoproteinaemia, has not been previously described.

Here we have clinically characterised a large four-generation family found to have a rare *MTTP* variant located at the interface with PDI resulting in progressive NAFLD, with consequent cirrhosis, liver failure, and HCC in homozygotes. We evaluated post-prandial metabolic responses in carriers of the novel *MTTP* p.I564T variant compared with non-carriers. We used hepatocyte-like cells (HLCs) derived from human-induced pluripotent stem cells (hiPSCs) generated from donor skin fibroblasts from carriers and non-carriers of the *MTTP* variant, as a stable reproducible model for understanding the effect of the variant on the cellular phenotype. This has enabled us to understand how disrupted hepatic lipid homoeostasis can drive steatosis and NAFLD and therefore link genotype to phenotype in hereditary NAFLD.

Patients and methods

Further details of methods used are available in Supplementary information.

Human samples

The clinical studies were approved by the Health Research Authority after review by the National Research Ethics Service: East Midlands Northampton Committee for the Genetics of Rare Inherited Disorders (GRID) study (Ref. 12/EM/0262) and North-East Committee for meal-response study (Ref. 16/NE/0251). Studies were conducted according to the Declaration of Helsinki (Hong Kong Amendment) and Good Clinical Practice (European guidelines). All participants provided written informed consent. For the meal-response analysis, participants were recruited to the study at Queens Medical Centre, Nottingham University Hospitals, between 1 November 2016 and 1 June 2017. Patients with biopsy-proven NAFLD, sex and age matched (within 10 years) to family members, were consecutively identified from a large secondary care cohort who had previously participated in research, and invited to participate. Healthy volunteers were similarly identified and invited. None had diabetes or hazardous alcohol intake and had no known liver disease and had circulating caspase-cleaved CK18 level below 99 U/L.

Clinical investigations followed standard clinical care and included 6-month follow-up as required. Variants segregating with disease were identified following exome sequencing (Illumina HiSeq2000, San Diego, CA, USA). Genotype determination was done using Sanger sequencing (Source Bioscience Ltd, Nottingham, UK) or PCR restriction fragment analysis.

In silico analysis

Models of MTP were based on Protein Data Bank sequence 617S[8] and visualised using Visual Molecular Dynamics software (University of Illinois Urbana-Champaign, Champaign, IL, USA).[9]

Metabolite and protein analyses

Serum cholesterol, triglycerides, and ApoB, and plasma glucose were quantified using calibrated Horiba auto-analyser and reagents following validated standard manufacturer protocols (Horiba ABX, Montpellier, Hérault, France) at the University of Nottingham Metabolic Analysis Facility. Serum insulin was quantified using Human Insulin specific RAI kit (Merck KGaA, Darmstadt, Germany). Plasma lipoproteins were separated by sequential non-equilibrium density-gradient ultracentrifugation.

Apolipoprotein B-100 (ApoB-100) was determined in culture supernatants by ELISA (Merck KGaA, Darmstadt, Germany) in duplicate (twice). MTP activity was determined in lysed cells (in triplicate) at four dilutions using MTP Activity Assay Kit (Merck KGaA). Human NF-κB Pathway, Phospho-Kinase, and XL Cytokine Array Kits (R&D Systems, Minneapolis, Minnesota, USA) were used to determine protein expression or secretion.

Fibroblast reprogramming, hiPSC maintenance, and differentiation

Two 2-mm skin punch biopsies were obtained from study participants, and primary dermal fibroblasts were established and skin fibroblasts were reprogrammed using CytoTune iPS 2.0 Sendai Reprogramming Kit (Invitrogen, Thermo Fisher Scientific, Waltham, MA, USA) in accordance with the manufacturer's guidelines. Mesoderm, ectoderm, and hepatocyte differentiation of hiPSCs was as described previously.[10–12]

CRISPR-Cas9-mediated correction of I564T mutation in the *MTTP*$^{(VAR/VAR)}$

For CRISPR-Cas9 editing, single-guide RNA (gaacatcctgctgtctactg) was cloned and nucleofected (Lonza, Basel, Basel-Stadt,

Switzerland) into the $MTTP^{(VAR/VAR)}$ parental line.[13] Clones were screened to select one with corrected alleles. hiPSCs derived from clones with corrected allele $MTTP^{(WT*/WT*)}$, and the parental line was differentiated to HLCs in parallel for characterisation.

Imaging and analysis of mitochondrial function and cellular reactive oxygen species

Cells were stained using Nile red, Hoechst, DAPI, or antibodies. The mitochondrial content of HLCs was visualised using 100 nM MitoTracker Green FM or MitoTracker Deep Red FM (Invitrogen, Thermo Fisher Scientific, Waltham, MA, USA), and intracellular reactive oxygen species (ROS) and mitochondrial superoxide production was assessed using 2.5 µM CellROX Green or 2.5 µM MitoSox Red. Mitochondrial respiration was determined using the Seahorse XF96 analyser (Seahorse Bioscience, Agilent Technologies Inc., Santa Clara, CA, USA).

Gene expression analysis and RNA sequencing

Quantitative real-time PCR was carried out as described in Supplementary methods. Fold changes in expression were calculated using the comparative ΔΔCt method standardised against the housekeeping gene porphobilinogen deaminase (*PBGD*), and the mean of Ct values ± SE was reported.[12] RNA sequencing and bioinformatics analysis were performed at the Babraham Institute (Cambridge, UK).

Statistical analysis

Statistical analyses were performed using GraphPad Prism version 8 (San Diego, CA, USA) software. One-way ANOVA followed by Dunnett's multiple comparison test were used to compare data from samples grouped by a single factor. Student's *t* test was used to compare the means of variables determined in two groups.

Results

Clinical presentation of family

A British four-generation family with recent Indian ancestry (Fig. 1A) was referred for genetic counselling after three individuals from the same generation developed HCC. Parent A had no history of NAFLD or metabolic syndrome; Parent B presented with NAFLD symptoms aged 80 and was diagnosed with cirrhosis aged 87. Clinical investigations found that all 10 children had NAFLD diagnosis as adults (C–L in Table 1) with progression to NASH, cirrhosis (in seven), and HCC (in four), suggesting a high conversion rate between NAFLD to cirrhosis and NAFLD to HCC. Only one of the affected individuals had a BMI of >30, and instances of type 2 diabetes, hypertension, or hyperlipidaemia within the family were not linked with the presence or severity of disease.

Identification of rare MTTP variant allele associated with diagnosis

Functional variants that were unique to affected family members were identified by whole exome sequencing of 12 affected individuals (Fig. 1A) by comparison with nine unaffected South Asian controls (including spouses of F, G, H, and J; three unrelated participants from the EXCEED study;[14] and two unrelated South Asian individuals with cholangiocarcinoma) and databases of genetic variation identified in the general population. We identified a missense variant: genomic NC_000004.12:g.99608899T>C, NM_000253.2:c.1691T>C, protein NP_000244.2:p.Ile564Thr, in *MTTP* that was unique to affected family members (Fig. 1B and C) and fully segregated with disease phenotype in those individuals analysed. All six homozygous individuals developed cirrhosis, and three also developed HCC, whereas some heterozygotes had no diagnosed disease (Table 1). The presence of both heterozygotes and homozygotes for the rare allele in the third generation of the family implies that individual A must also have carried the variant allele. The presence of fatty liver in wild-type individual I is suggested to be incidental relating to lifestyle factors.

This I564T variant has been previously described in combination with a second rare variant (IVS1+1G>C), manifesting as severe fatty liver in an atypical case of abetalipoproteinaemia in Japan[15] but was reported to have a 'mild effect' in the mother carrying I564T alone. The I564T variant is described in the National Center for Biotechnology Information database[16] as rs745447480 with allele frequency $<1.6 \times 10^5$ in an analysis of 251,024 alleles (gnomAD exomes v2.1.1) present in four non-Finnish European cases. The NCBI allele frequency aggregator population database reports two variants out of a 35,910 global total (both were in a European population). The GEM-Japan whole genome aggregation panel reports one allele in 15,198.[17] Other family members were subsequently tested for this variant, and clinical features of abetalipoproteinaemia[6] were investigated (Tables 1 and 2). Their genotype for other common functional variants at loci in *MTTP* (rs745447480, rs3816873, and rs2306985), *PNPLA3* (rs738409), and *TM6SF2* (rs58542926) associated with NAFLD was also determined. None of 83 patients with NAFLD from the Trivandrum cohort[18] had the *MTTP* p.I564T variant allele.

Mapping of the I564T variant onto the crystal structure[8] located it to the heterodimer interaction interface within a hydrophobic pocket on the MTP subunit surface having surrounding polar and charged residues (Fig. 1C and D). Mutation analysis predicts that medium mutation sensitivity and substitution to threonine, a polar residue, will likely destabilise this hydrophobic pocket, promote interactions with the surrounding polar/charged residues, and thereby cause local conformational variations (PolyPhen = 1, GERP (Genome Evolutionary Rate Profiling) = 5.120, CADD (Combined Annotation-Dependent Depletion) = 20.4, Mutation Assessor = 0.76, and REVEL (Rare Exome Variant Ensemble Learner) = 0.519).[19] A plausible impact on dimer formation and MTP functionality is therefore expected. In contrast, substitutions E98D, N166S, I128T, and H297Q arising from common single-nucleotide polymorphisms (SNPs), map to the protein surface exposed to the solvent in the complex structure (Fig. 1D), and mutation analysis predicts low mutation sensitivity and high tolerance of substitutions at each of these positions.

Post-prandial responses in affected individuals

The phenotypic impact of the *MTTP* p.I564T variant was assessed through investigation of metabolic responses to fat consumption. Family members were invited, and responses in five participants were compared with those in age- and sex-matched healthy volunteers and patients with NAFLD (Fig. 2A and Table S1). The level of ApoB, the protein constituent assembled into chylomicron and VLDL via the activity of MTP, was notably lower in the MTP564-TT homozygote F than in other participants including two MTP564-IT heterozygotes (participants K and Q) and the MTP564-TT liver-transplant recipient J (Fig. 2B). Subsequent testing of six further heterozygotes also showed levels within the normal range. Testing of a stored, pre-transplant serum sample from individual J and clinical data revealed that levels were also markedly lower before receiving a replacement liver where the gene is likely restored.

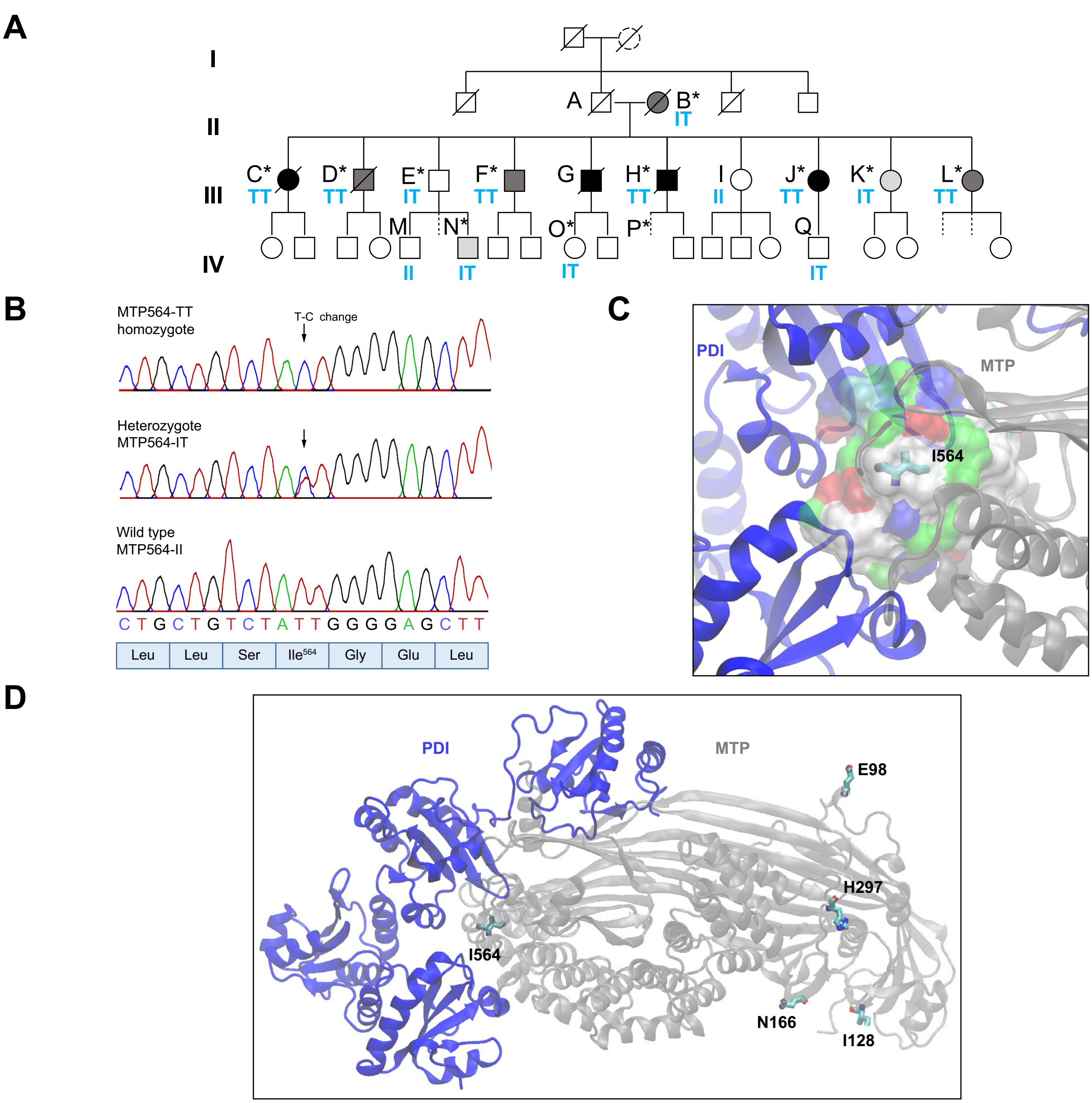

Fig. 1. Identification of a pathogenic variant in a large family with non-alcoholic fatty liver disease. (A) Pedigree. Clinical features are described in Table 1. Diagnosis is indicated by shading: black, hepatocellular carcinoma; dark grey, cirrhosis; light grey, non-alcoholic steatohepatitis. Dashed lines indicate no investigations. *Exome sequenced. Blue letters indicate residue 564 in MTP. (B) Variant rs745447480 sequencing. (C) Local environment of I564 on MTP–PDI interface (hydrophobic pocket [white], polar [green], and charged [red/blue] residues). (D) 564T variant and common non-synonymous variants (side chains: C = cyan; O = red; N = blue) in a model derived from PDB ID:617S,[8] a heterodimer of PDI (blue), and *MTTP* gene product (MTP) (grey). MTP, microsomal triglyceride transfer protein; PDI, protein disulfide isomerase.

We also compared levels of serum ApoB-100, the isoform associated with VLDL, in MTP564-TT homozygotes F and J, with those of wild-type MTP564-II control, participant 1, supporting the proposal that expression of the variant form in the liver results in reduced VLDL secretion (Fig. 2C). Levels of total cholesterol both before and after the meal were also noticeably lower in MTP564-TT homozygote F (Fig. S1) than in all other study participants owing to only very low levels of HDL-cholesterol being present (<0.4 mmol/L). Clinical data confirm this observation (Table 1), and the same phenotype was noted in another MTP564-TT homozygote, H; however, levels in homozygote J, pre-transplant, were normal. Participant F also reported postprandial gastrointestinal discomfort and diarrhoea following the study meal.

The levels of circulating triglyceride in participant F were lower than those in the healthy and disease controls (participants 1 and 2; Fig. 3A) but were similar to levels in participant 3, who possessed two variant alleles for *TM6SF2* rs58542926 (TM6SF2-KK). TM6SF2 is also involved in VLDL secretion and the variant associated with impaired function and decreased serum LDL-cholesterol.[20,21] In contrast, the MTP564-TT homozygote with a liver transplant showed a similar triglyceride response to

Table 1. Clinical characteristics of family members.

Person (Fig. 1A)	*MTTP* p.Ile564Thr	Sex (male/ female)	Liver disease diagnosis (method)	Age diagnosed (years)	Type 2 diabetes	BMI	Hypertension	Liver biochemistry and lipid blood analyses at diagnosis	Other subsequent investigations, treatments, and comorbidities	*PNPLA3* p.I148M	*TM6SF2* p.E167 K	*MTTP* p.I128T	*MTTP* p.Q297H
B	IT	F	Cirrhosis and ascites (USS)	88	x	<30	✓	ALT = 26, AP = 120, Chol = 3.5, TG = 1.29	Cardiovascular-related death aged 90	MM	EK	TT	HH
C	TT	F	HCC and cirrhosis (CT/biopsy)	57	✓	<30	✓	ALT = 38, AP = 129, normal lipids	Liver resection ablation, right hemihepatectomy, HCC recurred; liver-related death	IM	EE	TT	HH
D	TT	M	Cirrhosis (MRI)	60	x	<30	✓	Bilirubin = 312 μmol/L, normal lipids	Liver screen = normal; died of gallbladder sepsis aged 72	IM	EE	TT	HH
E	IT	M	FL (USS)	57	✓	30	✓	ALT = 30, AP = 75, Chol = 2.5, TG = 0.75, HDL = 1.2 mmol/L, LDL = 1.0 mmol/L	Stable for 18 years: TE = 5.8 kPa, CAP = 341, ApoB = 0.94	IM	EE	nd	HH
F	TT	M	Cirrhosis (USS)	56	x	21	✓	ALT = 23, AP = 125, Chol = 2.4, TG = 0.58, HDL = 1.7 mmol/L, LDL = 0.4 mmol/L	No retinitis pigmentosa; vagotomy and pyloroplasty; mild bone marrow failure	IM	EK	TT	HH
G	T*	M	HCC and cirrhosis (biopsy)	57	x	<30	X	Normal lipids	Multifocal HCC: chemotherapy and hepatectomy; died aged 61	nd	Nd	nd	nd
H	TT	M	HCC, cirrhosis, portal hypertension	51	x	<30	✓	Chol = 2.2, TG = 0.2, HDL = 1.6 mmol/L, LDL = 0.5 mmol/L	Liver transplant twice; died of cardiac complications aged 63	IM	EK	TT	nd
I	II	F	FL (USS)	50	x	<30	X		Diet/lifestyle changed: recovered/stable for >5 years: TE = 4.0 kPa; CAP = 223 ApoB = 1.02	IM	EE	TT	HH
J	TT	F	Cirrhosis (MRI) HCC	50 54	x	25	✓	ALT = 52, AP = 87, ApoB = 0.32, normal lipids	Liver transplant age 56: recovered/stable for >5 years: TE = 4.6 kPa, CAP = 214, BMI = 20.3; duodenal biopsy: no evidence of abetalipoproteinaemia	IM	EE	TT	HH
K	IT	F	NASH (USS) (TE:CAP = 380)	48	x	24	✓	ALT = 79, AP = 108, normal lipids, ApoB = 1.0	Diet/lifestyle changed: recovered/stable for 10 years: TE = 6.2 kPa, CAP = 298; duodenal biopsy: no evidence of abetalipoproteinaemia	IM	EK	nd	HH
L	TT	F	Cirrhosis (USS)	59	x	18	✓	Elevated ALT and AST, normal lipids	Stable for 10 years: TE = 10.3 kPa, CAP = 276, ALT = 104, AP = 87, AST = 73, cardiac arrhythmia, osteoporosis, vitamin D deficiency	IM	EE	TT	HH
M	II	M	Healthy	x	x	26	X	Normal lipids	TE = 4.6 kPa, CAP = 253, vitamin D deficiency	II	EK	IT	QH
N	IT	M	NASH (USS)	40	x	28	X	TE = 3.6 kPa, CAP=373; lipids and ALT elevated	Lifestyle and diet modified – stable for 7 years	IM	EK	nd	QH
O	IT	M	FL (USS)	38	x	27	X	Lipids elevated	Lifestyle and diet modified – recovered; stable for 19 years: TE = 5.8 kPa, CAP = 341	II	EK	IT	QH
Q	IT	M	FL (USS)	28	x	19	X	Normal lipids, duodenal Biopsy: evidence of duodenal lipid	Improved after 10 years: TE = 6.3 kPa, CAP = 199	MM	EE	IT	HH

For MTTP p.E98D, all tested were EE; for MTTP p.N166S, all tested were NN.
ALT, alanine transaminase (U/L); AP, alkaline phosphatase (U/L); ApoB, apolipoprotein B (g/L); CAP, controlled attenuation parameter (dB/m); Chol, cholesterol (mmol/L); CT, computed tomography; FL, fatty liver; HCC, hepatocellular carcinoma; MRI, magnetic resonance imaging; nd, not determined; TE, transient elastography; TG, triglycerides (mmol/L); USS, abdominal ultrasonographic steatosis score.
* Likely T carrier based on pedigree.

Table 2. Clinical characteristics of individuals investigated in generation IV.

MTTP genotype p.I564T	Sex (male/ female)	Liver disease diagnosis (method)	Age at testing (years)	Type 2 diabetes	BMI	Hypertension	Liver biochemistry and lipid blood analyses at diagnosis
IT	M	NASH (USS)	32	x	20	X	ALT = 53, AP = 68, Chol = 7, TG = 3.14, VitD <15, TE = 7.4 kPa, CAP = 352
IT	M	FL (USS)	37	x	18	X	Normal LFTs (ALT = 14), normal lipids; HbA1c = 9.5
IT	M	FL (USS)	40	x	28	X	ALT = 74, Chol = 6, TG = 2.12, TE = 3.6 kPa, CAP = 373
IT	F	Healthy (USS)	29	x	20	X	Normal LFTs (ALT = 13), normal lipids; ApoB = 0.95, TE = 3.5 kPa, CAP = 122
IT	M	Healthy (TE)	32	x	27	X	TG = 2.57, Chol = 3.7, ApoB = 1.04, TE = 5.3 kPa
IT	M	Healthy (USS)	31	x	18	X	Normal LFTs (ALT = 19), normal lipids
IT	F	Healthy (USS)	37	x	19	X	Normal LFTs (ALT = 10), Chol = 4.2, TG = 1.77, ApoB = 0.96, TE = 4.2 kPa, CAP = 291
IT	F	Healthy (USS)	39	x	21	x	Normal LFTs (ALT = 23), normal lipids
IT*	F	Healthy (USS)	41	x	20	x	Normal LFTs (ALT = 27), Chol = 4.6, TG = 2.15, ApoB = 1.02, TE = 3.1 kPa, CAP = 231
IT*	M	Healthy (USS)	44	x	<30	x	Normal LFTs (ALT = 27), Chol = 3.5, TG = 2.4; TE = 5.5 kPa, CAP = 281
IT	M	Healthy (USS)	49	x	<30	x	Normal LFTs (ALT = 14), Chol = 4.88, TG = 0.98, ApoB = 1.09, TE = 5.1 kPa, CAP = 245
IT	F	FL (USS)<br>Healthy (USS)	35<br>38	x	21	x	Normal LFTs (ALT = 12), Chol = 6, TG=1.5, ApoB = 1.31, TE = 4.2 kPa, CAP = 325<br>Recovered after 3 years: TE = 6.3 kPa, CAP = 278
IT	M	FL (USS)<br>Healthy (USS)	38<br>42	x	27	x	Normal LFTs (ALT = 39), Chol = 2.9, TG = 0.98<br>Recovered after 4 years: TE = 5.8 kPa, CAP = 243
II*	M	FL (USS)<br>Healthy (USS)	14<br>19	x	21	x	Normal LFTs (ALT = 15), normal lipids<br>Recovered after 5 years: TE = 6.9 kPa, CAP = 216
II*	F	Healthy (USS)	17	x	20	x	Normal LFTs (ALT = 15), normal lipids; TE = 6.4 kPa, CAP = 216
II*	F	Healthy (USS)	20	x	<30	x	Normal LFTs (ALT = 9), normal lipids

ALT, alanine transaminase (U/L); AP, alkaline phosphatase (U/L); ApoB, apolipoprotein B (g/L) (research laboratory data); CAP, controlled attenuation parameter (dB/m); Chol, cholesterol (mmol/L); FL, fatty liver; HbA1c, haemoglobin A1c; LFT, liver function test; NASH, non-alcoholic steatohepatitis; TE, transient elastography; TG, triglycerides (mmol/L); USS, abdominal ultrasonographic steatosis score.
* Deduced from pedigree.

her two matched controls (Fig. 3B). Investigation of VLDL- and chylomicron lipoprotein-associated triglycerides (Fig. 3C–F) revealed that both components were again lowered in participants F (MTP564-TT) and 3 (TM6SF2-KK) compared with the controls, whereas they appeared normal in the MTP564-TT with a transplant, J. Other family members had no notable defects in lipoprotein triglycerides (Fig. S2). Furthermore, VLDL-cholesterol levels were similarly blunted in participants F (MTP564-TT) and 3 (TM6SF2-KK) but not in other family members (Fig. S3), suggesting that transplant hepatocytes and MTP564-IT heterozygote hepatocytes are functioning effectively in VLDL secretion.

Of note, lipoprotein-associated lipid levels are also lower in participant 7, a patient with NAFLD who is homozygous for the *PNPLA3* rs738409 variant (PNPLA3-MM), which has been linked to a relative reduction in large VLDL secretion[22] (Figs. S2A and S3F). Circulating free fatty acids, glucose, and insulin levels in the family members were unremarkable (Fig. S4).

Generation of wild-type and $MTTP^{(VAR/VAR)}$ hiPSCs for disease modelling

To elucidate the mechanisms driving hepatic steatosis in homozygous MTP564-TT patients, we generated hiPSCs and differentiated them into hepatocytes to create an *in vitro* model of the variant. Fibroblasts derived from participant 1, genotyped as MTP564-II (also PNPLA3-148-IM; TM6SF2-167-EE), and family member J, MTP564-TT (also PNPLA3-148-IM; TM6SF2-167-EE), were expanded up to passage 4 and then reprogrammed into hiPSCs: $MTTP^{(WT/WT)}$ and $MTTP^{(VAR/VAR)}$, respectively (Figs. S5–S7). Neither carry the TM6SF2 variant that could confound the observed phenotype. Reprogrammed fibroblasts displayed the typical features of hiPSCs forming dense cell colonies, with well-defined colony boarders, containing cells with a high nuclear-to-cytoplasm ratio. To confirm their pluripotent status, Oct3/4 and NANOG expression was assessed and their ability to differentiate into endoderm, mesoderm, and ectoderm determined. We confirmed the karyotype as normal, without any major chromosomal abnormalities.

MTP levels are lower and lipoprotein secretion is impaired in $MTTP^{(VAR/VAR)}$ HLCs compared with $MTTP^{(WT/WT)}$ HLCs

To prevent bias as a result of differing differentiation efficiency of hiPSC lines, we differentiated cells from study donors 1 and J into HLCs to compare morphology and gene expression profiles.[12] Both cell lines appeared morphologically similar during all stages of differentiation and generated a monolayer of HLCs by Day 21 (Fig. 4A). Gene expression was similar at each of the developmental time points including definitive endoderm, foregut endoderm, and hepatoblast cells. Expression of genes associated with a mature hepatocyte phenotype was not significantly different between the two cell lines (Fig. S7). Analysis of mRNA expression patterns for both cell lines primarily matched 'liver bulk tissue' and then 'hepatocyte' (Table S2), and both showed high similarity to HepG2, HuH7, and Hep3B cell lines.

Nile red and Oil Red O staining revealed phenotypical differences with apparent significant sequestration of lipid via development of discrete lipid droplets and microvesicular steatosis throughout the cytoplasm in the $MTTP^{(VAR/VAR)}$ HLCs after 48 h of culture (Fig. 4B and C). Quantification showed levels were more than fourfold higher in $MTTP^{(VAR/VAR)}$ HLCs (Fig. 4E), consistent with the proposed reduced VLDL secretion in cells expressing MTP564-TT, restricting removal of intracellular triglycerides.

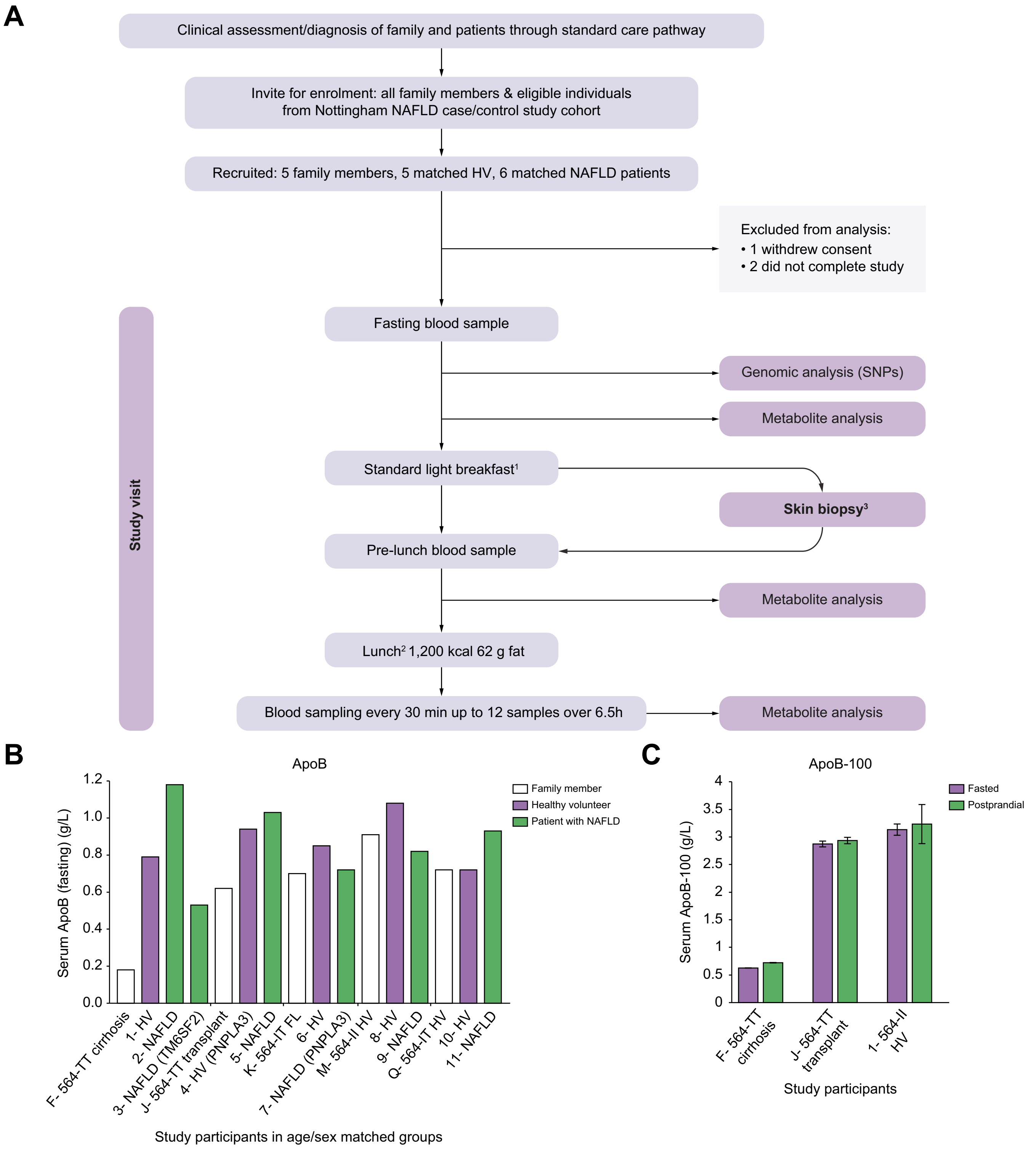

Fig. 2. Meal-response study to investigate metabolism in family members and matched controls. (A) Study design. (B) ApoB levels. Participants are grouped according to age and sex matching to family members F, J, K, Q, and M (Table S1). MTP residue 564 is indicated (TT/TI/II). *PNPLA3* or *TM6SF2* in parentheses indicates individuals homozygous for variant rs738409 or rs58542926, respectively. (C) Serum ApoB-100 (mean levels ± standard deviation). [1]Milk and cornflakes. [2]80–175 min between breakfast start and pre-lunch sample. [3]From participants J and 1 used to derive cell lines. ApoB, apolipoprotein B; ApoB-100, apolipoprotein B-100; HV, healthy volunteers; MTP, microsomal triglyceride transfer protein; NAFLD, non-alcoholic fatty liver disease; SNP, single-nucleotide polymorphism.

Importantly, however, although both immunocytochemistry and mRNA expression analysis suggested a trend towards lower MTP levels compared with cells expressing the wild-type allele, this was not statistically significant (Fig. 4D, F, and G). To assess the VLDL export capabilities of the cell lines, and thus functioning of variant MTP in lipoprotein biosynthesis, levels of

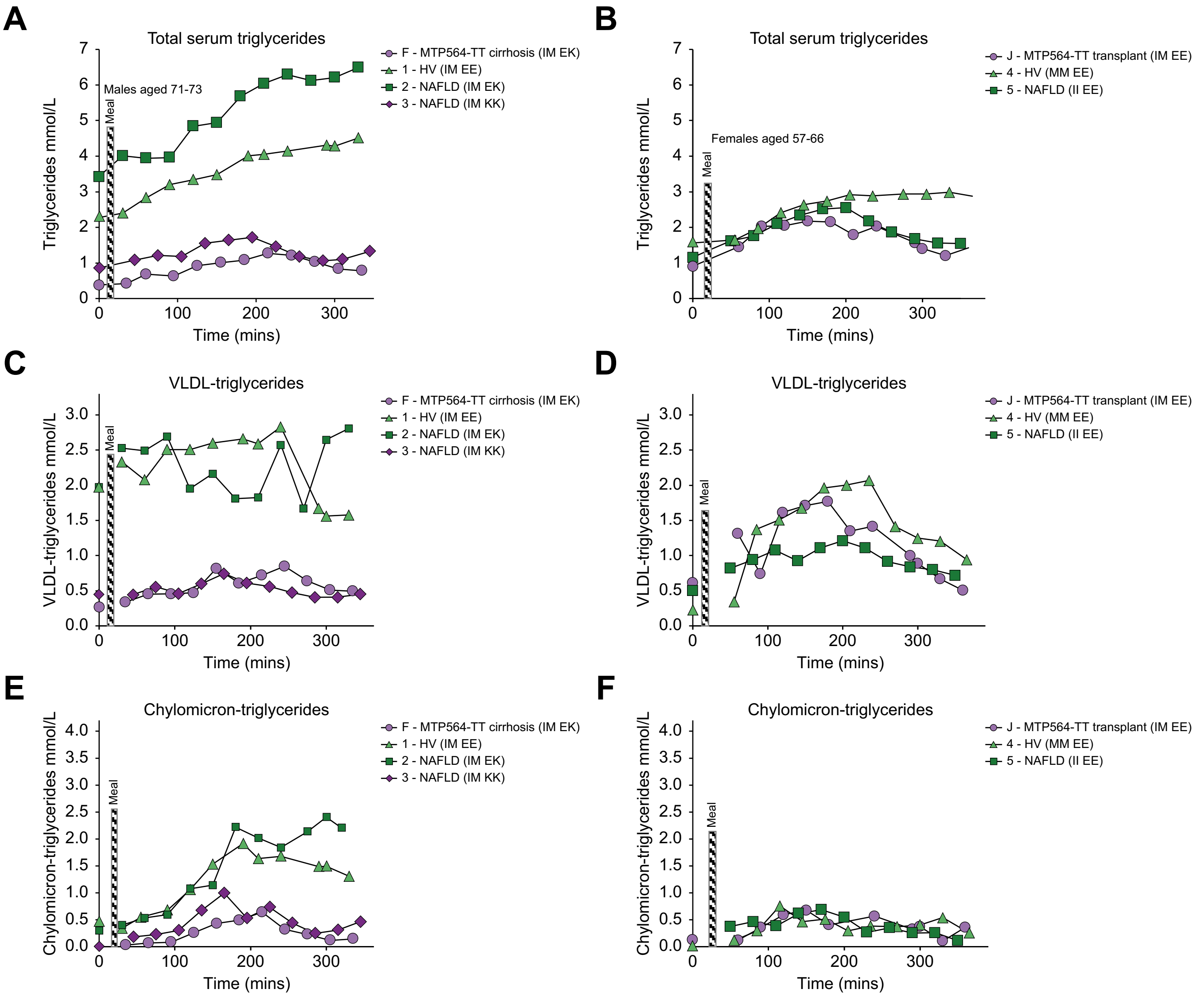

Fig. 3. Triglyceride levels in study participants. Total serum triglycerides: (A) Participant F and matched controls and (B) participant J and matched controls. VLDL-triglyceride: (C) Participant F and matched controls and (D) participant J and matched controls. Chylomicron-triglyceride: (E) Participant F and matched controls and (F) participant J and matched controls. *MTTP* genotypes are shown for family members. *PNPLA3* p.I148M and *TM6SF2* p.E167 K genotypes are indicated in parentheses. HV, healthy volunteers; NAFLD, non-alcoholic fatty liver disease.

secreted ApoB-100 were determined. There was significantly less, but detectable, ApoB-100 in the media from $MTTP^{(VAR/VAR)}$ compared with $MTTP^{(WT/WT)}$ HLCs (Fig. 4H), confirming the clinical phenotype and supporting the suggestion that the MTP variant in these patients affects lipid trafficking.

Increased generation of ROS and altered mitochondrial respiration in $MTTP^{(VAR/VAR)}$ HLCs

Hepatic free fatty acids can be converted to triglyceride for storage as cytoplasmic droplets or secreted as VLDL, or else directly metabolised via mitochondrial β-oxidation. Therefore, impaired MTP functionality restricting lipid secretion, thus increasing the availability of fatty acids, may impact on mitochondrial activities. Using mitochondrial stress testing measuring the oxygen consumption rate in live cells revealed that $MTTP^{(VAR/VAR)}$ HLCs had significantly higher basal and maximal mitochondrial respiration than mitochondria from the wild-type cell line (Fig. 4I). Of importance, increased β-oxidation would generate additional ROS, which can be a major driver of oxidative stress and cellular dysfunction. Both mitochondrial superoxide production and cytoplasmic ROS were significantly higher in the $MTTP^{(VAR/VAR)}$ HLCs than in $MTTP^{(WT/WT)}$ HLCs (Fig. 4J and K and Fig. S8), consistent with increased fatty acid metabolism.

Increased NF-κB signalling, inflammation, ER stress, and secretion of pro-inflammatory mediators in $MTTP^{(VAR/VAR)}$ HLCs

Impaired lipid trafficking and lipoprotein assembly incurred as a consequence of reduced MTP functionality is likely to cause a range of cellular responses including endoplasmic reticulum (ER) stress and inflammation. Analysis of mRNA revealed that expression of ER stress mediators spliced X-box binding protein-1 (SxBP1), activating transcription factor 6 (ATF6), and binding immunoglobulin protein (BIP), and the ER stress transducer inositol requiring enzyme 1 (IRE1) were significantly higher in $MTTP^{(VAR/VAR)}$ HLCs (Fig. 5A).

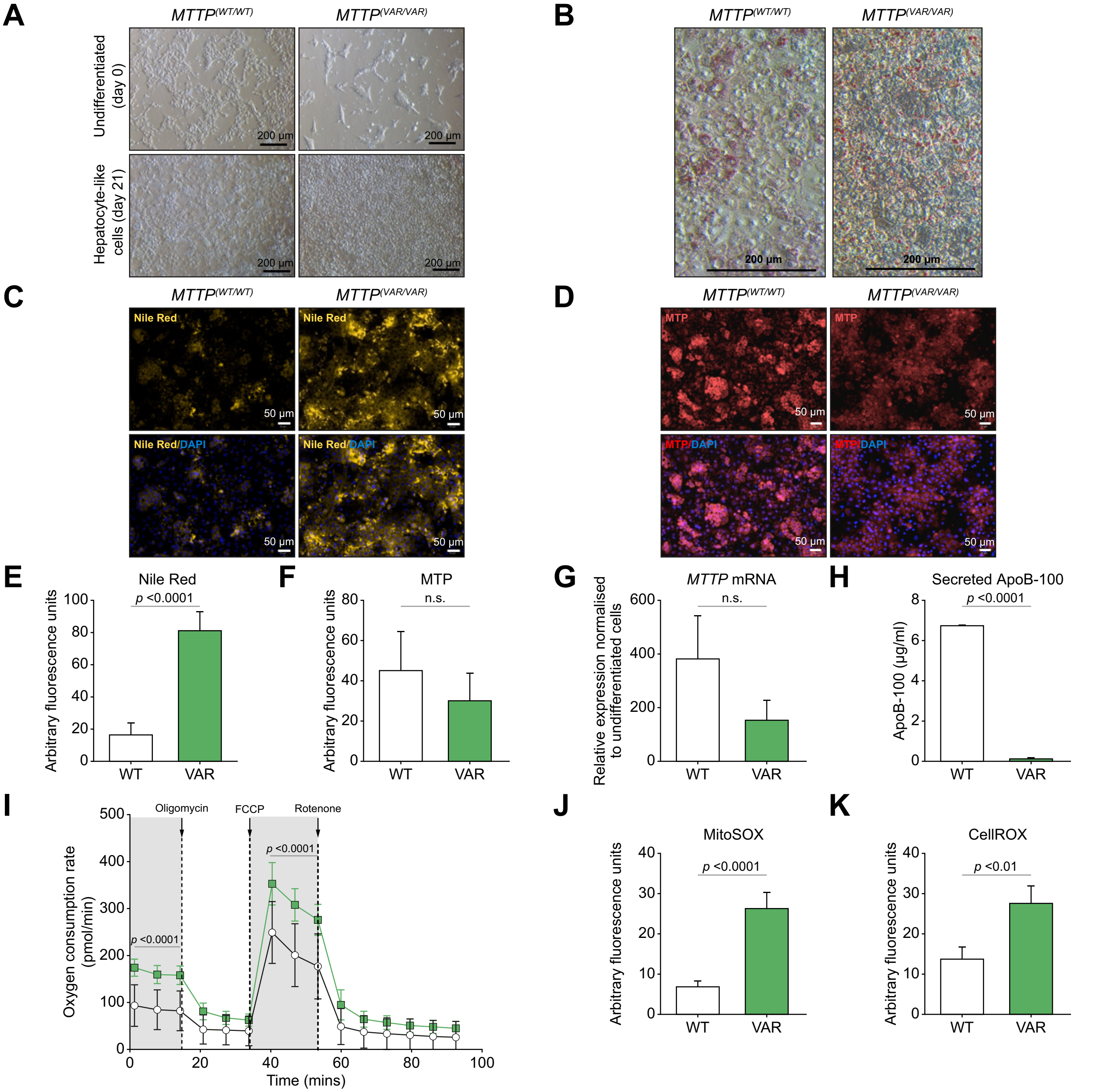

Fig. 4. Characterisation of MTP-564T homozygote variant, *MTTP*$^{(VAR/VAR)}$, and wild-type HLCs. (A) Light microscopy image of terminally differentiated hiPSC-derived HLCs. (B) Oil Red O staining of HLCs (light microscopy). (C) Nile red staining of lipids ± DAPI staining (fluorescence microscopy). (D) MTP expression immunocytochemistry ± DAPI staining. Quantification of Nile red fluorescence (E) and MTP staining (F) in HLCs. (G) Expression of MTP determined by quantitaive PCR. (H) ApoB-100 secretion by HLCs (ELISA). (I) Basal and maximal mitochondrial respiratory rates in *MTTP*$^{(WT/WT)}$ (white circles) and *MTTP*$^{(VAR/VAR)}$ (green squares) HLCs. Quantification of cellular superoxide (J) and reactive oxygen species (K) from fluorescence microscopy. Mean ± SE. p <0.05 is significant (t test, paired, two-tailed). ApoB-100, apolipoprotein B-100; hiPSC, human induced pluripotent stem cell; HLC, hepatocyte-like cell; MTP, microsomal triglyceride transfer protein.

MTTP$^{(VAR/VAR)}$ HLCs expressed significantly higher levels of NF-κB (Fig. 5B), suggesting greater ER stress and active pro-inflammatory response.

These observations were confirmed by assessing changes in intracellular and extracellular signalling (Fig. 5C–E). The observed lipid accumulation in the novel MTP564-TT variant strain was associated with increases in NF-κB pathway components, indicative of activation, and phosphorylation of pro-inflammatory and pro-apoptotic pathway mediators including RelA/p65 complex, apoptosis-associated speck-like protein containing a caspase-recruitment domain (ASC), p53, Fas-associated death domain (FADD), and CD95. This increased pro-inflammatory signalling coincided with increased secretion of pro-inflammatory mediators including chemokine (C-C motif)

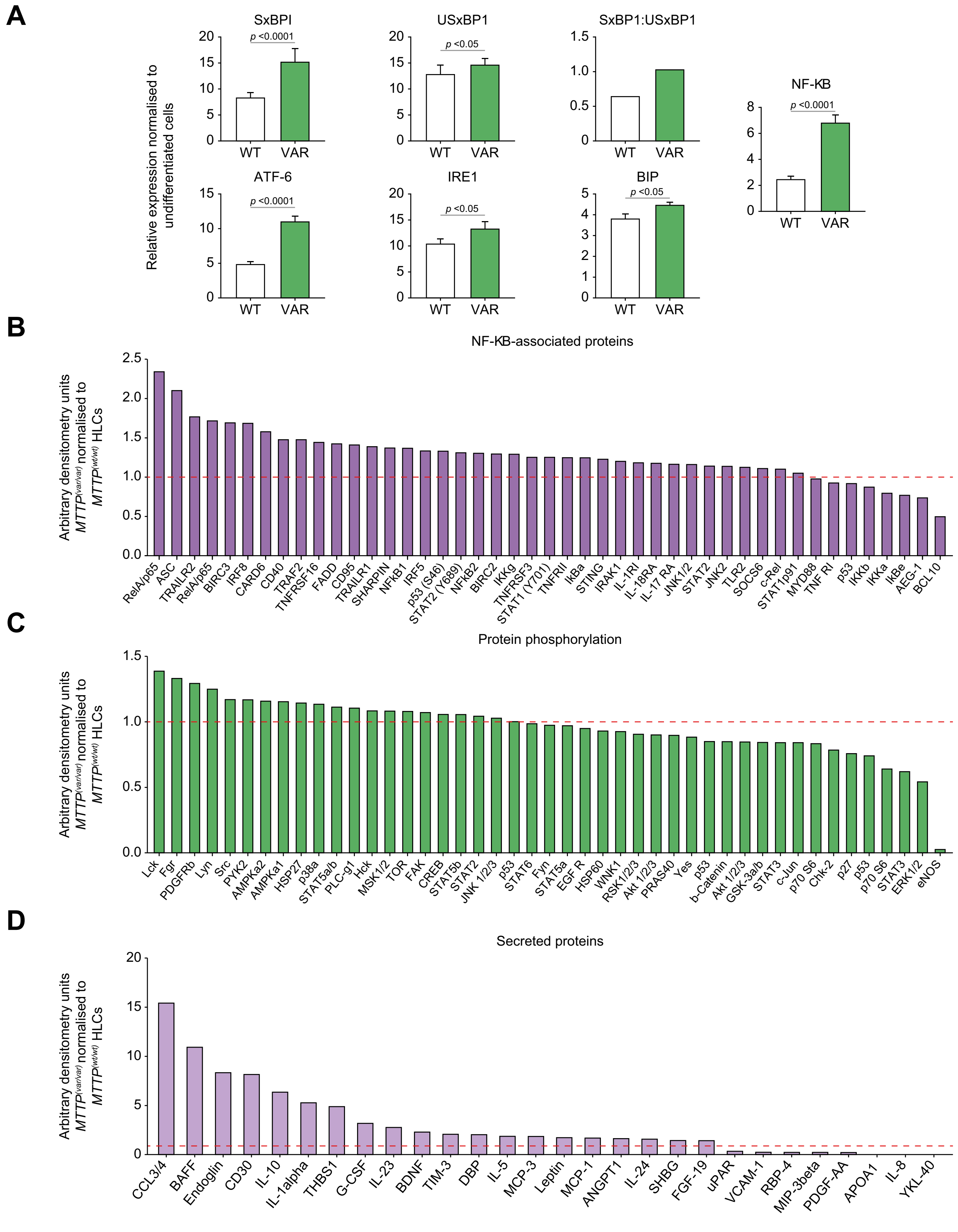

Fig. 5. Phenotypic characterisation of *MTTP*$^{(VAR/VAR)}$ hiPSC-derived HLCs. (A) Expression of inflammation-related and ER stress-related genes. Mean ± SE. p <0.05 is significant (t test, paired, two-tailed) (B) Expression of NF-κB-associated intracellular signalling proteins in *MTTP*$^{(VAR/VAR)}$ HLCs, relative to expression in *MTTP*$^{(WT/WT)}$. (C) Phosphorylated proteins in *MTTP*$^{(VAR/VAR)}$ HLCs, normalised to *MTTP*$^{(WT/WT)}$. (D) Secreted proteins from *MTTP*$^{(VAR/VAR)}$ HLCs, normalised to *MTTP*$^{(WT/WT)}$. ATF6, activating transcription factor 6; BIP, binding immunoglobulin protein; ER, endoplasmic reticulum; hiPSC, human induced pluripotent stem cell; HLC, hepatocyte-like cell; IRE1, inositol requiring enzyme 1; SxBP1, spliced X-box binding protein-1; USxBP1, unspliced X-box binding protein-1.

ligand 3/4 (CCL3/4), B cell–activating factor of the TNF family (BAFF), CD30, IL-1, IL-10, IL-23, D-box binding PAR BZIP transcription factor (DBP), and leptin while showing decreased retinol binding protein 4 (RBP4) and platelet-derived growth factor (PDGF). Notably, *MTTP*$^{(VAR/VAR)}$ HLCs had more than 10-fold lower expression of phosphorylated endothelial nitric oxide synthase (eNOS) than *MTTP*$^{(WT/WT)}$ HLCs. There were no significant differences in protein phosphorylation in the other 44 proteins assayed. However, there was a trend towards decreased extracellular signal-regulated kinase 1/2 (ERK1/2) phosphorylation in *MTTP*$^{(VAR/VAR)}$ HLCs.

MTTP$^{(VAR/VAR)}$ HLCs show increased expression of ECM remodelling and lipid metabolising genes

Bioinformatics analysis of mRNA-sequencing data to assess genome-wide changes in gene expression revealed 472 genes differentially expressed (>1 × $\log_2$-fold) between *MTTP*$^{(VAR/VAR)}$ and *MTTP*$^{(WT/WT)}$ cultured HLCs. These fulfil diverse cellular functions including glycolysis, lipid oxidation, oxidative phosphorylation, and complement activation (Fig. S9) and support previous observations of increased ROS generation and altered mitochondrial activity. Gene Ontology terms associated with the changing genes (Tables S3–S5) were mostly implicated in extracellular matrix (ECM) remodelling, ECM organisation and degradation, ECM–receptor interactions, and proteoglycan modifications, suggesting that ECM remodelling may be initiated during hepatosteatosis.

Confirmation that the homozygous rs745447480 variant in the *MTTP*$^{(VAR/VAR)}$ HLCs results in significantly lower MTP lipid transfer activity

A third cell line, *MTTP*$^{(WT*/WT*)}$, was generated from *MTTP*$^{(VAR/VAR)}$ in which the MTP564-TT in was gene-edited to wild-type 564-II using CRISPR-Cas9 transfection and selection of a corrected cloned (Fig. S10). The resultant differentiated cell line displayed the same characteristics as the *MTTP*$^{(WT/WT)}$ cell line (Fig. 6 and Fig. S11). This enables us to rule out the possibility of other genetic variants harboured by the patient or healthy volunteer influencing the observed *in vitro* phenotype. MTP lipid transfer activity of the original patient-derived HLCs, *MTTP*$^{(VAR/VAR)}$, was compared with that of the gene-edited HLCs to establish the impact of the SNP rs745447480. For equivalent cellular protein quantity, MTP activity was significantly lower in the *MTTP*$^{(VAR/VAR)}$ HLCs than in the edited derivative *MTTP*$^{(WT*/WT*)}$, having only 61% of the level determined in the wild-type cells. This is distinct from other described variants that abolish MTP activity and may explain the observed apparent phenotype of impaired lipid trafficking. To assure that the observed phenotype does not reflect compensatory activity of TM6SF2 in the same pathway, *TM6SF2* mRNA expression levels were determined. This indicated that TM6SF2 expression, normalised to undifferentiated cells, is not higher in *MTTP*$^{(VAR/VAR)}$ than in *MTTP*$^{(WT*/WT*)}$ and therefore suggests that the observed phenotype is not caused by increased TM6SF2 (Fig. S12).

Discussion

We have identified and characterised a rare *MTTP* variant (p.I564T) as causative for the Mendelian trait associated with an inherited form of NAFLD in a four-generation family. Our investigation has revealed a variant resulting in decreased ApoB-containing lipoprotein secretion in homozygotes (but not heterozygotes), in contrast to other variants causing abetalipoproteinaemia, where ApoB is undetectable (Fig. 2 and Table 1).[6,7,23–25] Although other carriages of this variant have been reported, no phenotypic characteristics related to these are previously described.[15] None of the GWAS so far, including that using UK Biobank[26] and the largest cross-ancestry GWAS,[27] have identified this particular *MTTP* variant (p.I564T) in association with NAFLD. Protein modelling (Fig. 1C) suggests that the substitution moderately affects the protein structure and likely impacts upon the interaction with PDI in the formation of a normal heterodimeric enzyme but is unlikely to abolish all functionality as in abetalipoproteinaemia. Presentation of homozygote cases is clearly distinct from abetalipoproteinaemia,[23,28] supporting the suggested subtle phenotype whereby impact is limited to liver lipid imbalance. This provides potential for treatment through reduced dietary fat intake and makes it an attractive model for cellular consequences of lipid accumulation.

Our phenotyping studies demonstrated distinct VLDL secretion responses following meal challenge and ApoB levels in the two *MTTP*-564TT family members: although these biomarkers were substantially low in untreated individual F, these were in the normal range in liver transplant recipient J (Fig. 2B and Table 1). Previously, the *MTTP* -493 variant (rs1800591) G allele linked with reduced MTP function has been associated with NAFLD susceptibility in a meta-analysis of 11 case–control studies.[29] Moreover, an association study in patients without diabetes with NASH found that GG homozygotes had significantly higher plasma triglycerides, intestinal and hepatic large VLDL, and oxidised LDL than the GG/GT group.[30] All of the five

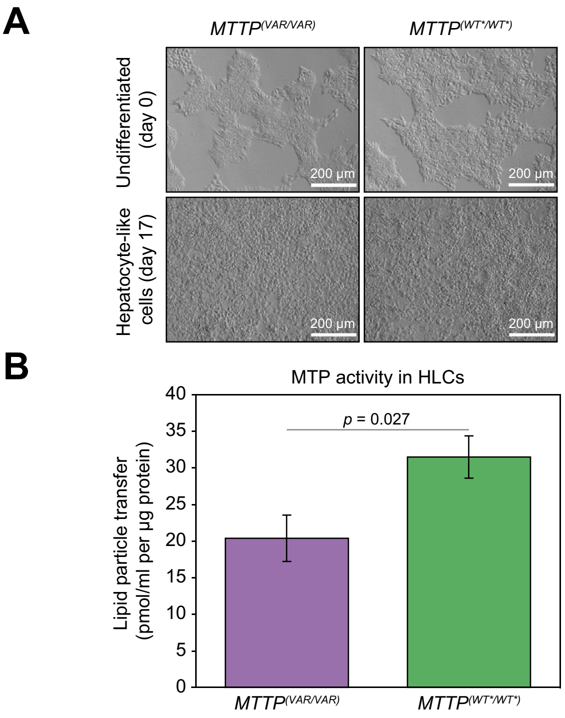

Fig. 6. Restoration of activities by gene editing of *MTTP*$^{VAR/VAR}$ 564-TT to 564-II. (A) Light microscopy showing terminally differentiated hiPSC-derived HLCs from *MTTP*$^{(VAR/VAR)}$ and gene-edited derivative *MTTP*$^{(WT*/WT*)}$. (B) MTP enzyme activity in HLCs. Mean ± SE; significance level p <0.05 (t test). hiPSC, human induced pluripotent stem cell; HLC, hepatocyte-like cell; MTP, microsomal triglyceride transfer protein.

MTP564-IT heterozygous individuals showed ApoB and lipoprotein levels within the normal range, consistent with reports that a single copy of *MTTP* is sufficient.[31]

Functional analysis of a childhood case with compound heterozygosity for *MTTP* c.619-5_619-2del and p.L435H, having severe liver fibrosis but no typical abetaliporoteinaemia symptoms, found that p.L435H abolished MTP activity, whereas the intronic variant resulted in 26% of transcripts being normally spliced, allowing limited MTP expression.[32] This suggested that the residual expression and resulting MTP activity was sufficient for substantial biological activity covering majority of necessary functionality, except for liver functions. Consistent with this, our observed modest reduction in activity to 61% would thus be predicted to have a subtle effect only on liver homoeostasis. The modest impact on protein function *in vitro* is compatible with the less severe, non-abetalipoproteinaemia clinical phenotypes described. The observed 'normal' levels of expression in HLCs may suggest that the I564M change effects translation, protein stability/turnover, or enzymatic function. Our *in silico* models suggested that the impact is on heterodimer assembly, stability, interaction, or activity. An impact on activity would be entirely compatible with the unchanged protein levels observed in HLCs.

Disruption of ApoB biosynthesis and associated VLDL secretion has been widely described with a spectrum of consequences linked to characterised pathologies.[33] ApoB missense variants are also associated with development of fibrosis and HCC linked to NAFLD.[34] Furthermore, rare variants in *MTTP* were found to be associated with increased hepatic fat in the UK Biobank cohort.[5] The underlying mechanisms are inherently linked to nutritional intake with diets high in fats (increasing hepatic fat content) and carbohydrate (increasing hepatic *de novo* lipogenesis), resulting in hyperlipidaemia. Hepatic lipid balance is dependent on secretion of VLDL, which is limited by availability/activity of MTP, so any variants with altered activities are likely to have metabolic effects. The low frequency of the *MTTP* p.I564T variant reported in large datasets[16] means that identification, recruitment, and analysis of further carriers to strengthen the study would be very difficult.

Overall, VLDL secretion may increase with hepatic steatosis related to metabolic syndrome.[35] However, decreased VLDL secretion has been reported in carriers of PNPLA3 G allele.[22] VLDL secretion is also lowered in *TM6SF2* T carriers,[20,21,36] which affects the same pathway. We considered key genetic risk factors, *PNPLA3* and *TM6SF2* variants, likely to influence disease phenotype as polygenic scores have been proposed for NAFLD.[37] *TM6SF2* p.E167K is of particular interest because it acts in the same pathway as MTP, so similarities in phenotypes would be expected. In our study, post-prandial secretion of VLDL, the predominant post-prandial lipoprotein associated with hyperlipidaemia,[38] was lower in participants homozygous for *TM6SF2* or *PNPLA3*, but fasting ApoB levels were normal. By contrast, in *MTTP* p.I564T homozygotes, we observed a reduced level of both circulating ApoB and VLDL-associated lipids. We have specifically considered the possibility of functional redundancy between TM6SF2 and MTP and potential additive effects of the variants *in vivo* and *in vitro*. First, the clinical characterisation in Table 1 shows that the disease phenotype is not linked to the *TM6SF2* carriage as three affected siblings do not carry the *TM6SF2* variant. Second, the post-prandial lipid data are consistent with reduction in circulating lipids owing to the lack of either wild-type *MTTP* or wild-type *TM6SF2*, whereas heterozygotes retain functionality.

In addition to demonstrating the functional consequences of the *MTTP* p.I564T variant, the HLCs derived from hiPSCs provide a disease model for early-stage NAFLD, beyond triglyceride accumulation. As the donors are wild type for *TM6SF2*, the cell phenotype reflects only the impact of the *MTTP* variant. Studies have shown a link between the amount of steatosis, fibrosis development, and liver disease mortality,[39] with lipid metabolism acting as the initiator of progression to NASH.[40] Although triglyceride sequestration may be protective, when fatty acid storage and disposal routes reach capacity, alternative pathways resulting in lipotoxicity can occur. Components of these pathways, such as acetyl-CoA carboxylase-1/2 (ACC-1/2), farnesoid X-activated receptor (FXR), fibroblast growth factor-19 (FGF19), and stearoyl-Coenzyme A desaturase-1 (SCD-1), are thus being tested as therapeutic targets.[41] Increased mitochondrial fatty acid β-oxidation may provide a protective response but uncontrolled results in the generation of ROS, which can be a major driver of oxidative stress and cellular dysfunction (Figs. 4I and 6).

We show that as lipid accumulation increases, hepatocytes have increased ER stress; activate pro-inflammatory signalling pathways including NF-κB, P53, and eNOS; and secrete pro-inflammatory mediators. This coincides with increased production of ROS, superoxide production, and alterations to mitochondrial respiration driving the disease progression leading to cirrhosis and HCC, as seen among the family members. Similar findings were reported in cardiomyocytes derived in an *MTTP* pR46G variant model.[42] Excessive lipid accumulation in hepatocytes can serve as substrates for the generation of lipotoxic species. One of the major consequences of hepatic lipid metabolism is mitochondrial β-oxidation and esterification to form triglycerides, which can serve as a protective mechanism against lipotoxicity in hepatocytes. However, if lipid accumulation is in excess of the β-oxidation capacity, such as in NAFLD, toxic intermediates can accumulate, which induce metabolic stress and subsequent inflammation and cell death. Changes in expression of ECM remodelling-associated genes, suggestive of ECM remodelling occuring during steatosis, may contribute to drive progression to fibrosis, which is clinically observed later.

We conclude that the main feature of the *MTTP* p.I564T variant is impaired ApoB secretion and hepatic lipid accumulation as a result of decreased lipid transfer activity distinct from the classical abetalipoproteinaemia phenotype where MTP expression is abolished. Identification and characterisation of a rare disease such as hereditary NAFLD is of medical significance in Indian populations where high rates of founder events have been reported.[43] In addition, HLC modelling supports this, providing additional details of signalling, inflammatory, and metabolic cellular pathways involved, highlighting pathophysiology driving NAFLD progression and possible therapeutic targets.

Abbreviations

ALT, alanine transaminase; AP, alkaline phosphatase; ApoB-100, apolipoprotein B-100; ApoB, apolipoprotein B; BIP, binding immunoglobulin protein; CAP, controlled attenuation parameter; Chol, cholesterol; CT, computed tomography; ECM, extracellular matrix; eNOS, endothelial nitric oxide synthase; ER, endoplasmic reticulum; ERK1/2, extracellular signal-regulated kinase 1/2; FL, fatty liver; GRID, Genetics of Rare Inherited Disorders; GWAS, genome-wide association studies; HbA1c,

haemoglobin A1c; HCC, hepatocellular carcinoma; hiPSC, human induced pluripotent stem cell; HLC, hepatocyte-like cell; IRE1, inositol requiring enzyme 1; LFT, liver function test; MRI, magnetic resonance imaging; MTP, microsomal triglyceride transfer protein; NAFLD, non-alcoholic fatty liver disease; NASH, non-alcoholic steatohepatitis; nd, not determined; PDI, protein disulfide isomerase; ROS, reactive oxygen species; SE, standard error; SNP, single-nucleotide polymorphism; SxBP1, spliced X-box binding protein-1; TE, transient elastography; TG, triglycerides; USS, abdominal ultrasonographic steatosis score.

Financial support

This work was supported by the Medical Research Council (MRC) Nottingham Molecular Pathology Node (grant number MR/N005953/1), National Institute of Health Research (NIHR) Nottingham Digestive Diseases Biomedical Research Unit, and Nottingham Biomedical Research Centre (BRC-1215-20003). JIG and GPA are supported by NIHR Nottingham Biomedical Research Centre. KTS and LKB are supported by Population Health and Research Institute. All cell modelling was supported by the RoseTrees Trust and the Stoneygate Trust (M546). NRFH and SCO are supported by the Medical Research Council (MR/S009930/1). LVW holds a GSK/British Lung Foundation Chair in Respiratory Research (C17-1). The research was supported by the NIHR Leicester Biomedical Research Centre; CJ held a Medical Research Council Clinical Research Training Fellowship (MR/P00167X/1). EXCEED is supported by the University of Leicester, the NIHR Leicester Respiratory Biomedical Research Centre; by Wellcome (202849); and by Cohort Access fees from studies funded by the Medical Research Council (MRC), BBRSC, NIHR, the UK Space Agency, and GSK. This work is supported by BREATHE – The Health Data Research Hub for Respiratory Health (UKR_PC_19004) in partnership with SAIL Databank. The exome sequencing was funded by MRC Grant Senior Clinical Fellowship to MDT (G0902313), and we thank the high-throughput genomics group at the Wellcome Trust Centre for Human Genetics (funded by Wellcome Trust Grant 090532/Z/09/Z and MRC Hub Grant G090074791070) for the generation of the sequence data. MDT is supported by a Wellcome Trust Investigator Award (WT202849/Z/16/Z) and holds an NIHR Senior Investigator Award. The funders had no role in study design, data collection and analysis, decision to publish, or preparation of the manuscript.

Conflicts of interest

GPA has served as a consultant and an advisory board member for Pfizer Inc, Inventiva Pharma, GlaxoSmithKline, and KaNDy Therapeutics; he has been a consultant to Servier, Clinipace, Albireo Pharma, BenevolentAI Bio, DNDi, BerGenBio ASA, Median Technologies, FRACTYL, Amryt Pharma, and AstraZeneca; and has given presentations on behalf of Roche Diagnostics and Medscape. IN is employed by Gilead Sciences Ltd. (since August 2019). All other authors declare no conflict of interests.

Please refer to the accompanying ICMJE disclosure forms for further details.

Authors' contributions

Conceptualisation: GPA . Study design: GPA, JB, NRFH, AMS, LVW, MDT. Data curation: JIG, PCKL, NRFH, EJH, NS, NB, CJ, IN. Funding: GPA, NRFH, EJH, KTS, MDT, LVW. Formal analysis: JIG, NRFH, PCKL, NS, NB. Investigation: JIG, PCKL, GPA, NRFH, JB, NS, ANB, SCO, NB, EJH, AG, GEJ, MGT, HK, ABA, PG, VMV. Supervision: GPA, NRFH, JB, AMS, JIG, LVW, MDT . Resources: GPA, JIG, NRFH, AMS, JB, CJ, IN, CPN, KTS, LKB. Interpretation of data: GPA, JIG, NRFH, AMS, MDT, JB, LVW, EJH. Writing – original draft: JIG, PCKL, NRFH, AMS, JB, LVW, GPA . Writing – review and editing: all authors.

Data availability statement

Study data are available on request. The three EXCEED exome sequences are available in the European Genome-phenome Archive using accession number EGAD00001007649. Access to sensitive genetic data and cell lines will be restricted to research facilities with institutional data and material transfer agreements to protect participant anonymity.

Acknowledgements

The views expressed are those of the authors and not necessarily those of the National Health Service (NHS), the NIHR, or the Department of Health. We thank all the research participants, particularly the family involved. We are grateful to the study teams of the EXCEED study, Trivandrum cohort, and NASH study for their contributions. We are grateful to the clinical team at University Hospitals of Leicester NHS Trust for clinical workup and acknowledge support from the late Roger Williams. We thank the high-throughput genomics group at the Wellcome Trust Centre for Human Genetics for the generation of the sequence data. We thank Sally Cordon and Ian Macdonald for assistance with metabolic analysis, and Melanie Lingaya and Calum Greenhalgh for technical support. We thank Ester Burden-Teh and Jane Chalmers for assistance with taking skin biopsies and Antonella Ghezzi for obtaining clinical samples for genotyping. We thank Beth Robinson and the Nottingham Digestive Diseases Team for assistance with coordinating participant involvement and the meal study. We also thank all participants and staff who have contributed their time to the study.

Supplementary data

Supplementary data to this article can be found online at https://doi.org/10.1016/j.jhepr.2023.100764.

References

Author names in bold indicate shared co-first authorship.

[1] Younossi ZM, Koenig AB, Abdelatif D, Fazel Y, Henry L, Wymer M. Global epidemiology of nonalcoholic fatty liver disease-Meta-analytic assessment of prevalence, incidence, and outcomes. Hepatology 2016;64:73–84.

[2] Krawczyk M, Liebe R, Lammert F. Toward genetic prediction of nonalcoholic fatty liver disease trajectories: *PNPLA3* and beyond. Gastroenterology 2020;158:1865–1880.e1861.

[3] Trépo E, Valenti L. Update on NAFLD genetics: from new variants to the clinic. J Hepatol 2020;72:1196–1209.

[4] **Tan J**, Zhang J, **Zhao Z**, Zhang J, Dong M, Ma X, et al. The association between SNPs rs1800591 and rs3816873 of the MTTP gene and nonalcoholic fatty liver disease: a meta-analysis. Saudi J Gastroenterol 2020;26:171–178.

[5] **Haas ME**, **Pirruccello JP**, **Friedman SN**, Wang M, Emdin CA, Ajmera VH, et al. Machine learning enables new insights into genetic contributions to liver fat accumulation. Cell Genom 2021;1:100066.

[6] Shoulders CC, Brett DJ, Bayliss JD, Narcisi TM, Jarmuz A, Grantham TT, et al. Abetalipoproteinemia is caused by defects of the gene encoding the 97 kDa subunit of a microsomal triglyceride transfer protein. Hum Mol Genet 1993;2:2109–2116.

[7] Di Filippo M, Moulin P, Roy P, Samson-Bouma ME, Collardeau-Frachon S, Chebel-Dumont S, et al. Homozygous MTTP and APOB mutations may lead to hepatic steatosis and fibrosis despite metabolic differences in congenital hypocholesterolemia. J Hepatol 2014;61:891–902.

[8] Biterova EI, Isupov MN, Keegan RM, Lebedev AA, Sohail AA, Liaqat I, et al. The crystal structure of human microsomal triglyceride transfer protein. Proc Natl Acad Sci U S A 2019;116:17251.

[9] Humphrey W, Dalke A, Schulten K. VMD: visual molecular dynamics. J Mol Graph 1996;14:33–38. 27–38.

[10] **Bertero A**, **Pawlowski M**, Ortmann D, Snijders K, Yiangou L, Cardoso de Brito M, et al. Optimized inducible shRNA and CRISPR/Cas9 platforms for in vitro studies of human development using hPSCs. Development 2016;143:4405–4418.

[11] Vallier L, Touboul T, Chng Z, Brimpari M, Hannan N, Millan E, et al. Early cell fate decisions of human embryonic stem cells and mouse epiblast stem cells are controlled by the same signalling pathways. PLoS One 2009;4:e6082.

[12] Hannan NR, Segeritz CP, Touboul T, Vallier L. Production of hepatocyte-like cells from human pluripotent stem cells. Nat Protoc 2013;8:430–437.

[13] Ran FA, Hsu PD, Wright J, Agarwala V, Scott DA, Zhang F. Genome engineering using the CRISPR-Cas9 system. Nat Protoc 2013;8:2281–2308.

[14] **John C**, **Reeve NF**, Free RC, Williams AT, Ntalla I, Farmaki A-E, et al. Cohort profile: extended cohort for e-health, environment and DNA (EXCEED). Int J Epidemiol 2019;48:678–679j.

[15] Sakamoto O, Abukawa D, Takeyama J, Arai N, Nagano M, Hattori H, et al. An atypical case of abetalipoproteinaemia with severe fatty liver in the absence of steatorrhoea or acanthocytosis. Eur J Pediatr 2006;165:68–70.

[16] Nucleotide. Bethesda (MD): National Library of Medicine (US) NCfBIANN, Homo sapiens chromosome vol. 4. https://www.ncbi.nlm.nih.gov/nuccore/NC_000004.12. Accessed 26 June 2020.

[17] GEM Japan whole genome aggregation (GEM-J WGA) panel. https://grch38.togovar.org/doc/datasets/gem_j_wga. Accessed 17 March 2023.

[18] Chalmers J, Ban L, Leena KB, Edwards KL, Grove JL, Aithal GP, et al. Cohort profile: the Trivandrum non-alcoholic fatty liver disease (NAFLD) cohort. BMJ Open 2019;9:e027244.
[19] rs745447480 SNP. http://www.ensembl.org/Homo_sapiens/Variation/Explore?r=4:99608399-99609399;v=rs745447480;vdb=variation;vf=126097885. Accessed 26 June 2020.
[20] Prill S, Caddeo A, Baselli G, Jamialahmadi O, Dongiovanni P, Rametta R, et al. The TM6SF2 E167K genetic variant induces lipid biosynthesis and reduces apolipoprotein B secretion in human hepatic 3D spheroids. Sci Rep 2019;9:11585.
[21] Kim DS, Jackson AU, Li YK, Stringham HM, Kuusisto J, Kangas AJ, et al. Novel association of *TM6SF2* rs58542926 genotype with increased serum tyrosine levels and decreased apoB-100 particles in Finns. J Lipid Res 2017;58:1471–1481.
[22] **Pirazzi C**, **Adiels M**, Burza MA, Mancina RM, Levin M, Ståhlman M, et al. Patatin-like phospholipase domain-containing 3 (*PNPLA3*) I148M (rs738409) affects hepatic VLDL secretion in humans and *in vitro*. J Hepatol 2012;57:1276–1282.
[23] Zamel R, Khan R, Pollex RL, Hegele RA. Abetalipoproteinemia: two case reports and literature review. Orphanet J Rare Dis 2008;3:19.
[24] Wetterau JR, Aggerbeck LP, Bouma ME, Eisenberg C, Munck A, Hermier M, et al. Absence of microsomal triglyceride transfer protein in individuals with abetalipoproteinemia. Science 1992;258:999–1001.
[25] Gündüz M, Özaydın E, Atar MB, Koç N, Kırsaçlıoğlu C, Köse G, et al. Microsomal triglyceride transfer protein gene mutations in Turkish children: a novel mutation and clinical follow up. Indian J Gastroenterol 2016;35:236–241.
[26] Fairfield CJ, Drake TM, Pius R, Bretherick AD, Campbell A, Clark DW, et al. Genome-wide association study of NAFLD using electronic health records. Hepatol Commun 2022;6:297–308.
[27] Vujkovic M, Ramdas S, Lorenz KM, Guo X, Darlay R, Cordell HJ, et al. A multiancestry genome-wide association study of unexplained chronic ALT elevation as a proxy for nonalcoholic fatty liver disease with histological and radiological validation. Nat Genet 2022;54:761–771.
[28] Khatun I, Walsh MT, Hussain MM. Loss of both phospholipid and triglyceride transfer activities of microsomal triglyceride transfer protein in abetalipoproteinemia. J Lipid Res 2013;54:1541–1549.
[29] Li L, Wang SJ, Shi K, Chen D, Jia H, Zhu J. Correlation between MTP -493G>T polymorphism and non-alcoholic fatty liver disease risk: a meta-analysis. Genet Mol Res 2014;13:10150–10161.
[30] Musso G, Gambino R, Cassader M. Lipoprotein metabolism mediates the association of MTP polymorphism with beta-cell dysfunction in healthy subjects and in nondiabetic normolipidemic patients with nonalcoholic steatohepatitis. J Nutr Biochem 2010;21:834–840.
[31] Di Filippo M, Varret M, Boehm V, Rabès JP, Ferkdadji L, Abramowitz L, et al. Postprandial lipid absorption in seven heterozygous carriers of deleterious variants of MTTP in two abetalipoproteinemic families. J Clin Lipidol 2019;13:201–212.
[32] Di Filippo M, Créhalet H, Samson-Bouma ME, Bonnet V, Aggerbeck LP, Rabès JP, et al. Molecular and functional analysis of two new MTTP gene mutations in an atypical case of abetalipoproteinemia. J Lipid Res 2012;53:548–555.
[33] Welty FK. Hypobetalipoproteinemia and abetalipoproteinemia: liver disease and cardiovascular disease. Curr Opin Lipidol 2020;31:49–55.
[34] Pelusi S, Baselli G, Pietrelli A, Dongiovanni P, Donati B, McCain MV, et al. Rare pathogenic variants predispose to hepatocellular carcinoma in nonalcoholic fatty liver disease. Sci Rep 2019;9:3682.
[35] Taylor R. Pathogenesis of type 2 diabetes: tracing the reverse route from cure to cause. Diabetologia 2008;51:1781–1789.
[36] Luukkonen PK, Zhou Y, Nidhina Haridas PA, Dwivedi OP, Hyötyläinen T, Ali A, et al. Impaired hepatic lipid synthesis from polyunsaturated fatty acids in *TM6SF2* E167K variant carriers with NAFLD. J Hepatol 2017;67:128–136.
[37] Bianco C, Tavaglione F, Romeo S, Valenti L. Genetic risk scores and personalization of care in fatty liver disease. Curr Opin Pharmacol 2021;61:6–11.
[38] Nakajima K, Nakano T, Tokita Y, Nagamine T, Inazu A, Kobayashi J, et al. Postprandial lipoprotein metabolism: VLDL vs chylomicrons. Clin Chim Acta 2011;412:1306–1318.
[39] Ajmera V, Park CC, Caussy C, Singh S, Hernandez C, Bettencourt R, et al. Magnetic resonance imaging proton density fat fraction associates with progression of fibrosis in patients with nonalcoholic fatty liver disease. Gastroenterology 2018;155:307–310.e302.
[40] Schwabe RF, Tabas I, Pajvani UB. Mechanisms of fibrosis development in nonalcoholic steatohepatitis. Gastroenterology 2020;158:1913–1928.
[41] Friedman SL, Neuschwander-Tetri BA, Rinella M, Sanyal AJ. Mechanisms of NAFLD development and therapeutic strategies. Nat Med 2018;24:908–922.
[42] **Liu Y**, **Conlon DM**, **Bi X**, **Slovik KJ**, Shi J, Edelstein HI, et al. Lack of MTTP activity in pluripotent stem cell-derived hepatocytes and cardiomyocytes abolishes apoB secretion and increases cell stress. Cell Rep 2017;19:1456–1466.
[43] Reich D, Thangaraj K, Patterson N, Price AL, Singh L. Reconstructing Indian population history. Nature 2009;461:489–494.

Journal of Hepatology, Volume *5*

Supplemental information

Identification and characterisation of a rare *MTTP* variant underlying hereditary non-alcoholic fatty liver disease

Jane I. Grove, Peggy C.K. Lo, Nick Shrine, Julian Barwell, Louise V. Wain, Martin D. Tobin, Andrew M. Salter, Aditi N. Borkar, Sara Cuevas-Ocaña, Neil Bennett, Catherine John, Ioanna Ntalla, Gabriela E. Jones, Christopher P. Neal, Mervyn G. Thomas, Helen Kuht, Pankaj Gupta, Vishwaraj M. Vemala, Allister Grant, Adeolu B. Adewoye, Kotacherry T. Shenoy, Leena K. Balakumaran, Edward J. Hollox, Nicholas R.F. Hannan, and Guruprasad P. Aithal

Supplementary materials

Identification and characterisation of a rare *MTTP* variant underlying hereditary non-alcoholic fatty liver disease.

Jane I. Grove, Peggy Cho Kiu Lo, Nick Shrine , Julian Barwell, Louise V. Wain, Martin D. Tobin , Andrew M. Salter, Aditi Borkar, Sara Cuevas-Ocana, Neil Bennett, Catherine John, Ioanna Ntalla, Gabriela E. Jones, Christopher P. Neal, Mervyn G. Thomas, Helen Kuht , Pankaj Gupta, Vishwaraj M. Vemala, Allister Grant, Adeolu B. Adewoye, Kotacherry T. Shenoy , Leena K. Balakumaran, Edward J. Hollox, Nicholas R.F. Hannan, Guruprasad P. Aithal

Table of Contents:

Supplemental Methods……………………………………………………………………2

Supplemental Figures…………………………………………………………….......13

Supplemental Tables…………………………………………………………………….28

Supplemental References…………………………………………………………....34

Supplemental Methods

See CTAT table for addition details of materials

Patient Investigations

Family members were screened at a joint hepatology-genetics clinic. Liver ultrasound and fibroscan® were used to detect evidence of steatosis and if seen in the context of abnormal liver function tests, NAFLD was diagnosed. Details of clinical investigations: Eyes: any evidence of night blindness, progressive reduced vision, retinitis pigmentosa, nystagmus and ophthalmoplegia; Neurological: ataxia (if any what age of onset), slurred speech, muscle weakness, loss of reflexes; Skeletal: lordosis, kyphoscoliosis, high arched feet (pes cavus, club foot); Gastrointestinal: fat malabsorption, greasy pale stools, steatorrhea; Developmental: failure to thrive, intellectual disability, developmental delay; blood tests: peripheral smear for acanthocytosis, low number of red blood cells; lipid profile: hypocholesterolaemia; duodenal biopsy; liver profiles, ultrasound and fibroscan®, or biopsy.

Meal Response Study

Foods: Breakfast was 70g Kelloggs Cornflakes, 300ml Tesco British Whole Milk with 35g Nestle Lido full cream milk powder (total: 633kcals; 21.3g fat; 84.9g carbohydrate; 23.5g protein; 2.1g fibre; 1.5g salt). Lunch was Sainsbury's Indian Vegetable Biryani (500g) meal and milkshake containing 200ml Tesco whole milk, 20ml Tesco fresh double cream and Kelly's clotted cream vanilla ice cream (total: 1134kcals; 58.7g fat; 119.9g carbohydrate; 23.9g protein; 15.7g fibre; 1.77g salt).

Blood Sampling

Blood samples were collected in vacutainer tubes containing: 5mg sodium fluoride and 4mg potassium oxalate (for glucose analysis), lithium heparin, potassium EDTA and stored on ice, or in tubes without additive (all Becton Dickinson) for serum after coagulation at room temperature for 30 min. For free fatty acid analysis 45µl of glutathione/ethylene glycol tetra acetic acid was added

per 6ml blood in with lithium heparin (Sigma-Aldrich) and 15µl Tetrahydrolipostatin was also added to inhibit lipase activity[1]. Plasma free fatty acids were measured using Wako NEFA C enzymatic colour test method (Wako Chemicals GmbH). Caspase-cleaved CK-18 was quantified in duplicate using M30 Apoptosense ELISA (Peviva, Sweden). Blood was centrifuged at 2000g for 10 min either at room temperature for serum, or at 4°C for plasma and the upper layer transferred to cryovials. EDTA blood was collected for DNA extraction. Whole blood, plasma and serum samples were stored at -80°C prior to metabolic analyses, or used immediately.

Lipoprotein Preparation

Lipoprotein fractions were prepared from plasma within 12h of blood sampling.
Plasma lipoproteins were separated by sequential non-equilibrium density-gradient ultracentrifugation by established techniques based on those originally described [2]. Up to 3 ml EDTA plasma was pipetted into quick-seal ultracentrifuge tubes (Beckman Coulter, Inc., High Wycombe, UK) and were topped up with 1.006 g/ml potassium bromide (KBr) solution and sealed. The tubes were centrifuged in a Beckman Optima ultracentrifuge XL-70 under vacuum at 12°C using rotor 50.4 for 20 min at 12,000 rpm with full acceleration and no break. Afterwards each tube was cut open using a tube slicer (Kontron, Watford, UK). The top chylomicron layer was taken and made up to 2 ml with 1.006 g/ml KBr solution and stored at -20°C The lower lipoprotein layer was then transferred to fresh ultracentrifuge tubes and centrifuged under vacuum at 12°C using rotor 50.4 for 16 hrs at 39,000 rpm with full acceleration and no break. Following ultracentrifugation the top VLDL layer was removed, this and the lower layer were separately stored at -20°C.
Quantitative determination of TAG and cholesterol was carried out on the plasma, chylomicron and VLDL fractions using Infinity™ TAG and Cholesterol Liquid Stable Reagent kits (Thermo Fisher Scientific, UK), according to the manufacturer's instructions.

Variant identification

DNA was derived from blood except the 3 EXCEED study samples[3] which were derived from saliva. Whole exome sequencing (single batch with 3 replicates) mean depth of coverage was 42-66×. Exome enrichment was done in 3 batches using NimbleGen SeqCap EZ Exome v3.0 (64Mb). Samples were sequenced using 100bp paired-end sequencing on the Illumina HiSeq2000 (each sample sequenced in 2 lanes) and validated by Sanger sequencing. Both NAFLD cases and controls were present in each pair of lanes and in each batch to minimise confounding.

After alignment[4], reads were cleaned with Picard v1.93 (synchronise mate-pair information) and SAMtools[5] (PCR duplicates removed, sorted and indexed); local realignment around indels and recalibration of quality scores was done with GATK v3.2-2[6]. Variant calling was done using both GATK v3.2-2 HaplotypeCaller and SAMtools v1.1 mpileup to obtain a consensus. Concordance between the two methods was 96.8%. Concordance between the 3 pairs of replicate samples was 98.4-98.6%.

First we identified variants that were not called in any of the 9 control samples. This identified 8,835 SNPs unique to one or more NAFLD cases. We next excluded any SNPs that were also present in the following datasets: 1000 genomes phase I high confidence SNPs[7], 1000 Genomes Project (May 2013 release), 1000 genomes Illumina OMNI 2.5 SNP array[8], dbSNP version 138 and 142[9] (https://www.ncbi.nlm.nih.gov/snp/) HapMap3[10] (https://www.sanger.ac.uk/resources/downloads/human/hapmap3.html), NHLBI exomes[11] (March 2015; https://evs.gs.washington.edu/EVS/) and whole exome sequencing of 125 South Asian samples[12]. We restricted the remaining 1024 SNPs to those annotated as 'exonic' using ANNOVAR[13] (November 2014 version) which left 434. Of those, Variant Effect Predictor[14] annotated 159 SNPs as 'deleterious' by SIFT[15], 'probably damaging' or 'potentially damaging' by PolyPhen-2[16], or had a CADD[17] Phred scaled score≥20 or GWAVA[18] score >0.5. Segregation with disease was assessed by overlaying the variant genotypes on the pedigree (Fig. 1A).

A missense variant in *MTTP* seen in all 12 affected individuals (6 heterozygotes and 6 homozygotes) was the only variant that fully segregated with disease. Two additional variants were found as heterozygotes: N1484S in *NOTCH1* NC_000009.11:g.139399897T>C (10 cases) and G796R in *EPB41L1* NC_000020.10:g.34807716G>C (11 cases). The sequence was analysed via Phyre2.0[19] and SuSPect[20] webservers to study the effects of mutations on the protein structure, stability and possible function.

Genotyping

Genomic DNA was prepared using Flexigene DNA kit (Qiagen). PCR-RFLP genotyping used primers *MTTP*-F1 and *MTTP*-R1 followed by restriction digestion with Hpy166II and analysis by agarose gel electrophoresis. PCR-RFLP was used for genotyping rs738409 (FokI), rs58542926 (MspI) and rs58542926 (Hpy188I)[21]. For analysis of *MTTP* alleles, following removal of PCR components, the sequences of PCR products generated were determined using Sanger sequencing (Source Bioscience, Nottingham, UK). The following primer pairs were used to amplify DNA for genotyping common genetic variants: rs745447480: TCTTAACGGCCTCAGCCTAG & CAGAGTTACCAGTCATGGACTC; rs2306986 and rs3816873: AAGGTAGAATAGGGCAGGGGTCC & CTAATCTCAGTTGGATCATTTCAGTCTC; rs3792683: GTTACAGGTAGAGAACATGCTGACATG & CCTCCATGGTACAGTGGTGCAC; rs2306985: CAGTCACAGAGTCCTACCCAGG & GAGACTGCTGTCATCACAACTCTGTG; rs738409: CAGCTGTGGCTACTCTGTCTG & TGGAGAAAGCTTATGAAGGATCAG; rs58542926: CCAAAATGTTGGGATTACAGG & ACAGATGTCCAGCAGGGTTC.

Isolation and culture of human dermal fibroblasts

Primary human dermal fibroblasts were established via explant culture in DMEM medium supplemented with 2% Antibiotic-Antimycotic; 10% FBS; 1% GlutaMAX; 1% NEAA and 1% penicillin/streptomycin. Fibroblast medium was refreshed every 2-3 days, cells were split at 1:3-1:6

using 0.25% Trypsin-EDTA for 3min at 37°C when 80% confluent. All reagents were from Gibco (ThermoFisher, UK).

Fibroblast reprogramming and hiPSC maintenance

Skin biopsies from participant 1, genotyped as MTP564-II, and family member J, MTP564-TT, were dissected to remove subcutaneous fat and cultured in fibroblast media for approximately 10 days until fibroblasts began emerging from the biopsy forming a monolayer. Approximately 20,000 fibroblasts were seeded in a well of 6-well plate, cells were transduced 24h later at a MOI of 5:5:3 (hKOS: hc-Myc: hKlf4). Transduced cells were maintained in fibroblast medium for 3-7 days until they were 80-90% confluent, then transferred to a new 6-well plate at a split ratio of 1:3 using 0.05% Trypsin (Gibco) and switched to TeSR-E7 medium (StemCell Technologies, Cambridge, UK). When hiPSC colonies appeared around 13-22 days post-transduction, medium was replaced to TeSR-E8 (StemCell Technologies). Colonies were selected between day 13-45 post-transduction using either 0.5mM EDTA (Invitrogen), or ReLeSR (StemCell Technologies). Once the hiPSC lines were established, culture medium was transitioned from TeSR-E8 to an E8 medium (prepared on site) following the formula described previously (50), with an additional 100 ng/ml heparin sodium salt. HiPSCs were passaged every 3-4 days (75-90%) using TrypLE Express (Gibco) at 1:10-1:20 ratio and seeded onto matrigel- coated (Corning) Nunc plasticware (Thermofisher). E8 medium was supplemented with 10 μM Y27632 ROCK inhibitor (ROCKi; Tocris) for the initial 24h after splitting. The first undifferentiated colonies appeared 7 days post transduction, stable colonies were picked after 3 weeks and stable cell lines generated approximately 40 days post transduction. All cell lines tested negative for mycoplasma contamination using the EZ-PCR Mycoplasma Test Kit (Biological Industries) prior to reprogramming.

Differentiation to embryonic germ layers

For mesoderm differentiation hiPSCs were differentiated as described previously[22]. Briefly, hiPSCs were seeded at 20,000 cells/cm^2 on Matrigel-coated 48-well plate. 72 h later E8 medium

was replaced to mesoderm induction medium consisting of RPMI (Gibco), 213 µg/ml ascorbic acid and 500 µg/ml albumin (both from Sigma-Aldrich). 4 µM CHIR99021 was supplemented to the medium for the first two days. Cells were fixed with 4% paraformadehyde on day 4 for immunostaining.

For ectoderm differentiation hiPSCs were differentiated as described previously [23]. Briefly, hiPSCs were seeded onto Matrigel-coated 48-well plate at a density of 50,000 cells/cm^2. 24 h after seeding, E8 medium was replaced to RPMI medium supplemented with 2% B27; 1% NEAA; 10 µM SB431542 (Selleckchem) and 12 ng/ml FGF2 (Peprotech), medium was replaced daily.

Differentiation of hiPSCs into hepatocyte-like cells (HLCs)

All cells were differentiated into hepatocytes as described previously [24]. Briefly, hiPSCs were seeded at a density of 15,000-20,000 cells/cm^2 (dependant on the cell line) onto Matrigel-coated plasticware. Definitive endoderm differentiation was initiated 48h after seeding when cells were approximately 50% confluent, hiPSCs were cultured in RPMI with 2% B27 and 1% NEAA as basal medium, then supplemented with 100 ng/ml activin A and 50 ng/ml Wnt-3a (R&D) for 3 days. Foregut was generated by incubating cells in RPMI/B27/NEAA with 50 ng/ml activin A for an additional 2 days. Hepatic endoderm specification was achieved by supplementing RPMI/B27/NEAA with 10 µM SB431542, 10 ng/ml BMP4 (R&D Systems) and 20 ng/ml FGF10 for 4 days. Finally, medium was replaced every other day to HepatoZYME (Gibco) with 1% chemically defined-lipid concentrate (Gibco); 1% GlutaMAX; 1% NEAA; HGF; and oncostatin-M.

CRISPR-Cas9 mediated correction of I564T mutation in the MTTP$^{(VAR/VAR)}$

Nucleofection of the *MTTP*$^{(VAR/VAR)}$ parental line was optimised according to the manufacturer's recommendations (Lonza) using the provided eGFP plasmid and transfection efficiency was quantified by flow cytometry (Supplementary Fig. 10A). For CRISPR-Cas9 editing (Supplementary Fig. 10B-F), a single-guide RNA (5'-gaacatcctgctgtctactg) was cloned into the #51133 plasmid (Addgene) [25] and nucleofected into the *MTTP*$^{(VAR/VAR)}$ parental line along with #62988 (Addgene)

[26]. Plasmid # 51133 (pGL3-U6-sgRNA-PGK-puromycin) was a gift from Xingxu Huang [25] and #62988 (pSpCas9(BB)-2A-Puro (PX459) V2.0) was a gift from Feng Zhang [26]. Clones were manually isolated and screened for correction of the I564T mutation $MTTP^{(VAR/VAR)}$ to $MTTP^{(WT*/WT*)}$ (C>T) using PCR amplification of the target site (primers FWD and REV) followed by restriction digestion with Hpy166II (New England Biolabs) that specifically detect $MTTP^{(VAR/VAR)}$. Genetic modifications in the selected clones were confirmed by Sanger sequencing analysis (Source Bioscience Ltd, UK). Corrected $MTTP^{(WT*/WT*)}$ hiPSCs and the $MTTP^{(VAR/VAR)}$ parental hiPSC line were differentiated to HLCs in parallel for characterisation.

Karyotyping

30 metaphase spreads from exponentially growing hiPSC cultures were analysed by conventional karyotyping[27] (Nottingham University Hospitals NHS Trust).

Detection of cellular and mitochondrial ROS

Mitochondria content of HLCs were visualised using 100 nM MitoTracker green FM or MitoTracker deep red FM. Intracellular reactive oxygen species (ROS) and mitochondrial superoxide production were assessed using 2.5 µM CellROX green or 2.5 µM MitoSox Red respectively. Nuclei counterstaining was achieved using 5 µg/ml of Hoechst 33342 (Invitrogen). All reagents were purchased from Invitrogen and used according to manufacturer's guidance. Live cells were loaded with the dyes for 30 minutes at 37°C and 5% CO_2. Cells were then replaced with warm HepatoZYME (Gibco) media and imaged using the Operetta system (PerkinElmer). Images were captured at 445nm (Hoechst 33342), 525nm (CellROX green and MitoTracker green) and 705nm (MitoSOX Red and MitoTracker deep red). Fluorescence intensity was analysed using Columbus system (PerkinElmer) and plotted as mean ± SD from multiple image acquisition fields.

Mitochondrial respiration analysis

HLCs were dissociated using TrypLE Express (Gibco) and seeded onto XF96 plate with a density of 25,000-50,000 cells per well. HLCs were maintained for further 6 days and mitochondrial respiration analysed at D20. To assess mitochondrial respiration, culture medium was replaced with 200 µl Seahorse XF base medium supplemented with 10 mM glucose, 1 mM sodium pyruvate and 2 mM L-glutamine at 37°C without CO_2 for 1 h prior to measurements using the Seahorse XF96 analyser (Seahorse Bioscience, USA). Mitochondria stress tests were performed as recommended by the manufacturer, oxygen consumption rate (OCR) was measured while injecting oligomycin (1.5 µM), FCCP (0.4 µM), rotenone (1 µM) and XF base medium. OCR values were normalised by the number of viable cells counted with DAPI. All reagents were from Agilent.

RT-qPCR Gene expression analysis

Total RNA was isolated from HLCs cells using RNeasy Mini kit (Qiagen). Total RNA (500 µg) was reverse transcribed using the SuperScript II Reverse Transcriptase kit (Invitrogen) with random primers (Promega) and dNTP (Promega) according to the manufacturer's recommendations. Quantitative real-time PCR (qPCR) was performed as described previously [28, 29]: after cDNA samples diluted to a final volume of 600 µl with nuclease free water. qPCR reactions were run using cDNA samples (from initial 500 ng of reverse-transcribed RNA, diluted 1:30); 5 µM forward and reverse primers; with SensiMix SYBR & Fluorescein Kit (Bioline). qPCR amplification was performed on an Applied Biosystems 7500 Fast Real-Time PCR Systems using a three-step cycling programme of 40 cycles consisting incubation at 95°C (15 sec), 60°C (30 sec) and 72°C (30 sec). All samples were run with three technical replicates. Fold changes in expression of differentiating cells over undifferentiated hiPSCs were calculated using comparative ΔΔCt method standardised against the housekeeping gene PBGD, data were shown as mean of Ct values ± standard error of mean (SEM).

RNA sequencing and analysis

Total RNA was isolated from hiPSC-HLCs on day 21 using RNeasy Mini Kit (Qiagen). RNA yield was quantified by a Qubit Fluorometer (Invitrogen) with the Qubit RNA BR Assay Kit (ThermoFisher). RNA quality was assessed by Agilent RNA ScreenTape Assay kit all samples had an integrity number (RIN) ≥ 9.8. NEBNext rRNA Depletion Kit (New England Biolabs) was used to enrich mRNAs, depleted RNA samples were assessed using Agilent High Sensitivity RNA ScreenTape Assay Kit (RIN ranges 1.0-5.4). NEBNext Ultra II Directional RNA Library Prep Kit for Illumina (Set 1 and 2 indexes) was used to construct cDNA libraries. Concentration and sizes of the generated cDNAs were measured using Agilent High Sensitivity D1000 ScreenTape Assay Kit. 4200 TapeStation System (Agilent) was used to evaluate the quality of RNA and cDNA library. EnrichR was used to query the likely tissue and cell types based on gene expression [30]. Sequencing was done using the Illumina HiSeq 2500 system (high output mode) to yield targeted number of single-end 100bp reads to a depth of 30 million per sample. Reads were mapped to the GRCh38.p10 Ensembl human genome using Hisat2 (v2.1.0). Analysis was performed using SeqMonk (1.46.0) software, the read counts per gene was determined using RNA-Seq pipeline quantitation. Differential expression analysis was performed in R using DESeq2 (1.28.1) package. Data was trimmed with Trim Galore v0.6.2 using default parameters. It was aligned to the GRCh38 human genome assembly using Hisat2 v2.1.0 using the option "--sp 1000,1000" to prevent soft-clipping. The alignment was seeded with introns from gene models from Ensembl v87. Alignments with a MAPQ score of < 20 were discarded.

Per gene expression was quantitated against gene models from Ensembl v97 counting read overlaps to any exon of each gene. Only alignments on the opposite strand to the gene being measured were counted. For normalised expression visualisation log2 Reads per million reads of library (log2RPM) values were calculated, and these were then corrected using size factor normalisation based on genes which were measured in at least one replicate.

A similar process was employed for all differential expression calculations. An initial set of differentially expressed genes was calculated from raw counts using the DESeq2 package. Genes

with a FDR of <0.05 were retained. This list was further filtered using an expression normalised fold change z-score, again with a cut-off of FDR < 0.05. Final candidates were the intersection of the hits from these two tests.

Proteome profiler antibody arrays

Human NFκB Pathway, Phospho-Kinase and XL Cytokine Array Kits were all purchased from R&D Systems and used as specified by the manufacturer. Cell protein lysates were analysed using the NFκB and Phospho-kinase array kits; cell culture supernate from cultured HLCs collected 48 h after media change, was used for the XL Cytokine arrays.

Total protein concentration was quantified by NanoDrop-1000 spectrophotometer (Thermo-Fisher). Protein samples were incubated overnight and visualised the next day using ImageQuant LAS-4000 (Fujitsu Life Sciences). Image Studio Lite Software (version 5.2) was used to quantify the pixel intensity of each set of duplicated antibody spots and normalised by subtracting the averaged background signal. Comparison of protein expression was achieved by calculating the relative fold change with respect to signals produced by $MTTP^{(WT/WT)}$.

Immunocytochemistry

Cells were fixed in 4% paraformaldehyde (PFA; VWR Chemicals) for 20 min at 4°C, followed by 30 min blocking and membrane permeabilisation using 10% foetal bovine serum (FBS; Gibco) and 0.1% TritonX-100 (Thermo Scientific) in PBS (Gibco). Fixed cells were incubated overnight at 4°C in a combination of primary antibodies diluted in 1% FBS-PBST solution. The next day, cells were incubated with fluorescent-labelled secondary antibodies for 1 h at RT, followed by nuclei-counterstaining using 0.5 µg/ml DAPI (Sigma) in 1% FBS-PBST for 5 min. Antibodies used were MTTP (Abcam); OCT3/4 (Santa Cruz Biotechnology); NANOG (R&D Systems); MESP1 (Abcam); GATA-4 (R&D Systems); Nestin (Merck); SOX2 (Novus Biologicals); ALB (R&D Systems); CYP2A6 (OriGene Technologies); A1AT (Abcam); GST-pi (Enzo Life Sciences);

Secondary antibodies at 1/400 dilution: Donkey Anti-Mouse AF 488 (Invitrogen); Donkey Anti-Goat AF 647 (Invitrogen); Donkey Anti-Rabbit AF 647 (Invitrogen).

Microscopy

Cells were fixed and permeabilised and imaged on tissue culture plates containing PBS. All images were acquired using the Automated Operetta™ high content image analyser (PerkinElmer); captured images were then analysed using Columbus™ software (PerkinElmer), fluorescence intensity of specific channel was determined with the developed algorithms in the software. Fluorochromes were Alex Fluor 488 and 647. Images were captured at either 10x or 20x magnification as indicated. Expression was quantified in 10 fields in each. Mean fluorescence intensity in triplicate wells were compared using T-test.

Supplemental Figures

A

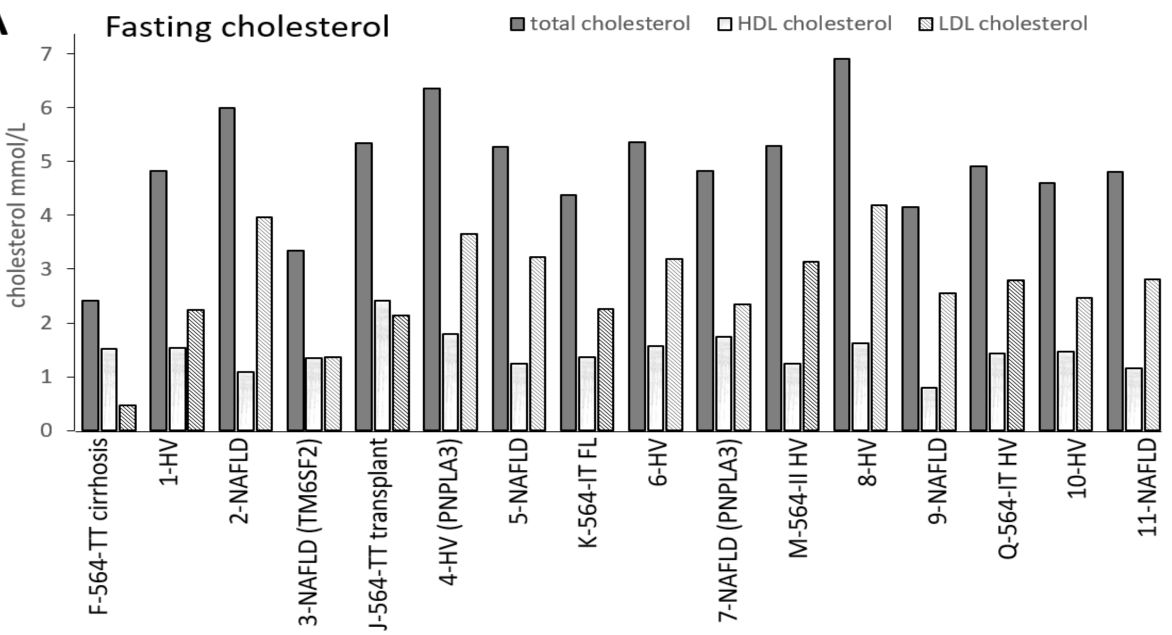

B

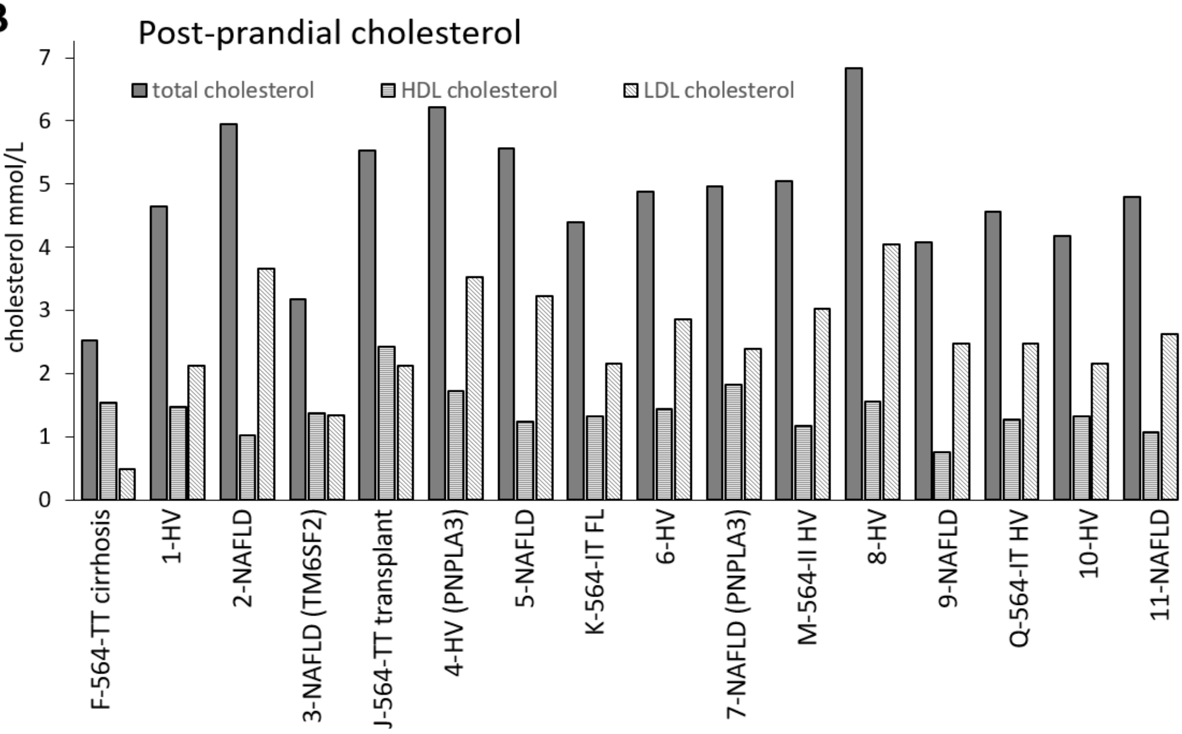

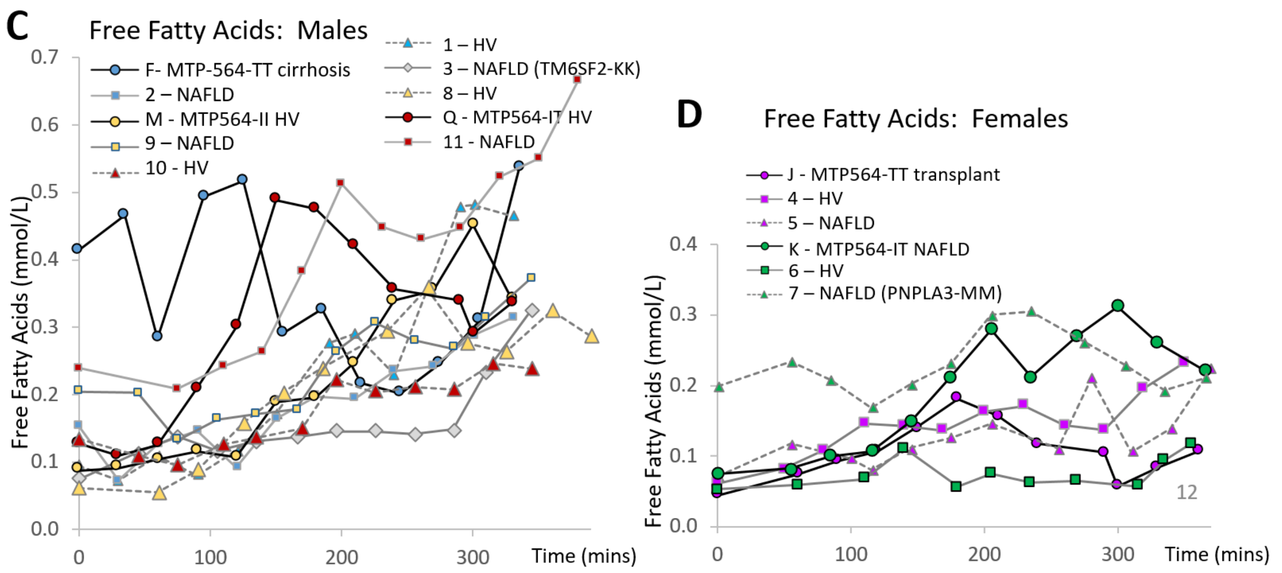

Figure S1. Serum cholesterol levels and free fatty acid levels in study participants.

Participants grouped according to age and gender matching with family member (Fig. 1a and Supplemental Table 3). Genes are shown in parentheses where participant is homozygous for other effect alleles: *PNPLA3* rs738409; *TM6SF2* rs58542926). (**A)** Fasting cholesterol. (**B)** Cholesterol level approx. 2h after eating standard study meal. (**C)** Free fatty acid levels in male participants. (**D)** Free fatty acid levels in female participants. NAFLD= non-alcoholic fatty liver disease; HV=healthy volunteer; FL=fatty liver. *PNPLA3* (rs738409) variant homozygotes (148-MM) and *TM6SF2* (rs58542926) variant homozygotes (167-KK) are indicated.

VLDL-triglycerides

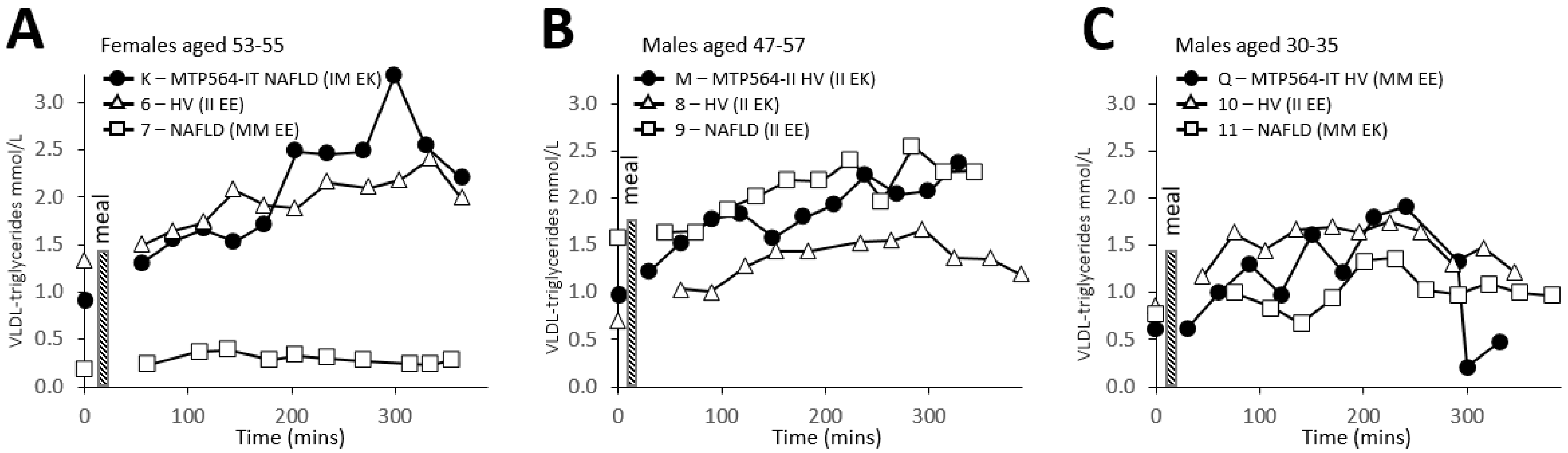

Chylomicron-triglycerides

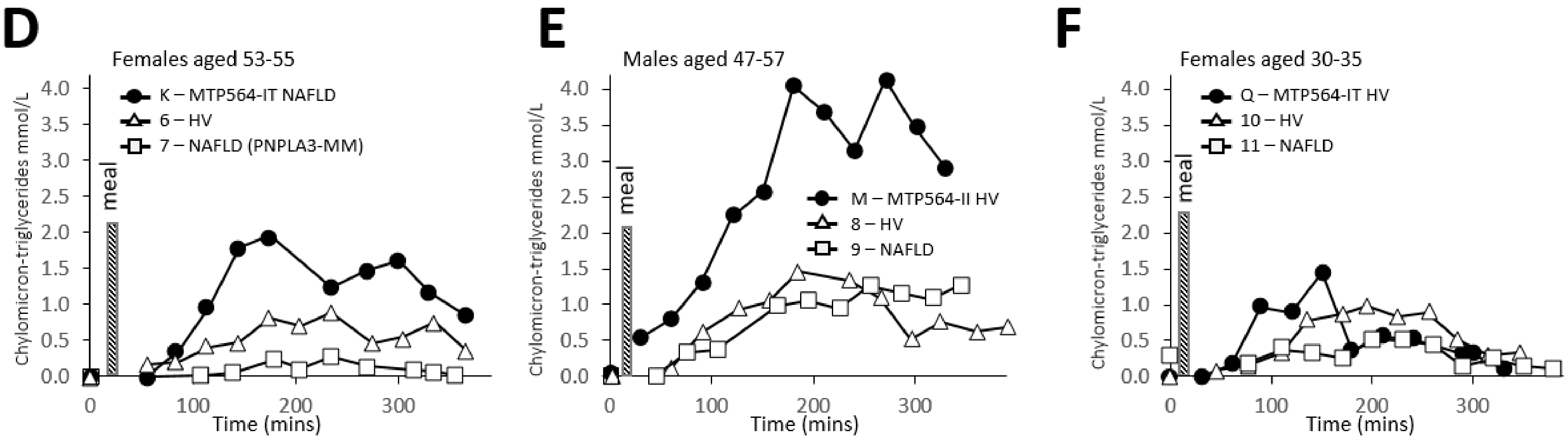

Figure S2. Lipoprotein-associated triglyceride levels in study participants before and after a fatty meal.

Participants are described in Supplemental Table 1**. (A)** Serum VLDL-triglycerides in MTP564-IT family member K and matched control participants: 6 (HV) and 7 (NAFLD patient). (**B)** VLDL-triglyceride in MTP564-II (wild type) family member M and matched control participants: 8 (HV) and 9 (NAFLD patient). (**C)** VLDL-triglycerides in MTP564-IT family member Q and matched control participants: 10 (HV) and 11 (NAFLD patient). (**D)** Chylomicron-triglycerides in participants K, 6 and 7. € Chylomicron-triglycerides in participants M, 8 and 9. (**F)** Chylomicron-triglycerides in participants Q, 10 and 11. *PNPLA3* p.I148M and *TM6SF2* pE167K genotypes are indicated in parentheses. HV=healthy volunteer; NAFLD=non-alcoholic fatty liver disease.

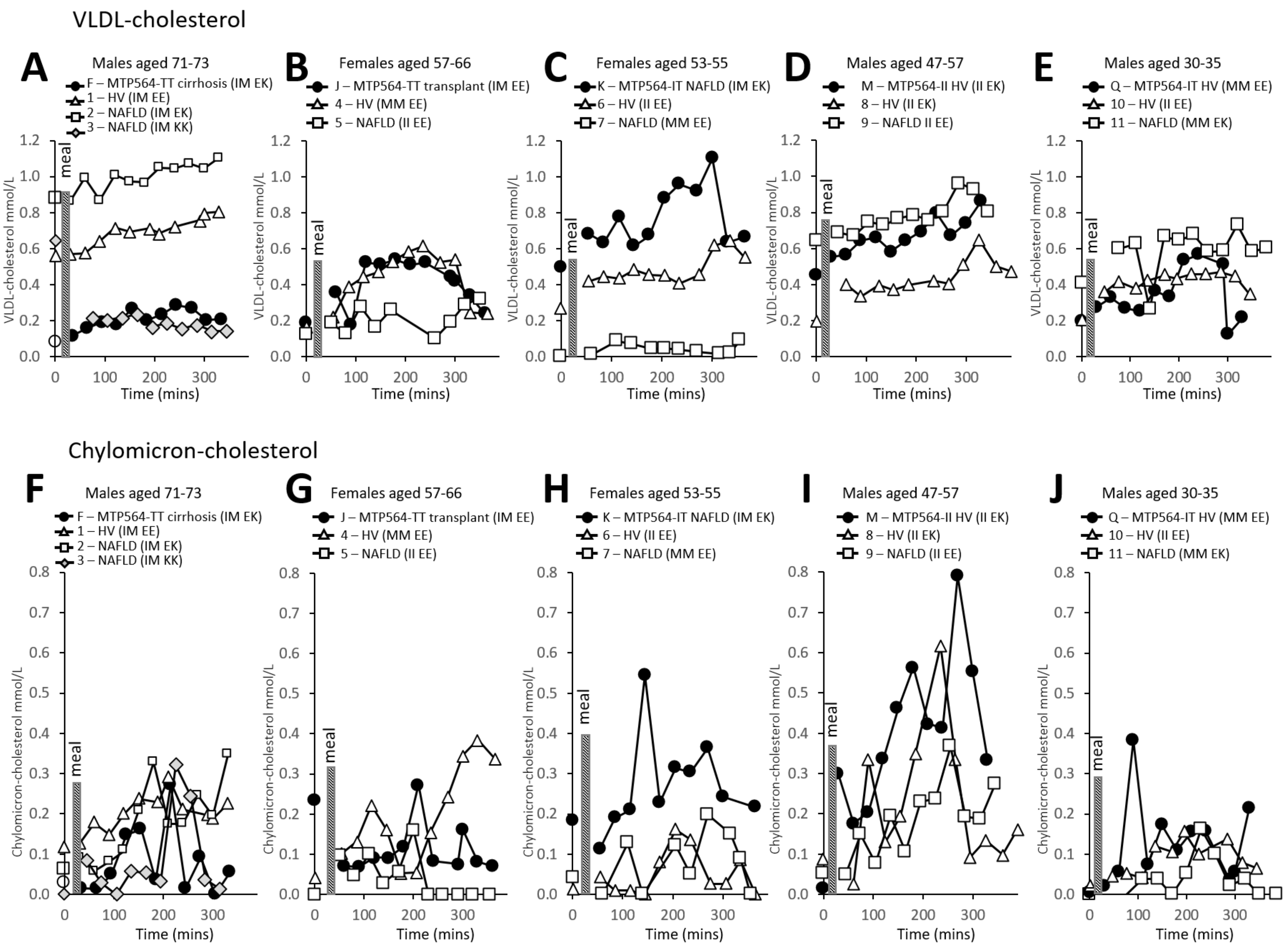

VLDL-cholesterol
A
Males aged 71-73
F – MTP564-TT cirrhosis (IM EK)
1 – HV (IM EE)
2 – NAFLD (IM EK)
3 – NAFLD (IM KK)
B
Females aged 57-66
J – MTP564-TT transplant (IM EE)
4 – HV (MM EE)
5 – NAFLD (II EE)
C
Females aged 53-55
K – MTP564-IT NAFLD (IM EK)
6 – HV (II EE)
7 – NAFLD (MM EE)
D
Males aged 47-57
M – MTP564-II HV (II EK)
8 – HV (II EK)
9 – NAFLD II EE)
E
Males aged 30-35
Q – MTP564-IT HV (MM EE)
10 – HV (II EE)
11 – NAFLD (MM EK)
VLDL-cholesterol mmol/L
meal
Time (mins)
Chylomicron-cholesterol
F
Males aged 71-73
F – MTP564-TT cirrhosis (IM EK)
1 – HV (IM EE)
2 – NAFLD (IM EK)
3 – NAFLD (IM KK)
G
Females aged 57-66
J – MTP564-TT transplant (IM EE)
4 – HV (MM EE)
5 – NAFLD (II EE)
H
Females aged 53-55
K – MTP564-IT NAFLD (IM EK)
6 – HV (II EE)
7 – NAFLD (MM EE)
I
Males aged 47-57
M – MTP564-II HV (II EK)
8 – HV (II EK)
9 – NAFLD (II EE)
J
Males aged 30-35
Q – MTP564-IT HV (MM EE)
10 – HV (II EE)
11 – NAFLD (MM EK)
Chylomicron-cholesterol mmol/L
meal
Time (mins)

Fig. S3. Lipoprotein-associated cholesterol levels in study participants before and after a high fat meal.

Participants are described in Supplemental Table 1. (**A)** VLDL-associated cholesterol levels in MTP564-TT family member F with cirrhosis and matched control participants: 1 (HV), 2 and 3 (NAFLD patients). **(B)** VLDL-cholesterol in MTP564-TT family member J with liver transplantand matched control participants: 4 (HV) and 5 (NAFLD patient). **(C)** VLDL-associated cholesterol levels in MTP564-IT family member K with NAFLD and matched control participants: 6 (HV) and 7 (NAFLD patient). (**D)** VLDL-associated cholesterol levels in MTP564-II (wild type) family member M and matched control participants 8 (HV) and 9 (NAFLD patient). **(E)** VLDL-associated cholesterol levels in MTP564-IT family member Q and matched control participants 10 (HV) and 11 (NAFLD patient). (**F)** Chylomicron**-**associated cholesterol in participants F, 1, 2 and 3. (**G)** Chylomicron**-**associated cholesterol in participants J, 4 and 5. (**H)** Chylomicron**-**associated cholesterol in participants K, 6 and 7. **(I)** Chylomicron**-**associated cholesterol in participants M, 8 and 9. (**J)** Chylomicron**-**associated cholesterol in participants Q, 10 and 11.

PNPLA3 p.I148M and *TM6SF2* pE167K genotypes are indicated in parentheses. HV=healthy volunteer; NAFLD=non-alcoholic fatty liver disease.

.

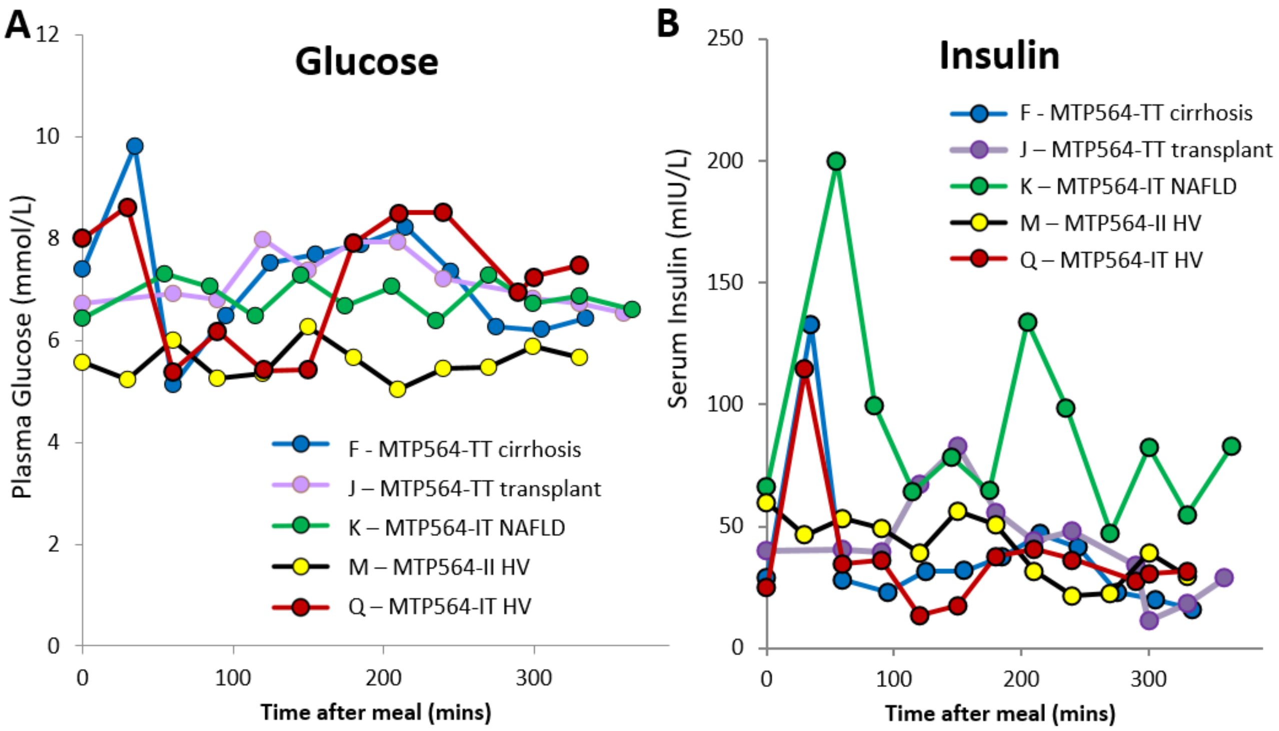

Fig. S4. Blood biomarker levels in family members after consuming a fatty meal.

(**A**) Plasma glucose. (**B**) Serum insulin.

PNPLA3 (rs738409) variant homozygotes (148-MM) and *TM6SF2* (rs58542926) variant homozygotes (167-KK) are indicated. HV=healthy volunteer; NAFLD=non-alcoholic fatty liver disease.

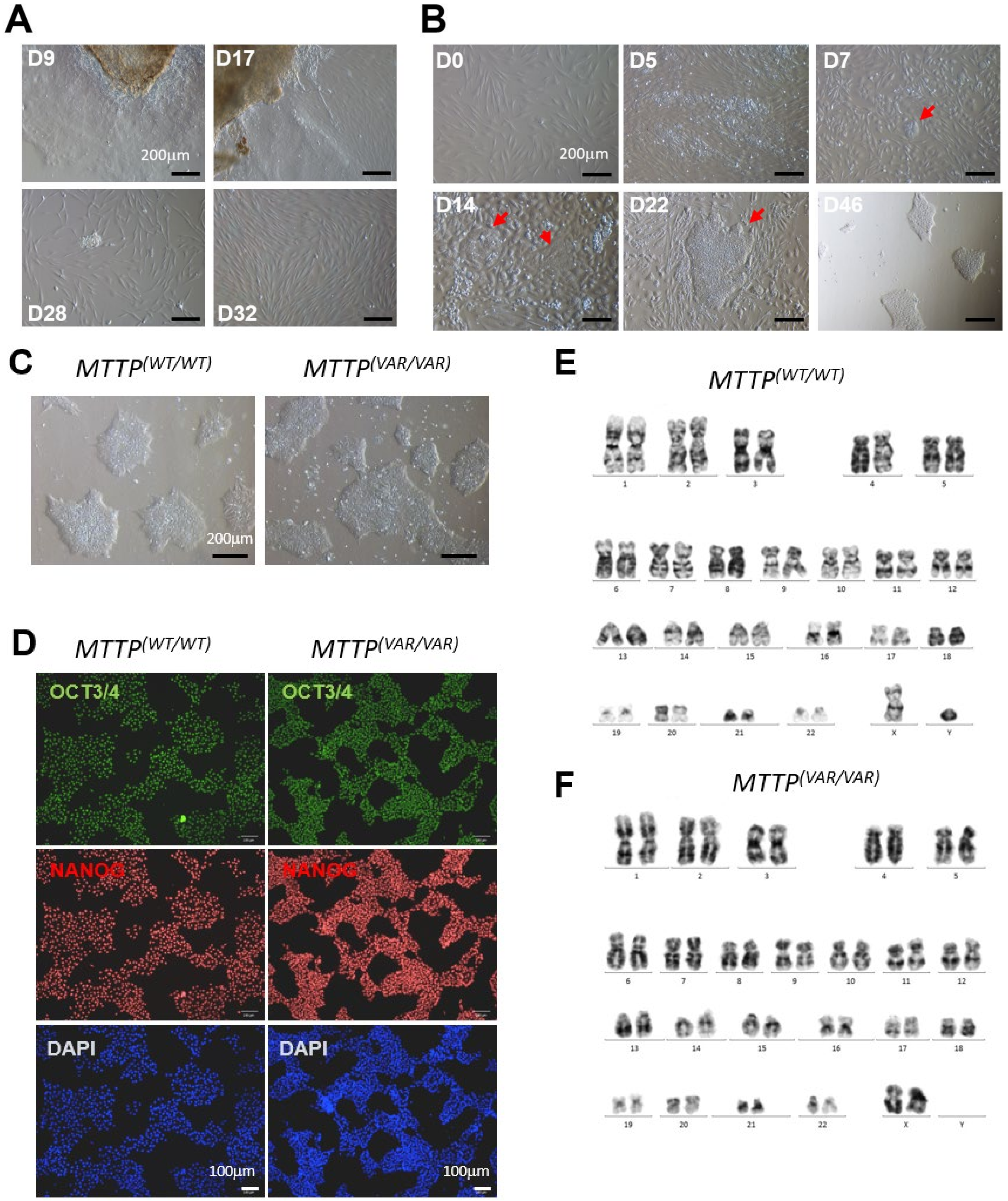

Fig. S5. Characteristics of hiPSCs derived from donor skin biopsy.
(**A**) Light microscopy showing representative skin biopsy fibroblast outgrowth at day 9, 17, 28 and 32. (**B**) Light microscopy showing representative images of fibroblast reprogramming at day 0, 5, 7, 14, 22 and 46 post viral transduction. Red arrows indicate emerging hIPSC colonies. (**C**) Light microscopy showing representative pictures of reprogrammed *MTTP*$^{(WT/WT)}$ and *MTTP*$^{(VAR/VAR)}$ hIPSC cultures. (**D**) Immunocytochemistry showing expression of pluripotency markers in *MTTP*$^{(WT/WT)}$ and *MTTP*$^{(VAR/VAR)}$ hiPSCs. (**E**), Karyotype of *MTTP*$^{(WT/WT)}$. (**F**) Karyotype of *MTTP*$^{(VAR/VAR)}$ hiPSCs.

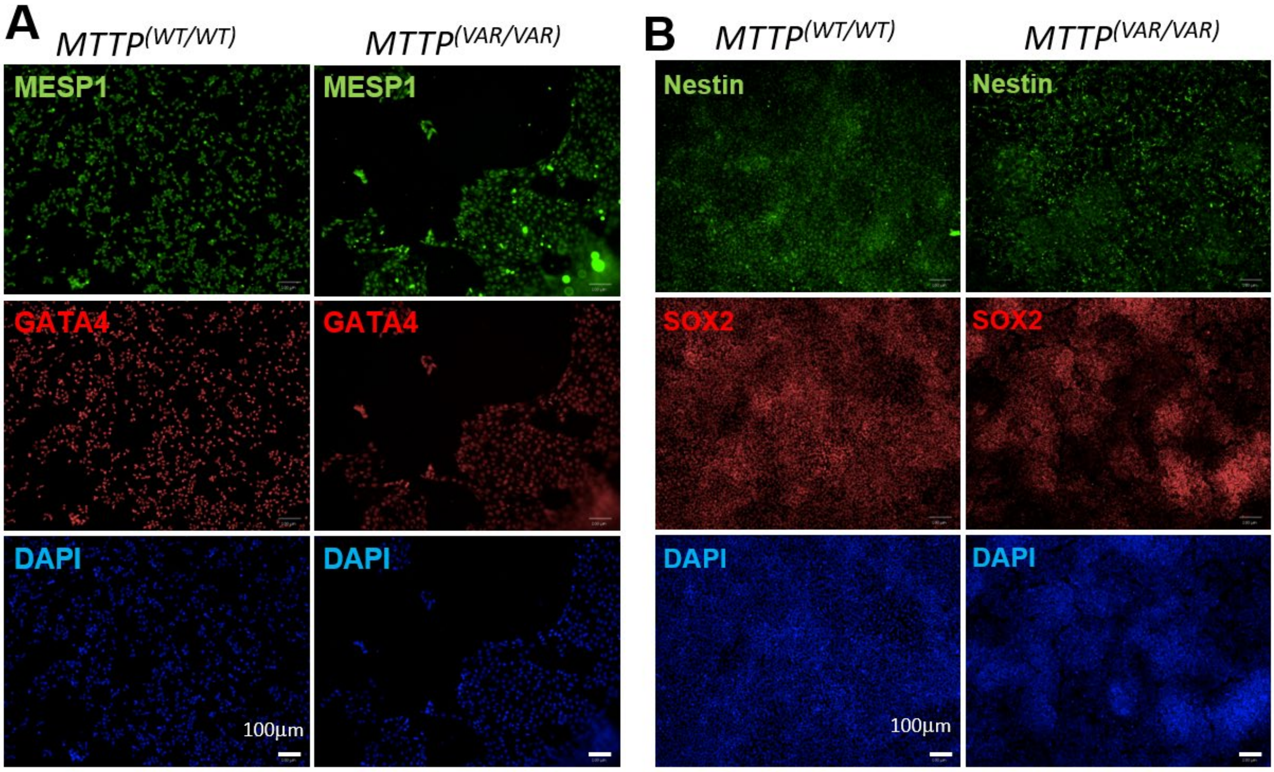

Fig. S6. Differentiation of *MTTP*$^{(WT/WT)}$ and *MTTP*$^{(VAR/VAR)}$ hiPSCs into multiple germ layers. (**A**) Expression of mesodermal genes following mesoderm differentiation of *MTTP*$^{(WT/WT)}$ and *MTTP*$^{(VAR/VAR)}$ hiPSCs by immunocytochemistry with MESP1, GATA4 and DAPI staining. (**B**) Expression of ectoderm genes following ectoderm differentiation of *MTTP*$^{(WT/WT)}$ and *MTTP*$^{(VAR/VAR)}$ hiPSCs by immunocytochemistry with Nestin, SOX2 and DAPI staining.

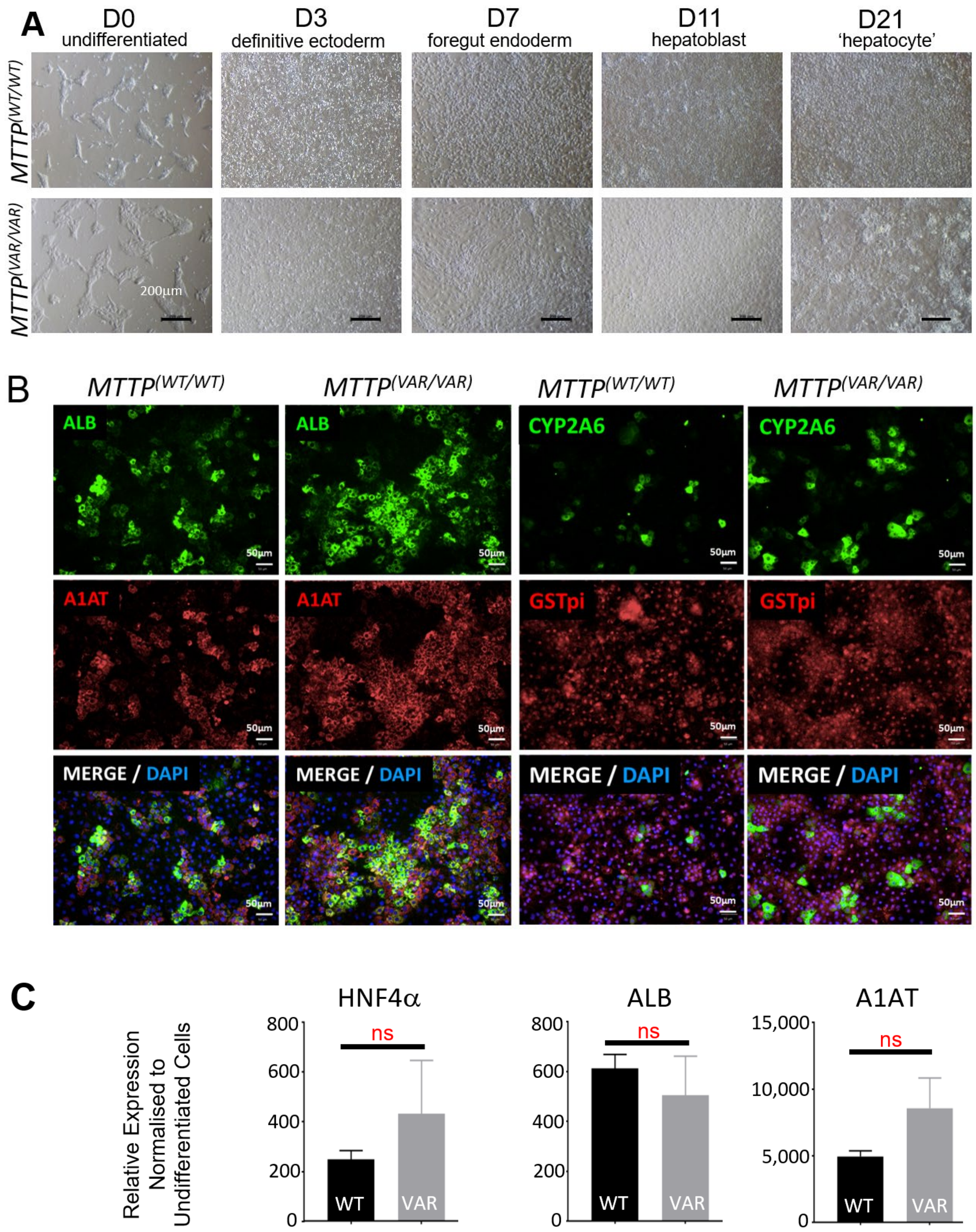

Fig. S7. Differentiation of *MTTP*$^{(WT/WT)}$ and *MTTP*$^{(VAR/VAR)}$ hiPSCs into mature HLCs. **(A)** Representative light microscopy images of *MTTP*$^{(WT/WT)}$ and *MTTP*$^{(VAR/VAR)}$ hiPSCs as they differentiate into HLCs including undifferentiated cells (Day 0), definitive endoderm (Day 3), foregut endoderm (Day 7), hepatoblast (Day 11) and 'hepatocytes' (Day 21). **(B)** Immunocytochemistry showing expression of hepatocyte markers in *MTTP*$^{(WT/WT)}$ and *MTTP*$^{(VAR/VAR)}$ hiPSC derived HLCs. (**C**) Light microscopy of Oil-Red-O stained *MTTP*$^{(WT/WT)}$ and *MTTP*$^{(VAR/VAR)}$ HLCs.

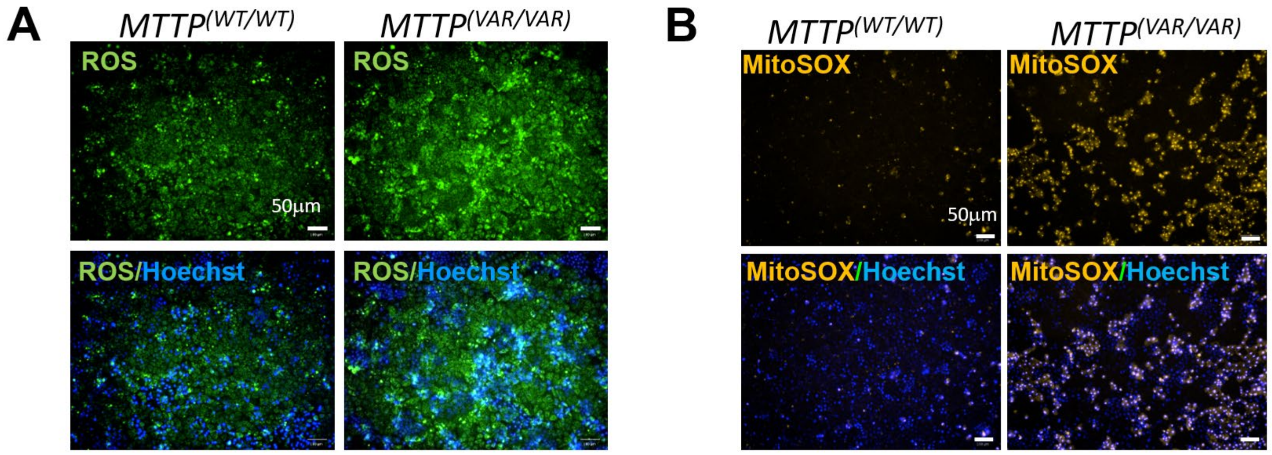

Fig. S8. Assessment of oxidative stress in MTP-564T homozygote variant, MTTP$^{(VAR/VAR)}$ and wild-type hepatocyte-like cells (HLCs).

(A) Reactive oxygen species detected in cells by fluorescence microscopy ±Hoechst stain.

(**B**) Superoxide presence in cells detected by fluorescence microscopy ±Hoechst stain.

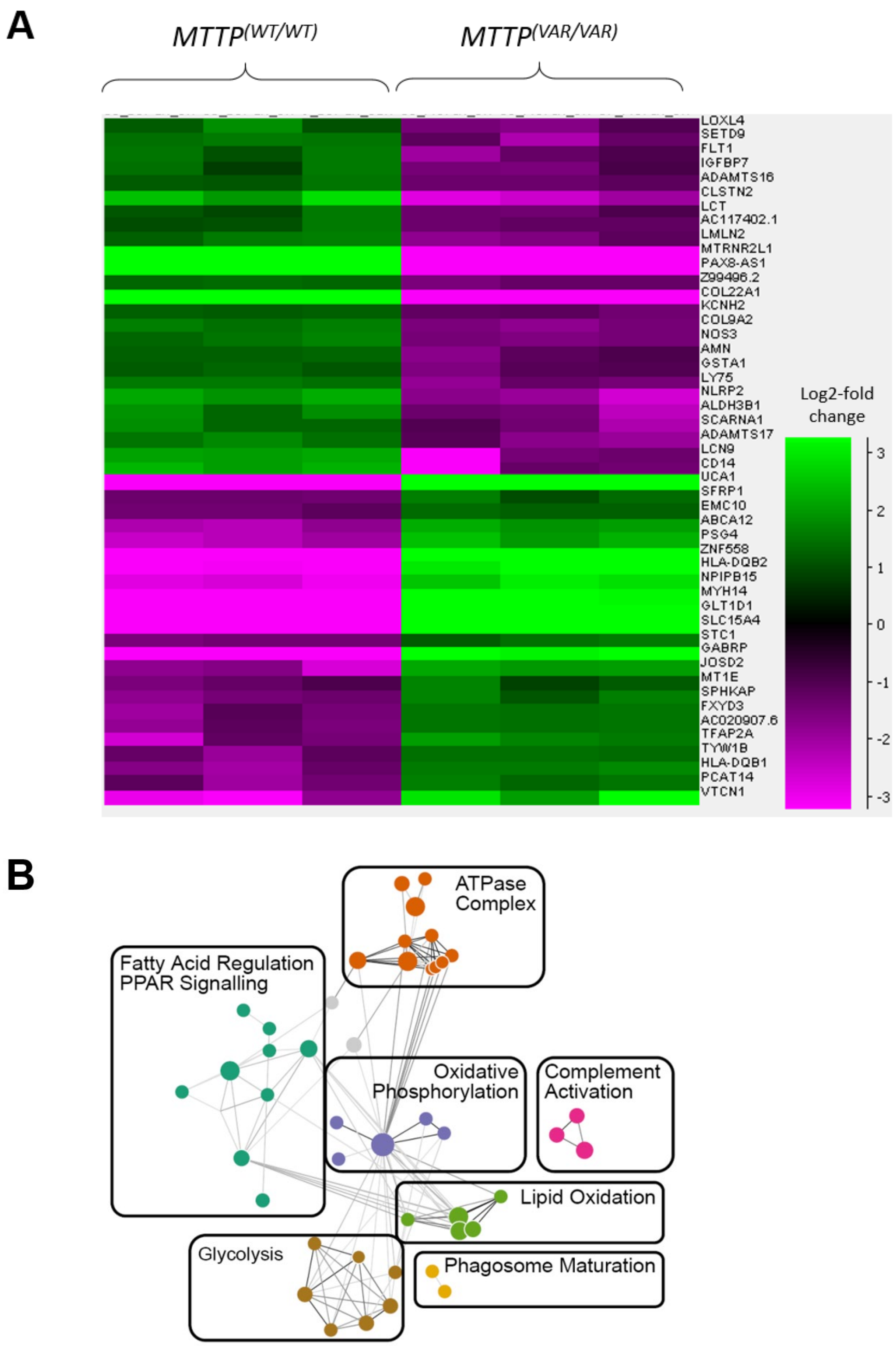

Fig. S9. Comparison of expression patterns in HLCs with MTP564-II and MTP564-TT.
(**A**) Heatmap representing 48 differentially expressed genes with more than 2x log2 fold change in expression, in wild type and MTTP*(VAR/VAR)* hIPSC-derived HLCs (triplicate samples).
(**B**) Giraph plot from quantitative gene set analysis showing gene sets up-regulated in *MTTP*(VAR/VAR) hIPSC-derived HLCs.

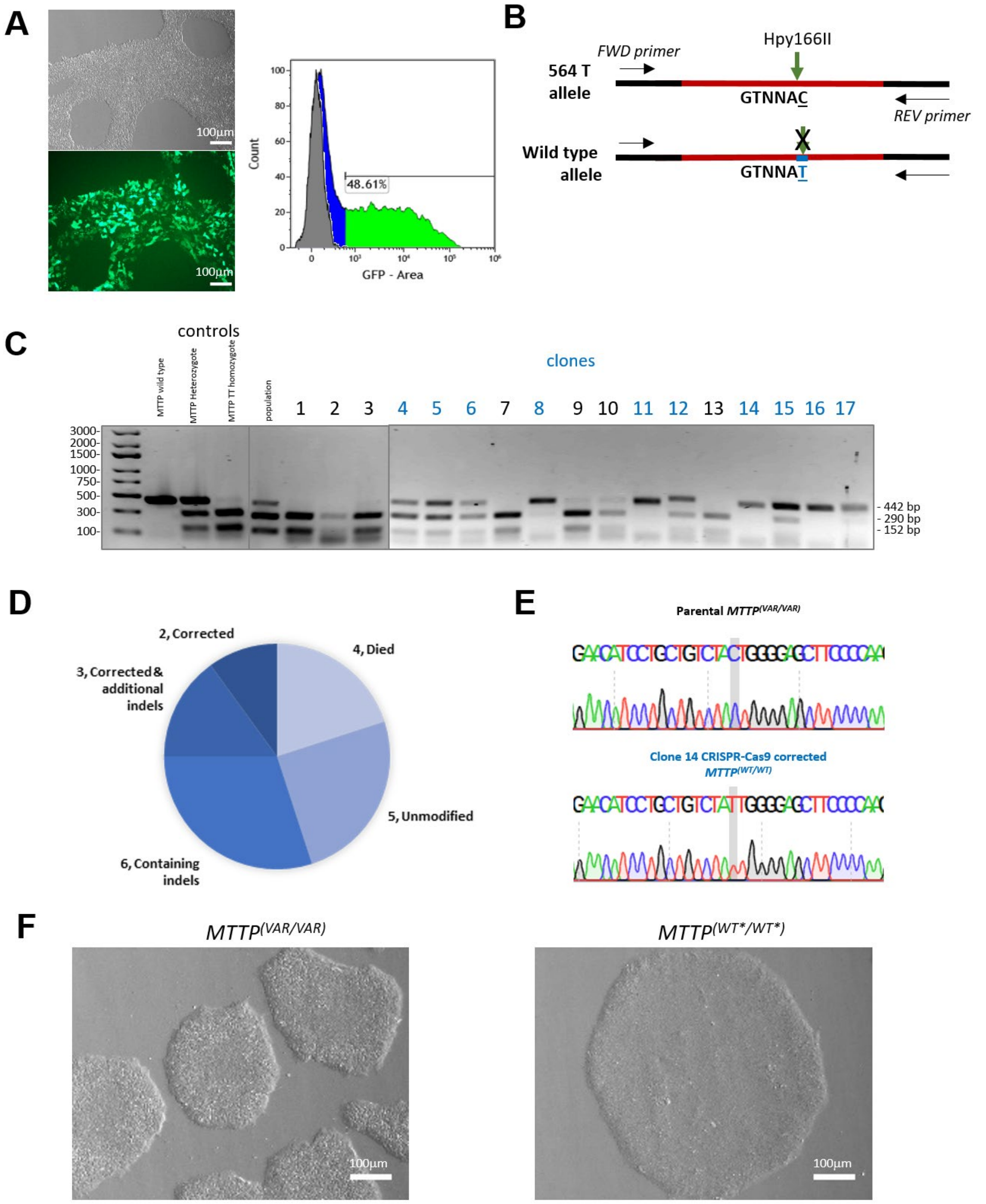

Fig. S10. CRISPR-Cas9 mediated correction of 564TT in the *MTTP*$^{(VAR/VAR)}$ parental line to restore wild type 564II in *MTTP*$^{(WT*/WT*)}$.

(A) Representative phase-contrast and fluorescence microscopy images of MTTP$^{(VAR/VAR)}$ parental line transfected with an EGFP plasmid. Quantification of transfection efficiency by flow cytometry. Untransfected control (grey), sample (blue/green). **(B)** Schematic showing screening strategy for corrected clones. **(C)** Screening and selection of clone 14 by PCR and Hpy166II restriction digestion and agarose gel electrophoresis (O'GeneRuler Express DNA Ladder, Thermofisher). Screening identified 20/39 potentially corrected clones suggesting 51% efficiency. **(D)** Summary of characterisation of clones identified by PCR-RD analysis. Sanger sequencing confirmed 5 clones were unmodified, 6 clones contained indels, 3 were corrected but contained additional indels, giving 5% CRISPR-Cas9 mediated gene-editing efficiency. **(E)** Sanger sequencing chromatograms of parental mutant *MTTP*$^{(VAR/VAR)}$ line and corrected clone 14 *MTTP*$^{(WT*/WT*)}$. **(F)** Representative phase-contrast microscopy images of iPSC cultures for *MTTP*$^{(VAR/VAR)}$ and *MTTP*$^{(WT*/WT*)}$ derived from clone 14.

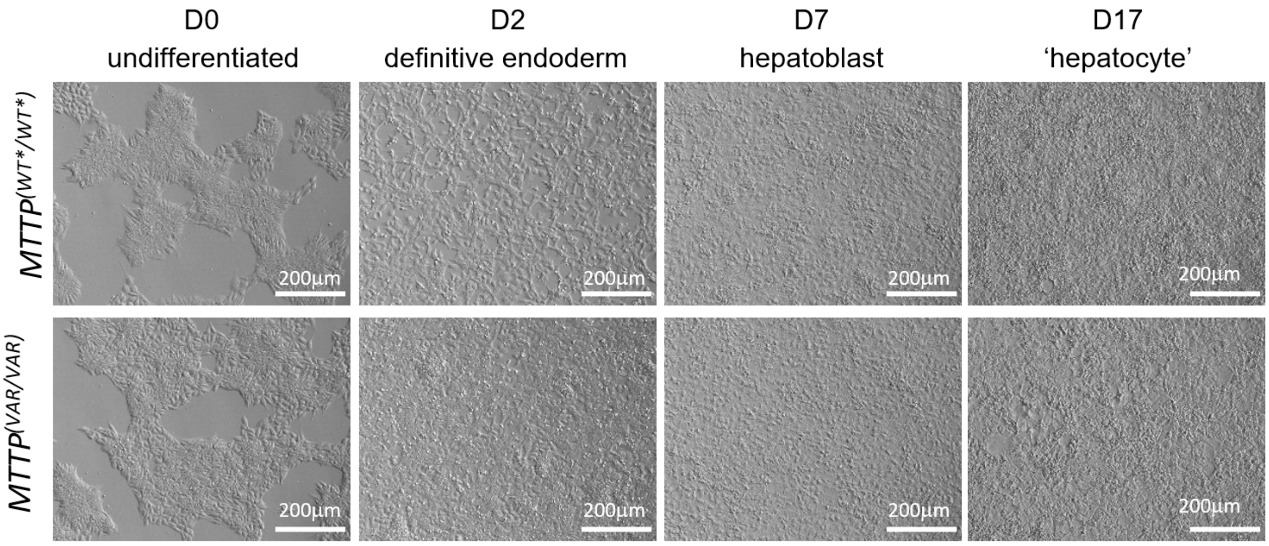

Fig. S11. **Differentiation of CRISPR-Cas9 corrected *MTTP*$^{(WT/WT)}$ and parental *MTTP*$^{(VAR/VAR)}$ hiPSCs into mature HLCs**.

Representative phase-contrast microscopy images of CRISPR-Cas9 corrected *MTTP*$^{(WT/WT)}$ and parental *MTTP*$^{(VAR/VAR)}$ hiPSCs during the differentiation into hiPSC-derived hepatocyte-like cells (HLCs) including undifferentiated cells (Day 0), definitive endoderm (Day 2), hepatoblasts (Day 7) and 'hepatocytes' (Day 17).

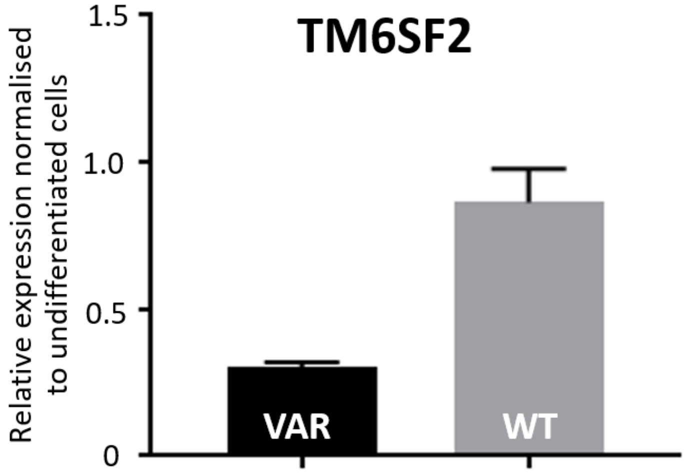

Fig. S12. Assessment of TM6SF2 expression in *MTTP*$^{(VAR/VAR)}$ and restored wild type 564II *MTTP*$^{(WT*/WT*)}$.

Quantification of TM6SF2 mRNA levels by QPCR in *MTTP*$^{(VAR/VAR)}$ and *MTTP*$^{(WT*/WT*)}$ derived hepatocytes.

Supplemental Tables:

Table S1. Characteristics of study participants included in meal response analysis. *PNPLA3* p.I148M and *TM6SF2* p.E167K genotypes are shown. NAFLD, Non-alcoholic fatty liver disease; NASH, non-alcoholic steatohepatitis.

**Family member studied**		**Matched healthy volunteer**		**Matched NAFLD patient**	
**Person ID-Family**	Description	**Person ID-Healthy Volunteer**	Description	**Person ID-NAFLD case**	Description
**F**	♂ 71y,<br>BMI 21.4,<br>MTP564-TT<br>cirrhosis,<br>PNPLA3-IM<br>TM6SF2-EK	**1**	♂ 73y,<br>BMI 29.6,<br>PNPLA3-IM<br>TM6SF2-EE	**2**	♂ 73y,<br>BMI 31.9,<br>NASH + fibrosis<br>PNPLA3-IM<br>TM6SF2-EK
				**3**	♂ 71y,<br>BMI 25.3,<br>NASH + fibrosis<br>PNPLA3-IM<br>TM6SF2-KK
**J**	♀ 57y,<br>BMI 20.3,<br>MTP564-TT<br>healthy<br>transplanted<br>PNPLA3-IM<br>TM6SF2-EE	**4**	♀ 63y,<br>BMI 27.0,<br>PNPLA3-MM<br>TM6SF2-EE	**5**	♀ 66y,<br>BMI 26.6,<br>NASH<br>no fibrosis,<br>PNPLA3-II<br>TM6SF2-EE
**K**	♀ 55y,<br>BMI 24.5,<br>MTP564-IT<br>fatty liver<br>PNPLA3-IM<br>TM6SF2-EK	**6**	♀ 57y,<br>BMI 25.3,<br>PNPLA3-II<br>TM6SF2-EE	**7**	♀ 57y,<br>BMI 41.5,<br>NASH +<br>fibrosis,<br>PNPLA3-MM<br>TM6SF2-EE
**M**	♂ 48y,<br>BMI 25.4,<br>MTP564-II<br>healthy<br>PNPLA3-II<br>TM6SF2-EK	**8**	♂ 57y,<br>BMI 29.5,<br>PNPLA3 II<br>TM6SF2 EK	**9**	♂ 47y,<br>BMI 42.0,<br>NASH +<br>fibrosis,<br>diabetic<br>PNPLA3-II<br>TM6SF2-EE
**Q**	♂ 31y,<br>BMI 22.8,<br>MTP564-IT<br>healthy<br>PNPLA3-MM<br>TM6SF2-EE	**10**	♂ 35y,<br>BMI 29.0,<br>PNPLA3-II<br>TM6SF2-EE	**11**	♂ 30y,<br>BMI 39.9,<br>NASH + fibrosis<br>PNPLA3-MM<br>TM6SF2-EK

Table S2. All RNA-seq and CHIP-seq Sample Search Space (ARCHS4) tissue type and cell type predictions for hIPSC-derived hepatocyte-like cells.

Index		MTP564-II wild type hIPSC derived hepatocyte-like cells					MTP564-TT variant hIPSC derived hepatocyte-like cells				
		Name	P-value	Adjusted P-value	Odds ratio	Combined Score	Name	P-value	Adjusted P-value	Odds ratio	Combined Score
Tissue Type Prediction	1	Liver (bulk tissue)	6.662e-163	7.194e-161	2.72	1017.19	Liver (bulk tissue)	2.363e-171	2.552e-169	2.74	1075.07
	2	Hepatocyte	3.025e-118	1.633e-116	2.44	661.17	Hepatocyte	4.413e-123	2.383e-121	2.45	689.05
	3	Small intestine (bulk tissue)	1.268e-78	4.564e-77	2.15	385.83	Small intestine (bulk tissue)	4.644e-82	1.672e-80	2.16	403.60
	4	Gastric epithelial cell	9.568e-70	2.583e-68	2.08	330.09	Ileum (bulk tissue)	4.053e-68	1.094e-66	2.04	316.85
	5	Ileum (bulk tissue)	4.833e-67	1.044e-65	2.05	313.59	Lung (bulk tissue)	1.365e-61	2.948e-60	1.99	278.23
	6	Lung (bulk tissue)	5.792e-58	1.042e-56	1.97	259.88	Gastric epithelial cell	1.754e-59	3.158e-58	1.97	266.04
	7	Colon (bulk tissue)	1.893e-53	2.921e-52	1.93	234.18	Colon (bulk tissue)	2.340e-53	3.610e-52	1.91	231.44
	8	Gastric tissue (bulk)	4.762e-46	6.428e-45	1.85	193.58	Gastric tissue (bulk)	3.668e-47	4.952e-46	1.85	197.74
	9	Omentum	3.685e-39	4.422e-38	1.78	157.60	Omentum	6.260e-45	7.512e-44	1.83	185.93
	10	Amniotic fluid	1.454e-23	1.570e-22	1.59	83.40	Skin (bulk tissue)	5.090e-28	5.497e-27	1.63	102.71
Cell	1	HEPG2	9.067e-83	1.133e-80	2.16	407.91	HEPG2	1.419e-75	1.774e-73	2.08	358.54
	2	HUH7	5.774e-52	3.609e-50	1.90	223.63	HUH7	5.473e-42	3.421e-49	1.87	216.27

	3	HEP3B	1.702e-41	7.091e-40	1.79	168.03	HEP3B	9.101e-42	3.754e-40	1.78	167.99
	4	CFPAC1	1.049e-38	3.278e-37	1.76	153.90	CFPAC1	2.518e-38	7.870e-37	1.74	150.72
	5	CAPAN1	5.244e-36	1.311e-34	1.73	140.51	MCF10	2.035e-33	5.088e-32	1.69	126.93
	6	A549	1.156e-29	2.409e-28	1.65	110.23	CAPAN1	3.661e-32	7.626e-31	1.67	121.00
	7	BXPC3	7.111e-28	1.270e-26	1.63	102.00	A549	7.477e-32	1.335e-30	1.67	119.54
	8	MCF10	2.724e-25	4.257e-24	1.60	90.38	BXPC3	4.377e-27	6.840e-26	1.61	97.69
	9	RT4	1.230e-23	1.709e-22	1.58	83.09	RT4	1.444e-24	2.006e-23	1.58	86.55
	10	HT29	2.676e-22	3.345e-21	1.56	77.31	HNSCC	1.740e-23	2.175e-22	1.56	81.86

Table S3. Gene set analysis showing terms for all genes differentially expressed between MTP564-II wild type and MTP564-TT variant hIPSC-derived hepatocyte-like cells.

source	term_name	term_id	adjusted_p_value	negative_log10_of_adjusted_p_value
REAC	Extracellular matrix organization	REAC:R-HSA-1474244	4.87E-07	6.312667258
GO:CC	extracellular matrix	GO:0031012	2.85941E-06	5.543723748
REAC	Degradation of the extracellular matrix	REAC:R-HSA-1474228	2.43072E-05	4.614264431
GO:BP	extracellular matrix organization	GO:0030198	0.000831626	3.080071732
GO:BP	extracellular structure organization	GO:0043062	0.000894227	3.048552438
REAC	Collagen formation	REAC:R-HSA-1474290	0.003136295	2.503583115
GO:BP	digestion	GO:0007586	0.004959299	2.304579689
GO:CC	collagen-containing extracellular matrix	GO:0062023	0.00516165	2.287211438
KEGG	Protein digestion and absorption	KEGG:04974	0.006978005	2.156268728
REAC	Collagen biosynthesis and modifying enzymes	REAC:R-HSA-1650814	0.014030082	1.852939779
KEGG	ECM-receptor interaction	KEGG:04512	0.014057765	1.852083733
REAC	ECM proteoglycans	REAC:R-HSA-3000178	0.016110075	1.792902441
TF	Factor: slug; motif: NRCAGGTGCR; match class: 1	TF:M12259_1	0.027606305	1.558991718

Table S4. Gene set analysis showing terms for genes upregulated in MTP564-II wild type hIPSC derived hepatocyte-like cells compared to MTP564-TT variant hIPSC-derived hepatocyte-like cells.

source	term_name	term_id	adjusted_p_value	negative_log10_of_adjusted_p_value
GO:CC	midbody	GO:0030496	0.014234541	1.846656538
REAC	Resolution of Sister Chromatid Cohesion	REAC:R-HSA-2500257	0.018667177	1.728921362
REAC	Amplification of signal from unattached kinetochores via a MAD2 inhibitory signal	REAC:R-HSA-141444	0.023621746	1.626688011
REAC	Amplification of signal from the kinetochores	REAC:R-HSA-141424	0.023621746	1.626688011
GO:CC	spindle	GO:0005819	0.026656468	1.5741974

Table S5. Gene set analysis showing terms for genes upregulated in MTP564-TT variant hIPSC derived hepatocyte-like cells compared to MTP564-II wild type hIPSC-derived hepatocyte-like cells.

source	term_name	term_id	adjusted_p_value	negative_log10_of_adjusted_p_value
REAC	Extracellular matrix organization	REAC:R-HSA-1474244	0.000145038	3.838517822
REAC	Degradation of the extracellular matrix	REAC:R-HSA-1474228	0.000353562	3.451534414
REAC	ECM proteoglycans	REAC:R-HSA-3000178	0.002952027	2.529879617
GO:BP	extracellular matrix organization	GO:0030198	0.008885339	2.051326005
GO:BP	extracellular structure organization	GO:0043062	0.009318155	2.030670061
HP	Abnormal cardiovascular system physiology	HP:0011025	0.014922899	1.826146793
KEGG	ECM-receptor interaction	KEGG:04512	0.01921473	1.71636572
KEGG	Focal adhesion	KEGG:04510	0.027077396	1.567393098
WP	Focal Adhesion	WP:WP306	0.030961443	1.509178802
HP	Osteoporosis	HP:0000939	0.038409054	1.415566389
HP	Abnormality of skin physiology	HP:0011122	0.043372457	1.362785975
HP	Abnormality of humoral immunity	HP:0005368	0.043717346	1.35934621
HP	Abnormal vascular physiology	HP:0030163	0.04508747	1.345944131

Supplemental References

Author names in bold designate shared co-first authorship.

[1] Simpson EJ, Debevec T, Eiken O, Mekjavic I, Macdonald IA. PlanHab: the combined and separate effects of 16 days of bed rest and normobaric hypoxic confinement on circulating lipids and indices of insulin sensitivity in healthy men. Journal of applied physiology 2016;120:947-955.

[2] Havel RJ, Eder HA, Bragdon JH. The distribution and chemical composition of ultracentrifugally separated lipoproteins in human serum. The Journal of clinical investigation 1955;34:1345-1353.

[3] **John C, Reeve NF**, Free RC, Williams AT, Ntalla I, Farmaki A-E, et al. Cohort Profile: Extended Cohort for E-health, Environment and DNA (EXCEED). International Journal of Epidemiology 2019;48:678-679j.

[4] Li H, Durbin R. Fast and accurate short read alignment with Burrows-Wheeler transform. Bioinformatics 2009;25:1754-1760.

[5] **Li H, Handsaker B**, Wysoker A, Fennell T, Ruan J, Homer N, et al. The Sequence Alignment/Map format and SAMtools. Bioinformatics 2009;25:2078-2079.

[6] Van der Auwera GA, Carneiro MO, Hartl C, Poplin R, Del Angel G, Levy-Moonshine A, et al. From FastQ data to high confidence variant calls: the Genome Analysis Toolkit best practices pipeline. Curr Protoc Bioinformatics 2013;43:11.10.11-11.10.33.

[7] https://www.internationalgenome.org. Accessed 15 March 2015

[8] ftp://ftp.1000genomes.ebi.ac.uk/vol1/ftp/release/20130502/supporting/hd_genotype_chip/. Accessed 15 March 2015

[9] https://www.ncbi.nlm.nih.gov/snp/. Accessed 15 March 2015.

[10] https://www.sanger.ac.uk/resources/downloads/human/hapmap3.html. Accessed 15 March 2014

[11] https://evs.gs.washington.edu/EVS/. Accessed 13 March 2015.

[12] **Chambers JC, Abbott J, Zhang W, Turro E,** Scott WR, Tan ST, et al. The South Asian genome. PloS one 2014;9:e102645.

[13] Wang K, Li M, Hakonarson H. ANNOVAR: functional annotation of genetic variants from high-throughput sequencing data. Nucleic Acids Research 2010;38:e164-e164.

[14] McLaren W, Gil L, Hunt SE, Riat HS, Ritchie GRS, Thormann A, et al. The Ensembl Variant Effect Predictor. Genome Biology 2016;17:122.

[15] Vaser R, Adusumalli S, Leng SN, Sikic M, Ng PC. SIFT missense predictions for genomes. Nat Protoc 2016;11:1-9.

[16] **Adzhubei IA, Schmidt S, Peshkin L,** Ramensky VE, Gerasimova A, Bork P, et al. A method and server for predicting damaging missense mutations. Nat Methods 2010;7:248-249.

[17] **Kircher M, Witten DM,** Jain P, O'Roak BJ, Cooper GM, Shendure J. A general framework for estimating the relative pathogenicity of human genetic variants. Nat Genet 2014;46:310-315.

[18] Ritchie GR, Dunham I, Zeggini E, Flicek P. Functional annotation of noncoding sequence variants. Nat Methods 2014;11:294-296.

[19] Kelley LA, Mezulis S, Yates CM, Wass MN, Sternberg MJE. The Phyre2 web portal for protein modeling, prediction and analysis. Nature protocols 2015;10:845-858.

[20] Yates CM, Filippis I, Kelley LA, Sternberg MJ. SuSPect: enhanced prediction of single amino acid variant (SAV) phenotype using network features. Journal of molecular biology 2014;426:2692-2701.

[21] Grove JI, Thiagarajan P, Astbury S, Harris R, Delahooke T, Guha IN, et al. Analysis of genotyping for predicting liver injury marker, procollagen III in persons at risk of non-alcoholic fatty liver disease. Liver international : official journal of the International Association for the Study of the Liver 2018;38:1832-1838.

[22] Bertero A, Pawlowski M, Ortmann D, Snijders K, Yiangou L, Cardoso de Brito M, et al. Optimized inducible shRNA and CRISPR/Cas9 platforms for in vitro studies of human development using hPSCs. Development 2016;143:4405.

[23] Vallier L, Touboul T, Chng Z, Brimpari M, Hannan N, Millan E, et al. Early cell fate decisions of human embryonic stem cells and mouse epiblast stem cells are controlled by the same signalling pathways. PloS one 2009;4:e6082.

[24] Hannan NR, Fordham RP, Syed YA, Moignard V, Berry A, Bautista R, et al. Generation of multipotent foregut stem cells from human pluripotent stem cells. Stem cell reports 2013;1:293-306.

[25] Ran FA, Hsu PD, Wright J, Agarwala V, Scott DA, Zhang F. Genome engineering using the CRISPR-Cas9 system. Nature protocols 2013;8:2281-2308.

[26] Shen B, Zhang W, Zhang J, Zhou J, Wang J, Chen L, et al. Efficient genome modification by CRISPR-Cas9 nickase with minimal off-target effects. Nature methods 2014;11:399-402.

[27] Priddle H, Allegrucci C, Burridge P, Munoz M, Smith NM, Devlin L, et al. Derivation and characterisation of the human embryonic stem cell lines, NOTT1 and NOTT2. In vitro cellular & developmental biology Animal 2010;46:367-375.

[28] Rimland CA, Tilson SG, Morell CM, Tomaz RA, Lu WY, Adams SE, et al. Regional Differences in Human Biliary Tissues and Corresponding In Vitro-Derived Organoids. Hepatology 2021;73:247-267.

[29] Segeritz CP, Rashid ST, de Brito MC, Serra MP, Ordonez A, Morell CM, et al. hiPSC hepatocyte model demonstrates the role of unfolded protein response and inflammatory networks in α(1)-antitrypsin deficiency. Journal of hepatology 2018;69:851-860.

[30] Kuleshov MV, Jones MR, Rouillard AD, Fernandez NF, Duan Q, Wang Z, et al. Enrichr: a comprehensive gene set enrichment analysis web server 2016 update. Nucleic acids research 2016;44:W90-97.
